# Liver transcriptome analysis reveals PSC-attributed gene set associated with fibrosis progression

**DOI:** 10.1016/j.jhepr.2024.101267

**Published:** 2024-11-12

**Authors:** Alena Laschtowitz, Eric L. Lindberg, Anna-Maria Liebhoff, Laura Anne Liebig, Christian Casar, Silja Steinmann, Adrien Guillot, Jun Xu, Dorothee Schwinge, Michael Trauner, Ansgar Wilhelm Lohse, Stefan Bonn, Norbert Hübner, Christoph Schramm

**Affiliations:** 1 Department of Medicine, University Medical Center Hamburg-Eppendorf, Hamburg, Germany; 2 Department of Hepatology and Gastroenterology, Charité - Universitätsmedizin Berlin, Berlin, Germany; 3 Cardiovascular and Metabolic Sciences, Max-Delbrueck-Center for Molecular Medicine, Berlin, Germany; 4 Berlin Institute of Health (BIH), Berlin, Germany; 5 European Reference Network for Hepatological Diseases (ERN-RARE LIVER), Hamburg, Germany; 6 Department of Medicine I, LMU University Hospital, LMU Munich, Munich, Germany; 7 Gene Center, Department of Biochemistry, Ludwig Maximilians Universität, Munich, Germany; 8 Institute of Medical Systems Biology, Center for Molecular Neurobiology, University Medical Center Hamburg-Eppendorf, Hamburg, Germany; 9 Bioinformatics Core, University Medical Center Hamburg-Eppendorf; Hamburg, Germany; 10 Department of Biomarker Sciences, Gilead Sciences Inc., San Mateo, California, United States of America; 11 Division of Gastroenterology and Hepatology, Department of Medicine III, Medical University of Vienna, Vienna, Austria; 12 German Center for Cardiovascular Research (DZHK), partner site Berlin, Berlin, Germany; 13 Charité - Universitätsmedizin Berlin, Berlin, Germany; 14 Martin-Zeitz-Center for Rare Diseases, University Medical Center Hamburg-Eppendorf, Hamburg, Germany; 15 Hamburg Center for Translational Immunology (HCTI), Hamburg, Germany

**Keywords:** Primary sclerosing cholangitis, Biliary fibrosis, Bulk RNA-sequencing, Liver transcriptome

## Abstract

**Background & Aims:**

Primary sclerosing cholangitis (PSC) is a chronic heterogenous cholangiopathy with unknown etiology where chronic inflammation of the bile ducts leads to multifocal biliary strictures and biliary fibrosis with consecutive cirrhosis development. We here aimed to identify a PSC-specific gene signature associated with biliary fibrosis development.

**Methods:**

We performed RNA-sequencing of 47 liver biopsies from people with PSC (n = 16), primary biliary cholangitis (PBC, n = 15), and metabolic dysfunction-associated steatotic liver disease (MASLD, n = 16) with different fibrosis stages to identify a PSC-specific gene signature associated with biliary fibrosis progression. For validation, we compared an external transcriptome data set of liver biopsies from people with PSC (n = 73) with different fibrosis stages (baseline samples from NCT01672853).

**Results:**

Differential gene expression analysis of the liver transcriptome from patients with PSC with advanced *vs.* early fibrosis revealed 431 genes associated with fibrosis development. Of those, 367 were identified as PSC-associated when compared with PBC or MASLD. Validation against an external data set of 73 liver biopsies from patients with PSC with different fibrosis stages led to a condensed set of 150 (out of 367) differentially expressed genes. Cell type specificity assignment of those genes by using published single-cell RNA-Seq data revealed genetic disease drivers expressed by cholangiocytes (e.g. *CXCL1, SPP1*), fibroblasts, innate, and adaptive immune cells while deconvolution along fibrosis progression of the PSC, PBC, and MASLD samples highlighted an early involvement of macrophage- and neutrophil-associated genes in PSC fibrosis.

**Conclusions:**

We reveal a PSC-attributed gene signature associated with biliary fibrosis development that may enable the identification of potential new biomarkers and therapeutic targets in PSC-related fibrogenesis.

**Impact and implications:**

Primary sclerosing cholangitis (PSC) is an inflammatory liver disease that is characterized by multifocal inflammation of bile ducts and subsequent biliary fibrosis. Herein, we identify a PSC-specific gene set of biliary fibrosis progression attributing to a uniquely complex milieu of different cell types, including innate and adaptive immune cells while neutrophils and macrophages showed an earlier involvement in fibrosis initiation in PSC in contrast to PBC and metabolic dysfunction-associated steatotic liver disease. Thus, our unbiased approach lays an important groundwork for further mechanistic studies for research into PSC-specific fibrosis.

## Highlights

• Bulk RNA-Seq of liver biopsies revealed a PSC-attributed gene set associated with biliary fibrosis progression.
• External single-cell RNA-Seq data suggested the involvement of cholangiocytes, but also innate and adaptive immune cells in PSC fibrosis.
• Time course analysis indicated that macrophage- and neutrophil-associated genes are enriched in early fibrosis stages in PSC.

## Graphical abstract

**Figure:**
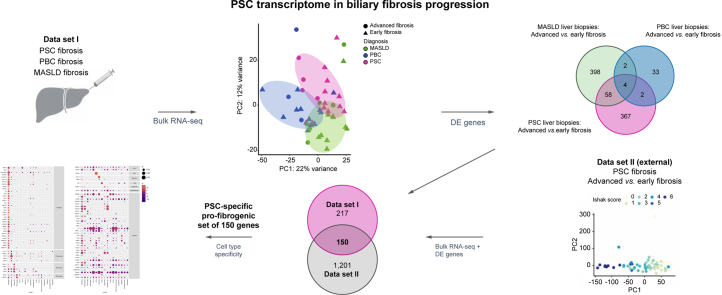

## Introduction

Primary sclerosing cholangitis (PSC) is a chronic heterogenous cholangiopathy that is characterized by multifocal inflammation of bile ducts and subsequent biliary fibrosis.^1^ Progression of fibrosis leads to end-stage liver disease in a considerable number of patients and together with hepatobiliary malignancy represents the main cause of mortality in PSC. To date, no clinically approved antifibrotic or causal treatments are available and liver transplantation remains the only effective therapy.^2^ Pre-clinical rodent models including bile duct ligation and Mdr2-knockout mice have been developed to discover the mechanisms underlying chronic cholestasis and subsequent fibrosis. However, translation to human PSC has been hampered because of the many drawbacks of murine models based on the lack of understanding of disease pathogenesis in PSC.^3^ Recent RNA-sequencing (RNA-Seq) studies aimed to decipher the complex cell composition in cirrhosis identifying multiple cell types of mesenchymal, endothelial, and myeloid origin.[4], [5], [6] However, those studies mostly focused on end-stage liver disease and neglected to examine earlier stages of fibrosis while often failing to include other non-PSC control cohorts.

In the current study, we used an unbiased RNA-Seq approach to identify a PSC-specific pro-fibrogenic gene signature. Thinking of fibrosis development as a therapeutic target, it is important to assess the progression from early fibrosis stages to advanced stage disease. We therefore analyzed the liver transcriptome in people with PSC with different stages of fibrosis and added biliary disease controls (primary biliary cholangitis [PBC]) and non-biliary controls (metabolic dysfunction-associated liver disease [MASLD]), to identify key drivers for PSC-specific biliary fibrosis progression.

## Patients and methods

### Patient population and liver samples

In our retrospective study 47 liver biopsies from people with PSC, PBC and MASLD who had undergone liver biopsy between 2011 and 2016 at the University Medical Center Hamburg-Eppendorf, Germany were included for RNA-Seq analysis. Diagnosis of the underlying chronic liver disease was based on clinical, biochemical, serological, radiological, and histopathological findings according to current guidelines[7], [8], [9] and biopsies were taken as part of routine clinical practice according to the standard procedure in our center via TruCut needle biopsy (TruCut, South Jordan, UT, USA) during a mini-laparoscopic procedure. Liver samples of all etiologies were categorized as early (Desmet/Scheuer stage 0–2/4) or advanced fibrosis (3–4/4) by local pathologists using the Desmet classification for better comparability.^10^ Liver tissue was stored in liquid nitrogen for further analysis.

For the qPCR validation liver tissue from explanted livers of patients with PSC cirrhosis who had undergone liver transplantation at the University Medical Center Hamburg-Eppendorf between 2015 and 2018 and from margins of resected liver adenomas that were undertaken at the University Medical Center Hamburg-Eppendorf between 2015 and 2019 were used.

### Clinical and biochemical parameters

We assessed data on clinical, biochemical and serological parameters at the time of liver biopsy. The following laboratory values were assessed: alanine aminotransferase (ALT), aspartate aminotransferase (AST), alkaline phosphatase (ALP), IgG, gamma-glutamyltransferase (γ-GT), albumin, bilirubin, creatinine, hemoglobin, platelet count, and international normalized ratio (INR).

### RNA purification

Total RNA was isolated from frozen liver tissue using the NucleoSpinKit (Macherey-Nagel, Düren, Germany) and complementary DNA (cDNA) was revere-transcribed from total RNA (High capacity cDNA Reverse Transcriptase Kit, applied biosystems, by Thermo Fisher Scientific). Expression was measured using the Kappa probe Fast Universal qPCR mastermix in combination with Taqman probes (applied biosystems, by Thermo Fisher Scientific) that were used for amplification.

### Validation with qPCR

To analyze the gene expression data, the ΔCt (delta Ct) method was used, whereby the Ct values of the target genes were normalized to the expression levels of the housekeeping gene Hypoxanthine Guanine Phosphoribosyltransferase (HPRT). This normalization approach allows for the comparison and interpretation of relative gene expression levels across different samples. The resulting values were reported as fold changes, indicating the increase or decrease in expression relative to the control samples. The fold changes were calculated using formula 2^(-ΔΔCt)^, which provides a quantitative measure of the relative changes in gene expression levels compared with the control group.

### RNA-Seq and analyses

RNA quality was assessed using Bioanalyzer, and samples with and RNA integrity number >7 were included for RNA-Seq. Up to 1 μg RNA was used to synthesize mRNA libraries using TruSeq stranded mRNA library Preparation Kit (Illumina) on a Hiseq 4000 system (Illumina). We used FastQC (version 0.11.5, Babraham Institute, Babraham, UK) for a general quality check of the raw fastq files. TruSeq2-PE adapter and low quality read trimming was performed with Trimmomatic^11^ (version 0.36) using the options ILLUMINACLIP:TruSeq2-PE.fa:2:30:10:2:falseSLIDINGWINDOW:4:15. Subsequently, the reads were aligned against the ensemble 87 reference genome and ensemble 87 reference annotation with STAR (version 2.7.3a).^12^ On average read depth for the 47 samples was 40.310.540 (range: 8.318.152–61.856.384) and on average 92.76% (range: 79.27–95.88%) of the reads were uniquely mapped to the reference genome.

Further analysis was performed with R (version 3.6.3, R Foundation for Statistical Computing, Vienna, Austria). To filter the data, a threshold of ≥10 counts in all samples was set. We used DESeq2 for normalization and differential gene expression analysis.^13^ Data were inspected for possible confounding effects using principal component analysis (PCA) based on regularized logarithm transformed counts. We compared different diseases and early *vs.* advanced stages of fibrosis across different etiologies including covariates for sex. We corrected for multiple testing with the Benjamini–Hochberg method. Adjusted *p* values of ≤0.05 were considered significantly different and an absolute cut-off log_2_ foldchange (log_2_FC) of 1 was set.

### Pathway analysis and enrichment analysis

Pathway and enrichment analysis was performed using KEGG pathway analysis or GO term analysis via the ClusterProfiler package in the R environment. If reasonable, the simplify-function was used to remove redundancy of enriched GO terms.

### Cell type mapping

Cell type mapping for the identified genes was determined by using a public available single-cell RNA-Seq data set only from lean people without chronic liver disease.^14^ Dot size represents the fraction of cells within a cell type cluster where transcripts of the gene were detected, whereas only cell types that showed expression of the gene in at least 0.5% of the cell fraction are shown. Color scale illustrates enrichment of expression (foldchange; logFC) in relation to all other cell types. Cell type specificity was defined as 50% higher mRNA expression levels in comparison with cell types with the next highest mRNA expression.

### Cell type mapping along fibrosis progression trajectory

Each gene weight was extracted per principal component (PC) describing the disease progression from fibrosis stages 0–4 for PSC, PBC, and MASLD respectively. For all genes, cell type specificity was calculated based on the fraction of single cell per cell type expressing the gene from a previously published scRNA-Seq study.^14^ To map gene expression signatures onto specific cell types at various stages of fibrosis progression, we normalized the gene expression data from the published scRNA-Seq study by library size, performed a log transformation, and scaled the values for each gene to unit variance and zero mean. We then multiplied these normalized gene expression values by the gene weights in the PC. For each fibrosis-course describing PC, the cell type specificity for each gene was determined and ranked according to its coefficient. The same sign for each PC was chosen, so that negative signs are associated with lower fibrosis scores, whereas positive signs indicate higher fibrosis scores.

### Statistical analysis

Percentages and counts are given for categorical data. Median values with the corresponding range were calculated for continuous data. To test for differences between groups, non-parametric tests, including the Wilcoxon signed rank test, were performed. A comparison of categorical data between groups was performed using Pearson’s Χ^2^ test. All *p* values were two-tailed, a *p* value <0.05 was considered statistically significant. Figure design and statistical testing were carried out using R version 3.6.0 and R Studio version 1.2.1335.

### Study approval

Informed written consent was obtained from each person. The study has been approved by the local ethics committee (ethics number PV4081).

## Results

### Patient characteristics at time of liver biopsy

In total, 47 liver samples from people with PSC (n = 16), PBC (n = 15), and MASLD (n = 16) with different fibrosis stages were included in the RNA-Seq analysis. Clinical characteristics of all patients are displayed in Table 1. Distribution of sex was different between people with PSC and PBC (PSC: 19% female, PBC: 87% female, *p* <0.001), and age differed between people with PSC and PBC (PSC median 37 years *vs.* PBC median 53 years, *p* <0.001) as well as MASLD (PSC median 37 years *vs.* MASLD median 49.5 years, *p* <0.001).

**Table 1:** Clinical characteristics at the time of liver biopsy.

Parameters	PSC (n = 16)	PBC (n = 15)	MASLD (n = 16)	*p* value∗	
				PSC *vs.* PBC	PSC *vs.* MASLD
Sex (female), n (%)	3 (19)	13 (87)	8 (50)	<0.001	0.137
Age (range), years	37 (18–59)	53 (40–82)	49.5 (23–73)	<0.001	0.01
Hemoglobin (range), g/dl	14.4 (10.4–16.3)	13.1 (9.5–15.9)	15.1 (12.5–16.3)	0.135	0.156
Platelets (range), 10^9^/L	248 (82–448)	241 (125–448)	243 (48–368)	0.989	0.269
Albumin (range), g/L	38 (24–43)	35 (21–41)	38 (37–42)	0.173	0.39
Bilirubin (range), mg/dl	1 (0.3–9.9)	1.5 (0.4–3)	0.5 (0.3–2.3)	0.303	0.533
AST (range), U/L	88 (16–263)	74 (20–487)	40.5 (21–142)	0.489	0.037
ALT (range), U/L	100 (19–723)	61 (17–515)	72 (26–290)	0.406	0.052
γ-GT (range), U/L	450 (62–1,310)	245 (73–839)	117 (30–901)	0.205	0.081
ALP (range), U/L	336 (115–672)	231 (85–746)	96 (54–137)	0.809	<0.001
IgG (range), g/L	17.3 (10.6–23.8)	15.1 (8.7–46.5)	—	0.676	—
Creatinine (range), mg/dl	0.9 (0.4–1)	0.8 (0.6–1.1)	1 (0.7–1.2)	0.919	0.037
INR (range)	1 (0.9–1.3)	1 (0.9–1.1)	1 (0.8–1.4)	0.157	0.594
Fibrosis stage (Desmet *et al.*^15^), n (%)					
0	1 (6)	2 (13)	4 (25)		
1	7 (44)	5 (33)	5 (31)		
2	4 (25)	5 (33)	1 (6)		
3	2 (12.5)	0	4 (25)		
4	2 (12.5)	3 (20)	2 (13)		
Systemic immunosuppressive treatment, n (%)	4 (25)	3 (20)	0	0.679	<0.001
Treatment with UDCA, n (%)	8 (50)	10 (66)	0	0.565	<0.001
Chronic inflammatory bowel disease, n (%)	9 (69)	0	0	<0.001	<0.001

Median values are presented with range in brackets. ∗Continuous variables were compared using Wilcoxon singed rank test. Pearson’s Χ^2^ test was used for comparing percentages. ALT, alanine aminotransferase; ALP, alkaline phosphatase; AST, aspartate aminotransferase; γ-GT, gamma-glutamyltransferase; IgG, immunoglobulin G; INR, international normalized ratio; MASLD, metabolic dysfunction-associated steatotic liver disease; PBC, primary biliary cholangitis; PSC, primary sclerosing cholangitis; UDCA, ursodesoxycholic acid.

### RNA-Seq of liver biopsies reveals etiology-specific pro-fibrogenic gene signatures

**Fig. 1:**
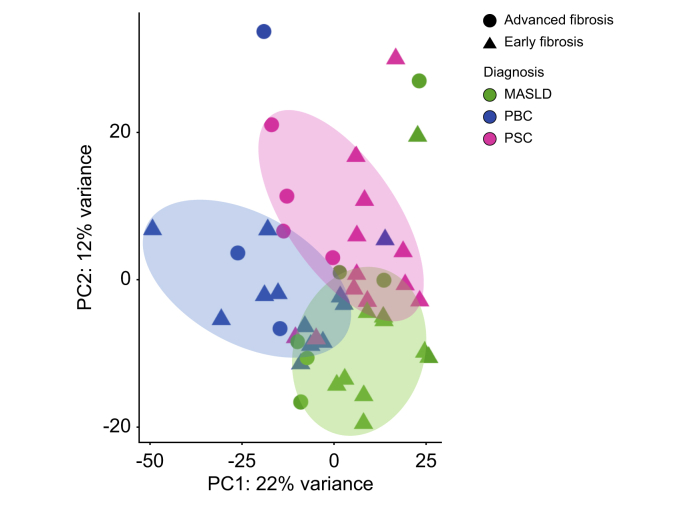
Disease- and sex-based clustering of liver samples of patients with PSC, PBC, and MASLD after RNA-sequencing analysis. Principal component analysis after RNA-sequencing of liver samples of patients with PSC (n = 16), PBC (n = 15) and MASLD (n = 16) with advanced and early fibrosis. Counts of the filtered genes are used. MASLD, metabolic dysfunction-associated steatotic liver disease; PBC, primary biliary cholangitis; PC, principal component; PSC, primary sclerosing cholangitis.

To explore a common gene signature for fibrosis development, we undertook RNA-Seq of liver biopsies from people with PSC, PBC, and MASLD. PCA for all samples revealed a clustering mainly based on the underlying disease and sex, as shown in Fig. 1 and Fig. S1A. Other clinical characteristics such as age, immunosuppression or UDCA intake at the time of biopsy did not show a clear impact on PCA clustering, as shown in our Fig. S1A and B, and could therefore be neglected as covariates. Consequently, we included sex as covariate for our further differential gene expression analyses between the diseases. As expected, categorization of all patient samples into early fibrosis (stages 0–2) and advanced fibrosis (stages 3–4) according to Desmet *et al.*^15^ without considering the underlying etiology did not result in a clear clustering between people with advanced or early fibrosis, as shown in Fig. 1, indicating strong disease-specific pro-fibrogenic mechanisms. Accordingly, we continued our analysis comparing the samples with advanced *vs.* early fibrosis strictly classified by the underlying etiology. Clinical characteristics within the groups are displayed in Table 2.

**Table 2:** Clinical characteristics at the time of liver biopsy of study patients divided into advanced (stage 3–4) and early fibrosis (stage 0–2).

	PSC			PBC			MASLD		
	Advanced fibrosis (n = 4)	Early fibrosis (n = 12)	*p* value∗	Advanced fibrosis (n = 3)	Early fibrosis (n = 12)	*p* value∗	Advanced fibrosis (n = 6)	Early fibrosis (n = 10)	*p* value∗
Sex (female), n (%)	1 (25)	2 (17)	0.712	3 (100)	10 (83)	0.448	3 (50)	5 (50)	1
Age (range), years	37 (24–48)	35 (18–59)	0.969	55 (53–66)	49 (40–82)	0.379	65.5 (48–73)	48.5 (23–60)	0.251
Systemic immunosuppressive treatment, n (%)	1 (25)	4 (36)	0.679	0	3 (25)	0.333	0	0	–
MELD	11.5 (9–14)	–	–	10 (6–10)	–	–	8 (7–15)	–	–
Child–Pugh	6.5 (5–8)	–	–	5 (5–7)	–	–	5 (5–6)		
Presence of varices	1 (25)	–	–	0	–	–	1 (17)	–	–
Episode of decompensation at time of biopsy	0	–	–	0	–	–	1† (17)	–	–
Percentage of steatosis (%)	–	–	–	–	–	–	10 (10)	30 (10–60)	0.037
NAFLD activity score	–	–	–	–	–	–	3 (3–5)	4 (0–5)	0.292

Median values are presented with range in brackets. MASLD, metabolic dysfunction-associated steatotic liver disease; NAFLD, non-alcoholic fatty liver disease; PBC, primary biliary cholangitis; PSC, primary sclerosing cholangitis.

∗ Continuous variables were compared using Wilcoxon singed rank test. Pearson’s Χ^2^ test was used for comparing percentages.

† One patient with ascites and variceal bleeding at time of biopsy (2 weeks around biopsy).

### Identification of a PSC-attributed gene signature in biliary fibrosis

**Fig. 2:**
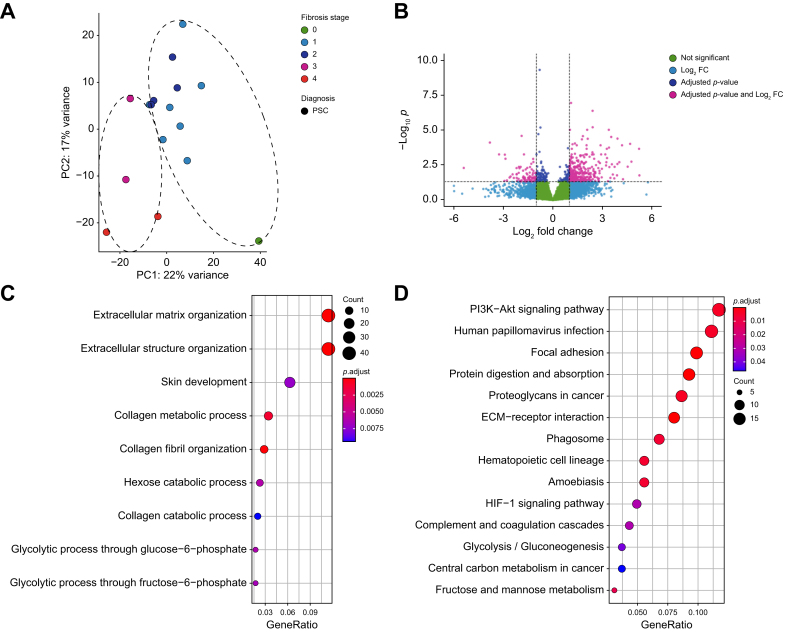
Differential gene expression analysis between advanced and early fibrosis in patients with PSC. (A) Principal component analysis after RNA-sequencing of liver samples of patients with PSC (n = 16) subdivided into advanced (3–4/4) and early (0–2/4) fibrosis. Corrected counts of the filtered genes are used. (B) Volcano plot illustrating genes from the filtered data set, that show significant differential expression between advanced and early fibrosis in 16 patients with PSC. (C) Gene ontology term enrichment analysis for the biological processes of the 431 DEGs between advanced and early fibrosis stage in patients with PSC (n = 16). (D) KEGG pathway analysis of the 431 DEGs between advanced and early fibrosis stage in patients with PSC (n = 16). PC, principal component; PSC, primary sclerosing cholangitis.

To identify a PSC-specific pro-fibrogenic gene set, we categorized the samples of people with PSC into early (stages 0–2) and advanced fibrosis (stages 3–4) and the PCA showed a clear clustering based on the fibrosis score along the first principal component (PC1) as displayed in Fig. 2A. After differential gene expression analysis comparing the PSC samples with advanced fibrosis against early fibrotic PSC samples, 431 differentially expressed genes (DEGs) were revealed (threshold: *p*-adj ≤0.05, log_2_FC ≥|-1|). Results are displayed in a volcano plot in Fig. 2B and in Table S1. Gene ontology enrichment analysis identified expected biological processes such as *extracellular matrix organization* and the *collagen metabolic process* while KEGG pathway analyses revealed cellular processes such as *ECM-receptor interaction* and pathways linked with cancer such as *PI3K-AKT signaling pathway* as displayed in Fig. 2C and D. Similarly, we categorized the samples of patients with PBC or MASLD into early and advanced fibrosis and analyzed them separately. The samples of patients with PBC showed a less stringent clustering based on fibrosis stages in the PCA, as shown in Fig. S2A, and only 41 genes were differentially expressed between advanced and early fibrotic PBC samples, displayed in Table S2, which may relate to the smaller sample size of PBC livers with advanced fibrosis included. However, the samples of people with MASLD showed a clear clustering according to fibrosis stages, as displayed in Fig. S2B with 462 genes being differentially expressed between the two fibrosis categories, as listed in Table S3.

**Fig. 3:**
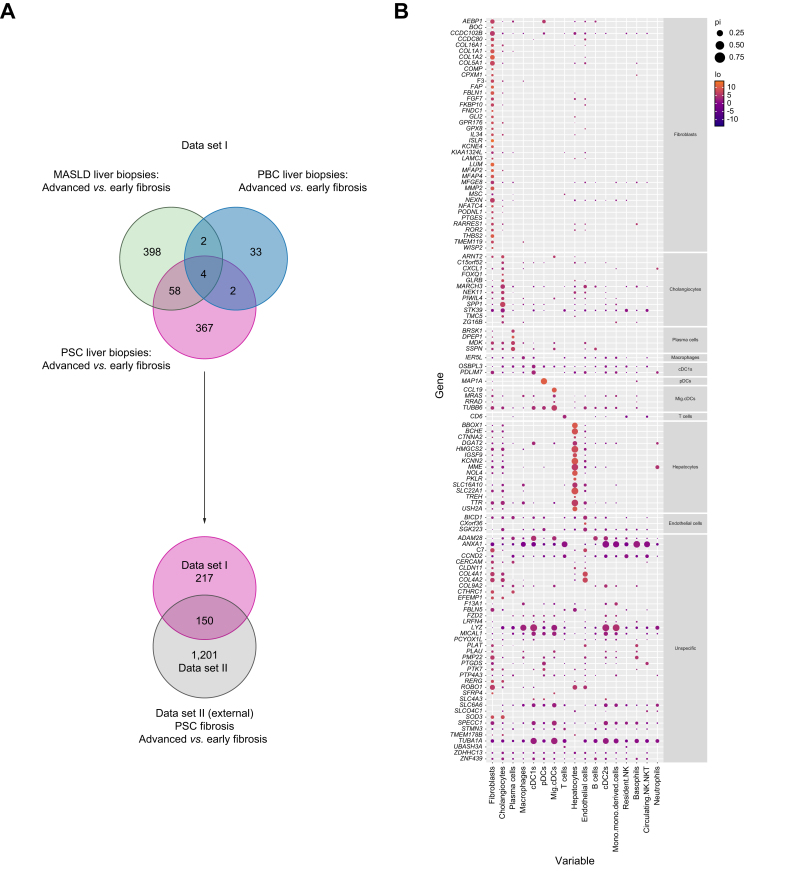
Validated PSC-specific pro-fibrogenic DEGs and their cell type specificity. (A) First Venn diagram showing the overlap and disease-specific DEGs of advanced *vs.* early fibrosis comparison in PSC, PBC, and MASLD in data set I. Second Venn diagram highlighting the overlap of PSC-specific pro-fibrogenic DEGs in data set I and external data set II. (B) Mapping cell type specificity of the 150 PSC-specific pro-fibrogenic genes as overlap from both data sets by using published single-cell RNA-Seq data.^14^ Dot size represents the fraction of nuclei within a cell type where transcripts of the gene were detected, while only cell types that showed expression of the gene in at least 0.5% of the cell type fraction are shown. Color scale illustrates enrichment of expression (foldchange; logFC) compared with all other cell types. Cell type specificity was defined as 50% higher mRNA expression levels in comparison with cell types with next the highest mRNA expression. cDC1, Type-I conventional dendritic cell; DEG, differentially expressed genes; mig. cDC, migratory conventional dendritic cell; MASLD, metabolic dysfunction-associated steatotic liver disease; PBC, primary biliary cholangitis; pDC, plasmacytoid dendritic cell; PSC, primary sclerosing cholangitis.

After identifying the DEGs between advanced *vs.* early fibrosis within each disease cohort separately, we aimed to analyze the unique features and overlaps of the disease-specific pro-fibrogenic gene sets: The majority of PSC-associated differentially expressed pro-fibrogenic genes (367/431 DEGs), were specifically seen in PSC fibrosis progression but not in the control cohorts, as displayed in Fig. 3A illustrating a strong etiology-dependent influence on biliary fibrosis progression. Gene set enrichment analysis of those 367 DEGs identified biological processes such as the extracellular matrix organization, but also leukocyte migration, as shown in Fig. S3.

Only four genes were shown to be pro-fibrogenic genes between all three diseases (*ASPHD1, DMKN, MUC13, ST14*). Amongst them was DMKN, encoding for the protein Dermokine, which had recently been identified as a regulator for hepatic stellate cell (HSC) activation,^16^ the main cellular players in liver fibrosis. Both chronic cholestatic diseases, PBC and PSC, shared additional two genes between their pro-fibrogenic DE gene sets: *SCUBE2* and *SPINK1*.

### External validation confirms PSC-associated pro-fibrogenic gene signature

In a next step, we aimed to validate our findings by means of an external validation cohort. In a recently published paper, Gindin *et al.*^17^ performed bulk RNA-Seq of 74 liver biopsies from people with PSC with different fibrosis stages (baseline samples from NCT01672853) which clustered based on their degree of fibrosis (along PC1). Given the fibrosis score according to the Ishak classification,^10^ we subdivided the samples into advanced fibrosis (4–6/6) and early fibrosis (0–3/6) and performed the differential gene expression analysis with DESeq2 on those samples with sex as covariate according to our first analysis (threshold: *p*-adj ≤0.05, log_2_FC ≥|-1|). In total, 1,351 genes were differentially expressed between PSC samples with advanced *vs.* early fibrosis contributing to biological processes such as *extracellular matrix organization* in the gene ontology enrichment analysis (Fig. S4). We compared this DE gene set to our DEGs generated from liver biopsies of patients with PSC with advanced *vs.* early fibrosis (Table S1). Indeed, 150 DEGs were shared between those two data sets contributing to biological processes such as *extracellular matrix organization* (Fig. S5), and molecular functions as *Wnt-protein binding* in gene ontology enrichment analysis. Accordingly, we found several genes amongst the 150 PSC-specific DEGs, being associated with *Wnt signaling pathway* (e.g. *AEBP1, CTHRC1, FZD2, FZD7, PTK7, ROR2, SFRP4*). Of note, the established fibrosis driver TGF-beta was differentially expressed in the external data set in PSC fibrosis but not in our analysis. Nevertheless, multiple genes known to be involved in TGF-beta signaling were detected within the 150 overlapping DEGs (*CTHRC1, FZD2, GLI2, MFAP2, PDLIM7, PMP22, PTK7, SPECC1*).

Further, according to KEGG pathway analysis, several genes were linked to the *PI3K/Akt pathway* (*BRSK1, CCND2, COL1A1, COL4A1, COL4A2, COMP, CXCL1, FGF7, LAMC3, SLCO4A2, SPP1*), which controls cellular processes such as cell division, survival, and differentiation.

### Cell type specificity assignment deciphers complexity of cell composition in biliary fibrosis

To enable assignment of the validated 150 PSC-specific fibrosis-associated genes to specific cell types, we used published single-cell RNA-Seq data of human livers from lean people without chronic liver disease.^14^ A total of 147 out of 150 genes could be attributed within the single-cell RNA-Seq data to the different cell types as described in the Patients and methods section. Of those, 39 showed fibroblast specificity, as displayed in Fig. 3B, amongst them well characterized genes mostly coding for extracellular matrix (ECM) proteins (e.g. *COL1A1, COL1A2, FBLN1, LUM*) or genes being previously associated to non-PSC liver fibrosis such as *AEBP1*,^18^ *MFAP4*,^19^^,^^20^ and *NFATC4*.^21^

Although 12 genes were assigned to cholangiocytes, other genes could be assigned to innate and adaptive immune cells, notably plasma cells, T lymphocytes, and macrophages and genes attributed to different subsets of dendritic cells such as type 1 conventional dendritic cells (cDC1) and plasmacytoid DCs (pDC), underlining the complex cell composition and interplay in PSC-associated biliary fibrosis progression. For a further 38 genes, a cell type-specific assignment was not easily possible, as the expression was detectable in several cell types and we decided to label them as unspecific.

**Fig. 4:**
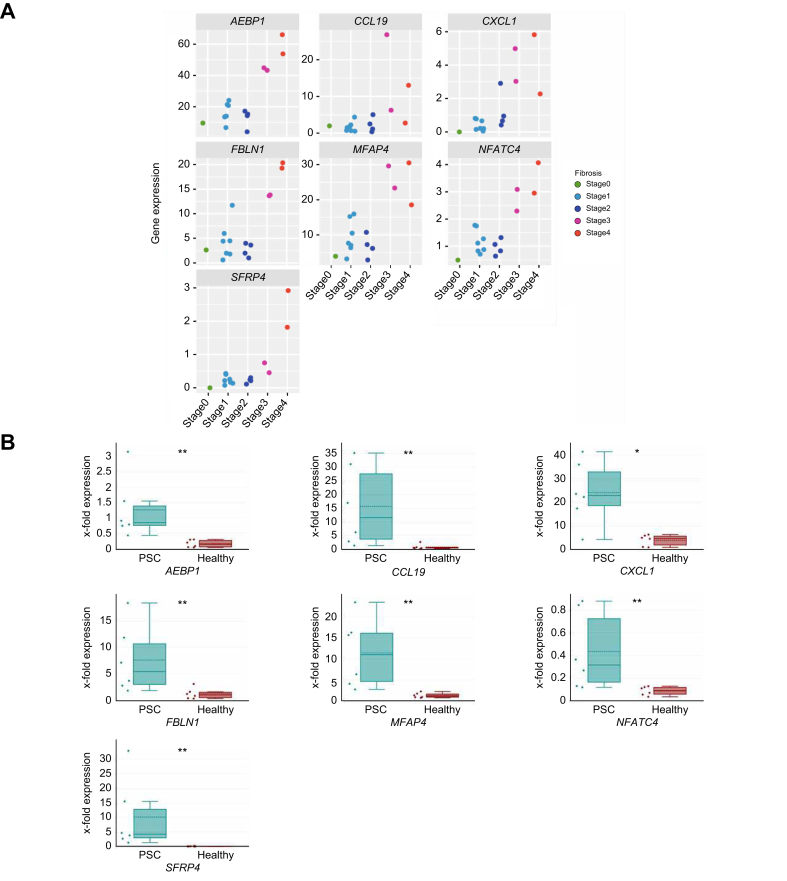
Gene expression along fibrosis progression and validation via qPCR of genes associated with PSC-specific fibrosis progression. (A) Display of the normalized counts (rpm, reads per million) of seven genes from each PSC liver biopsy with the according fibrosis stage. (B) Boxplots representing the gene expression of different genes in liver tissue from persons with PSC cirrhosis (n = 6) or healthy controls (n = 6). Horizontal bars indicating median and IQR, unpaired two-sided Wilcoxon test was performed (∗*p* ≤0.05, ∗∗*p* ≤0.01). PSC, primary sclerosing cholangitis.

Next, we identified those seven out of 150 genes with a steady increase in expression across the PSC fibrosis stages, as shown in Fig. 4A: *AEBP1, CCL19, CXCL1, FBLN1, MFAP4, NFATC4*, and *SFRP4*. The qPCR analysis of liver tissue samples from an independent cohort of persons with PSC cirrhosis (n = 6) and healthy controls (n = 6) validated an upregulated gene expression as displayed in Fig. 4B.

### Macrophage-, neutrophil-, and hepatocyte-associated genes are enriched in PSC fibrosis initiation

**Fig. 5:**
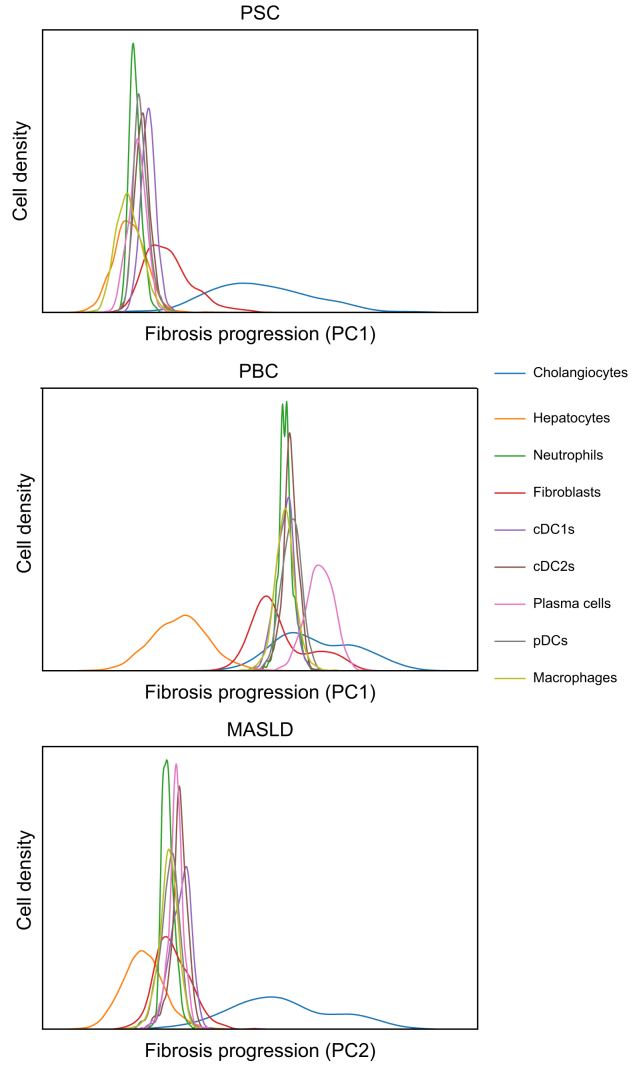
Cell type-specific enrichment over the time course of fibrosis development. Density distributions show cell type scoring along the fibrosis trajectories in PSC, PBC, or MASLD as obtained from the principal component analysis as displayed in Fig. 2A for PSC (along PC1), Fig. S2A for PBC (along PC1) and Fig. S2B for MASLD (along PC2). cDC1, Type-I conventional dendritic cell; mig. cDC, migratory conventional dendritic cell; MASLD, metabolic dysfunction-associated steatotic liver disease; PBC, primary biliary cholangitis; PC, principal component; pDC, plasmacytoid dendritic cell; PSC, primary sclerosing cholangitis.

Considering the low overlap of DEGs from advanced to early fibrosis between PSC, PBC, and MASLD, we wanted to investigate potential cellular drivers of fibrogenesis in PSC by assigning cell type specificity for the enriched genes during fibrosis progression within each disease cohort. For this aim, we first looked at the PCA of the three different diseases and noticed that the fibrosis progression was mostly attributed to the PC1 in PSC and PBC (Fig. 2A and Fig. S2A) and to the PC2 in MASLD (Fig. S2B). We extracted the gene weights of the PCs, respectively, as described above in the methods section and subsequently determined the enrichment of cell type specific gene signatures during fibrosis progression. Finally, we ended up with a density plot showing the assumed contribution of cell types using gene weights during fibrosis progression in PSC, PBC, and MASLD in comparison (Fig. 5). While in early phases of fibrosis in PBC and MASLD mainly hepatocyte-specific genes were enriched, in PSC also macrophages and neutrophils were already assigned at earlier time points, which may indicate an important role of macrophages and neutrophils in PSC fibrosis initiation. Our analyses also suggest an earlier contribution of fibroblast-specific genes in PBC compared with PSC, while in PBC, plasma cell-specific genes appear to be enriched rather late in fibrosis compared with PSC and MASLD. A common factor of all three diseases was that cholangiocyte-specific genes accumulate in late fibrosis stages.

## Discussion

Development of biliary fibrosis with progression to cirrhosis is one of the major life-limiting consequences of PSC.^1^^,^^22^ It is hypothesized that PSC fibrosis is driven by a complex interplay between inflammatory and parenchymal cells as hepatocytes and cholangiocytes, but to date, the detailed underlying pathomechanisms leading from initial insults to peribiliary fibrosis with subsequent cirrhosis development are still unknown.^1^ Recently, several studies have used extensive proteomic and transcriptomic analyses at the single-cell level to demonstrate the complexity and heterogeneity of immune cell composition and their potential interaction with parenchymal and non-parenchymal cells in chronic liver injury and fibrosis.[4], [5], [6]^,^^14^^,^^23^ However, these studies mostly focused on advanced fibrosis and often lacked appropriate control cohorts. By incorporating all stages of PSC fibrosis and comparing with fibrosis development in PBC and MASLD (Fig. 1), our bulk RNA-Seq study provides an unbiased comprehensive transcriptome data set identifying 367 DEGs associated with PSC fibrosis progression (Fig. 2). Further, we validated our analysis with the help of an external, independent dataset of PSC liver-derived transcriptomes thus being able to present a condensed set of 150 genes associated with PSC-specific fibrosis progression. Using published single-cell data, we attributed those genes to specific cell types (Fig. 3). Accordingly, this analysis highlighted the complexity and heterogeneity of cell type composition in PSC fibrosis: Besides cholangiocyte- and fibroblast-specific genes, other genes attributed to plasma cells, T lymphocytes, macrophages and several dendritic cell subtypes. Finally, we compared the contribution of genes along fibrosis progression combined with cell type annotation analysis between PSC, PBC, and MASLD. This indicates an early involvement of macrophages, neutrophils, and hepatocytes in the initiation phase of PSC fibrosis, whereas in PBC and MASLD hepatocyte-associated genes were enriched during early stages of fibrosis (Fig. 5).

The implementation of an external validation cohort led to a condensed data set of 150 DEGs associated with PSC fibrosis. Our analyses provide compelling evidence for the involvement of various signaling pathways such as *Wnt* and *PI3K/Akt*, which represent promising targets for future mechanistic studies. The latter appears to be particularly important for macrophage differentiation in chronic liver disease as recently elucidated.^24^ Accordingly, our data suggest TGF-beta involvement in PSC fibrosis. TGF-beta interacts with the *canonical Wnt/beta-catenin pathway*, whose selective inhibition was shown to decrease inflammatory processes and to reduce growth of activated HSCs and collagen synthesis, thus decelerating the progression of liver fibrosis.^25^ The gene *LOXL2*, which promotes collagen crosslinking in liver fibrogenesis,^26^ was found as differentially expressed in our PSC-specific gene set but not in the external cohort. This may be because of differences in sample acquisition and sequencing performed.

The annotation of those genes to the different cell types deciphered the potential cell types involved in PSC fibrosis and underlined the complexity of the cellular landscape in PSC fibrosis development. Twelve genes were assigned to cholangiocytes with *SPP1* as a well-known marker for activated cholangiocytes. *SPP1* encodes the chemokine osteopontin and has been linked to hepatic fibrosis via activation of HSC^26^ but was recently also shown as marker for recruited lipid-associated macrophages,^27^ underlining the important role of the heterogenous population of myeloid cells in fibrosis progression.

Although cell type-specific assignment was not possible for several genes, some of them had already been attributed to non-PSC liver fibrosis such as *CTHRC1*^28^ or to non-liver fibrosis such as *SFRP4*.^29^^,^^30^ We also found *TMEM178B*, which has recently been discovered as being upregulated in biliary fibrosis in people with biliary atresia.^31^

Moreover, our analyses shed light on new aspects of immune cell populations possibly involved. Our group could recently show that type 2 conventional dendritic cells (cDC2s) may play a role in cholangitis pathogenesis.^32^ Interestingly, the present data herein suggest that in PSC fibrosis, also pDCs, conventional DCs (cDC1), and migratory conventional DCs (mig. cDC) may be involved in fibrosis progression.

Macrophage- and neutrophil-associated genes seemed to be involved already at earlier time points of fibrogenesis in PSC as compared with PBC and MASLD. Recently published data from Guillot *et al.*^23^ may support this finding. Further, Govaere *et al.*^33^ found that in PSC the peribiliary invasion with macrophages occurred already in the early stages of PSC, in contrast to chronic HCV hepatitis. Neutrophils have pathogenic and protective effects in liver fibrosis: they secrete IL-17, thereby upregulating the expression of the TGF-beta receptor in HSCs.^34^ IL-17A promotes the recruitment of neutrophils in the liver and favors liver fibrosis in the model of bile duct ligation in mice.^35^ In patients with PSC, it has recently been shown that neutrophils infiltrate the biliary microenvironment.^36^ Accordingly, we found *CXCL1* increased in PSC fibrosis progression which is known to be involved in the recruitment especially of neutrophils and which has recently been shown to participate in early inflammatory responses and biliary proliferation via the inflammatory CXCL1–CXCR2–neutrophil axis induced by Hedgehog signaling in a mouse model of extrahepatic bile duct ligation.^37^ Additionally, it has been described that CXCL1 expression can be regulated by TH17 cells which are increased in PSC.^38^^,^^39^ In addition, we found *DPEP1* to be upregulated in PSC fibrotic livers, which may also have a role in neutrophil recruitment in lungs and liver.^40^ Taken together, our results suggest the contribution of neutrophils in PSC fibrosis initiation.

Notably, there was small overlap of genes associated with fibrosis progression between PSC, PBC, or MASLD, highlighting the strong etiology-specific mechanisms of fibrogenesis. Nevertheless, PSC and PBC shared two further pro-fibrogenic genes: *SPINK1* and *SCUBE2*. The former has been attributed to chronic pancreatitis,^41^ idiopathic pulmonary fibrosis,^42^ and hepatocellular carcinoma.^43^ The latter belongs to a secreted and membrane-associated multi-domain protein family which was found to play a role in the Hedgehog signaling pathway, a critical regulator in liver fibrosis.^44^ Interestingly, in a mouse model of cholestasis, Hedgehog signaling was found to demarcate a niche of fibrogenic peribiliary mesenchymal cells, indicating that the pathway might play an important role in cholestatic liver fibrosis.^22^^,^^45^ Nevertheless, the small overlap between PSC- and PBC-induced fibrotic gene signatures might not only be caused by already known differences in pathomechanisms – the typical periductular fibrosis in PSC in contrast to the lymphoplasmacellular infiltrates initiating bile duct damage in the portal tracts of PBC livers^46^ – but also attributable to a small sample size of PBC samples with advanced fibrosis.

Several strengths and limitations of our study need to be mentioned. Our analysis provides an unbiased approach that identifies the important gene signatures of biliary fibrosis development and offers the possibility to focus on a PSC-related fibrosis gene set by including different comparison groups and an external validation cohort. Nevertheless, not all fibrosis stages could be equally represented in the PBC group, with only two samples with advanced fibrosis, which may contribute to the small overlap of the DEGs between PSC and PBC fibrosis progression. In addition, the relatively small overlap of the two different data sets is striking, which is mainly attributable to technical differences (e.g. biopsy technique, sequencing technique, *etc*.). Although we provide a method for the interpretation of unbiased bulk RNA-Seq data that allows the assignment of genes to specific cell types, our cell type assignment analysis is based on the fact that the reference data underweigh hepatocyte and cholangiocyte populations because of the underlying single-cell RNA-Seq technique and that data derives from liver samples of people without chronic liver diseases.^14^ A similar bias is also present in the PCA of bulk RNA-Seq data, with gene expression values of lowly abundant cell types less included compared with higher abundant cell types such as hepatocytes.

Moreover, our bulk RNA-Seq data only measures the average expression level of a population of cells, information on the heterogeneity of the cell population is unavailable. Therefore, further unbiased single-cell studies, such as single nuclei RNA-Seq, are needed to study the role of rare immune cell populations and parenchymal cells at different fibrosis stages whereas spatial RNA-Seq is needed to determine the interaction of the different cell types. Lastly, our analysis is restricted to mRNA expression data which require validation at the protein level. Given the small amount of tissue obtained by needle biopsy in our study, no further material was available for validation of protein expression.

In summary, we provide an unbiased study of genes expressed in human livers at different fibrosis stages. We reveal PSC-associated gene signatures of fibrosis as a resource that will enable validation studies, as well as mechanistic studies needed to understand the differences and similarities of fibrosis progression between different liver diseases.

## Abbreviations

γ-GT, gamma-glutamyltransferase; ALP, alkaline phosphatase; ALT, alanine aminotransferase; AST, aspartate aminotransferase; cDC1, type 1 conventional dendritic cell; cDC2, type 2 conventional dendritic cell; DEG, differentially expressed genes; ECM, extracellular matrix; HSC, hepatic stellate cell; INR, international normalized ratio; log_2_FC, log_2_(foldchange); MASLD, metabolic dysfunction-associated steatotic liver disease; mig. cDC, migratory conventional dendritic cell; NAFLD, non-alcoholic fatty liver disease; PBC, primary biliary cholangitis; PC, principal component; PCA, principal component analysis; pDC, plasmacytoid dendritic cell; PSC, primary sclerosing cholangitis; RNA-Seq, RNA-sequencing.

## Financial support

The project was supported by the German Research Foundation (DFG, Deutsche Forschungsgemeinschaft (SFB841, KFO250, KFO306, No 290522633), ‘YAEL Foundation’, the ‘Helmut and Hannelore Greve Foundation’. AL was supported by the Clinician Scientist Program of the KFO306 and the Digital Clinician Scientist Program of the Berlin Institute of Health.

## Authors’ contributions

Substantial contribution to the conception and design: AL, CS. Data acquisition: AL, CS, ELL, SS. Bioinformatical analysis: AL, AML, CC, ELL, JX. Interpretation of data: AL, AG, ELL, LAL, CS. Drafting of the article: AL, CS. Critical revision: AML, AWL, CS, DS, ELL, MT, NH, SB.

## Data availability statement

The transcriptome data are available at the following link: https://zenodo.org/records/13990103.

## Conflicts of interest

Please refer to the accompanying ICMJE disclosure forms for further details.
