# Supplementary material for: Liver transcriptome analysis reveals PSC-attributed gene set associated with fibrosis progression
Source: JHEP Rep. 2024 Nov 12;7(3):101267. doi: 10.1016/j.jhepr.2024.101267 (PMC11848773; doi:10.1016/j.jhepr.2024.101267)
Supplement: Multimedia component 1 [file mmc1.pdf]

Liver transcriptome analysis reveals PSC-attributed gene set associated with fibrosis progression

Alena Laschtowitz, Eric L. Lindberg, Anna-Maria Liebhoff, Laura Anne Liebig, Christian Casar, Silja Steinmann, Adrien Guillot, Jun Xu, Dorothee Schwinge, Michael Trauner, Ansgar Wilhelm Lohse, Stefan Bonn, Norbert Hübner, Christoph Schramm

Table of contents

Supplementary Figures………………………………………………………………2

Supplementary Tables………………………………………………………………7

Supplementary Figures

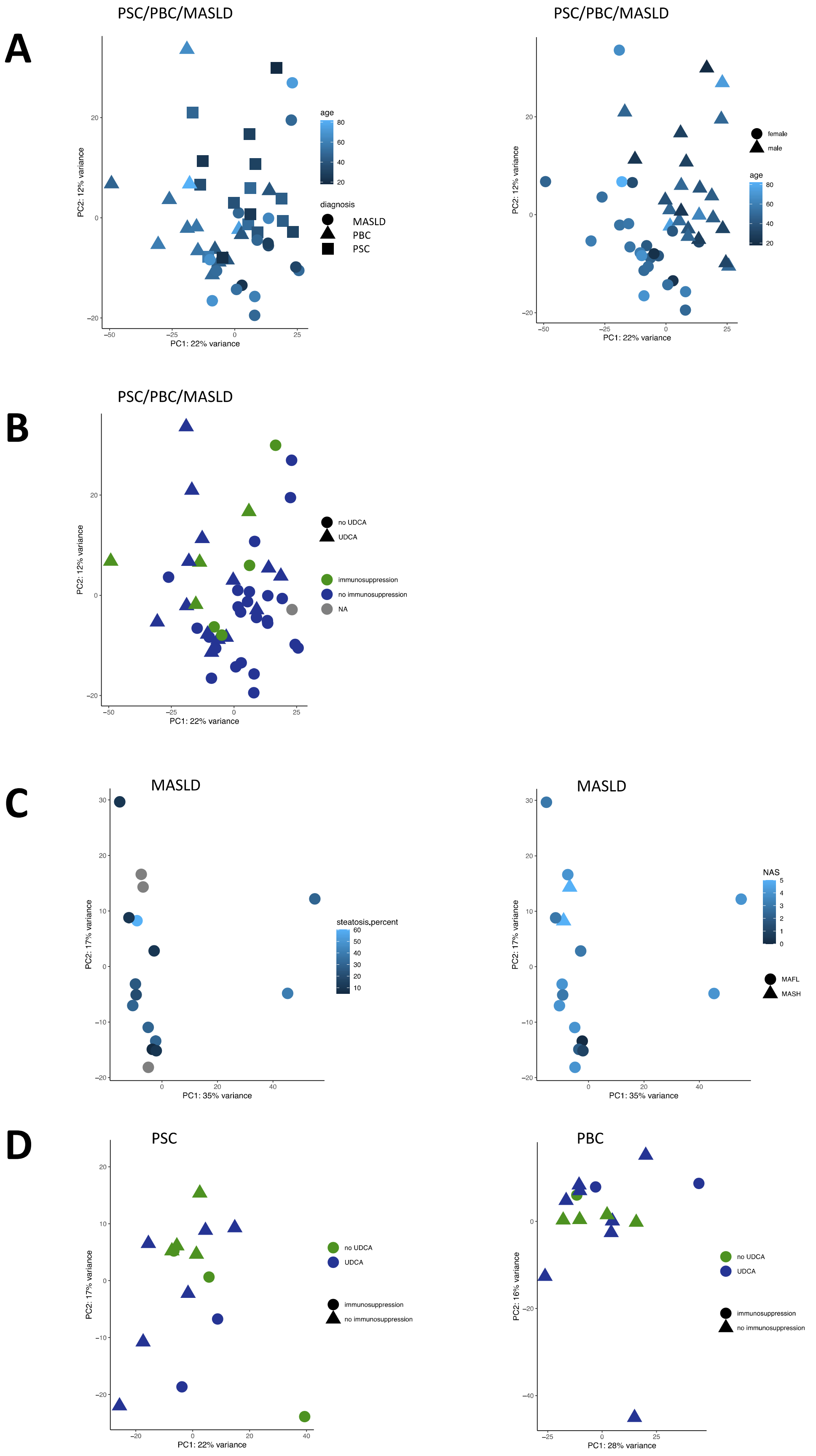

Fig. S1. Principal component analysis between samples of people with PSC, PBC and MASLD after RNA-sequencing analysis. (A) Principal component analysis of liver samples of people with PSC (n=16), PBC (n=15) and MASLD (n=16) regarding age or sex. (B) Principal component analysis after RNA-sequencing of liver samples of people with PSC (n=16), PBC (n=15) and MASLD (n=16) advanced or early fibrosis and immunosuppression or UDCA therapy at time of liver biopsy. (C) Principal component analysis of liver samples of people with MASLD regarding percentage of steatosis and NAFLD activity score. (D) Principal component analysis of liver samples of people with PSC or PBC regarding immunosuppression or UDCA therapy at time of liver biopsy. Counts of the filtered genes are used. MASLD, metabolic dysfunction-associated steatotic liver disease; PBC, primary biliary cholangitis; PC, principal component; PSC, primary sclerosing cholangitis.

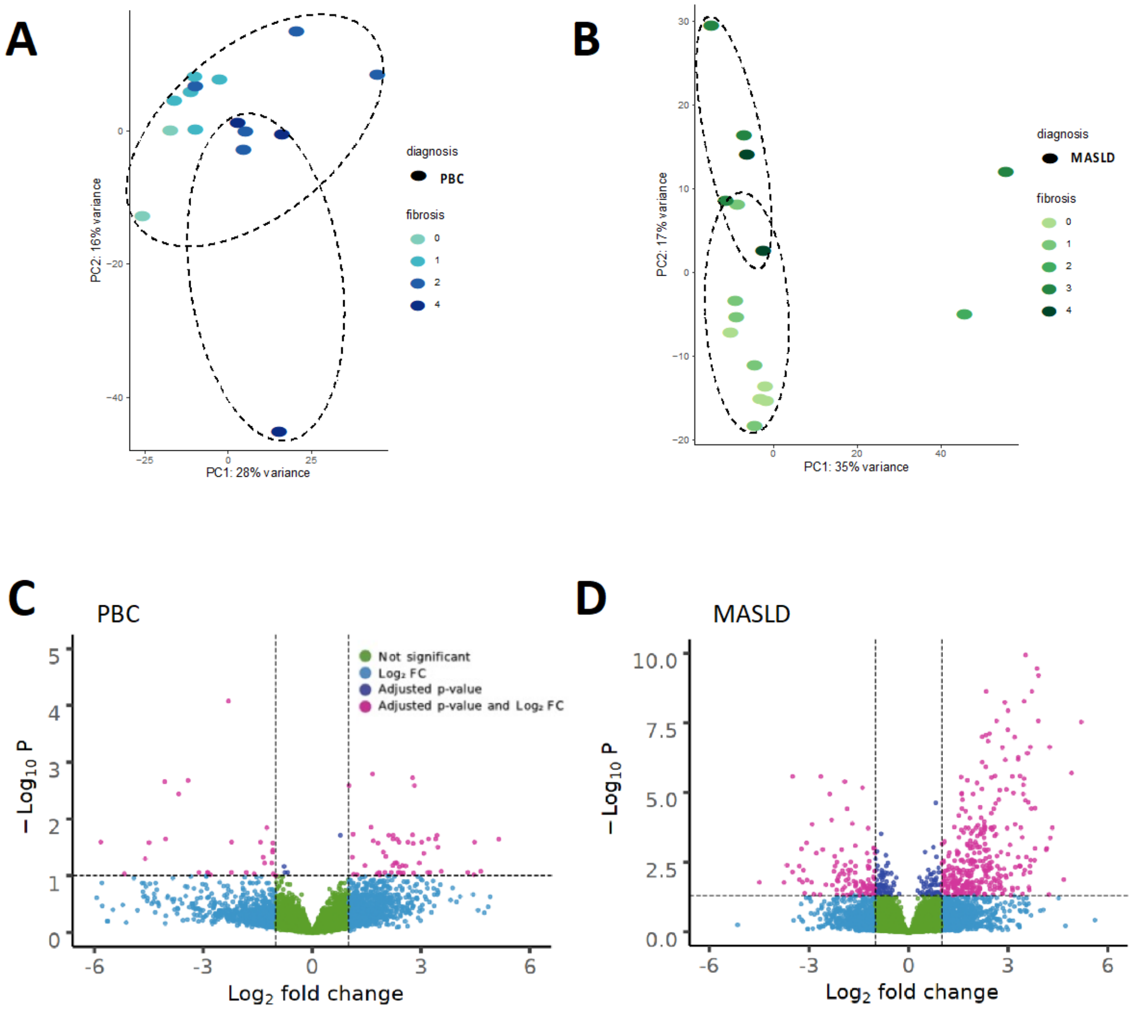

Fig. S2. Principal component analysis between samples of people with PBC and MASLD after RNA-sequencing analysis. (A)(B) Principal component analysis of liver samples of people with PBC (n=15) or MASLD (n=16) subdivided into advanced and early fibrosis. (C)(D) Volcano plot illustrating genes from the filtered data set, that show significant differential expression between advanced and early fibrosis in 15 PBC or 16 MASLD patients. Corrected counts of the filtered genes are used. MASLD, metabolic dysfunction-associated steatotic liver disease; PC, principal component; PBC, primary biliary cholangitis.

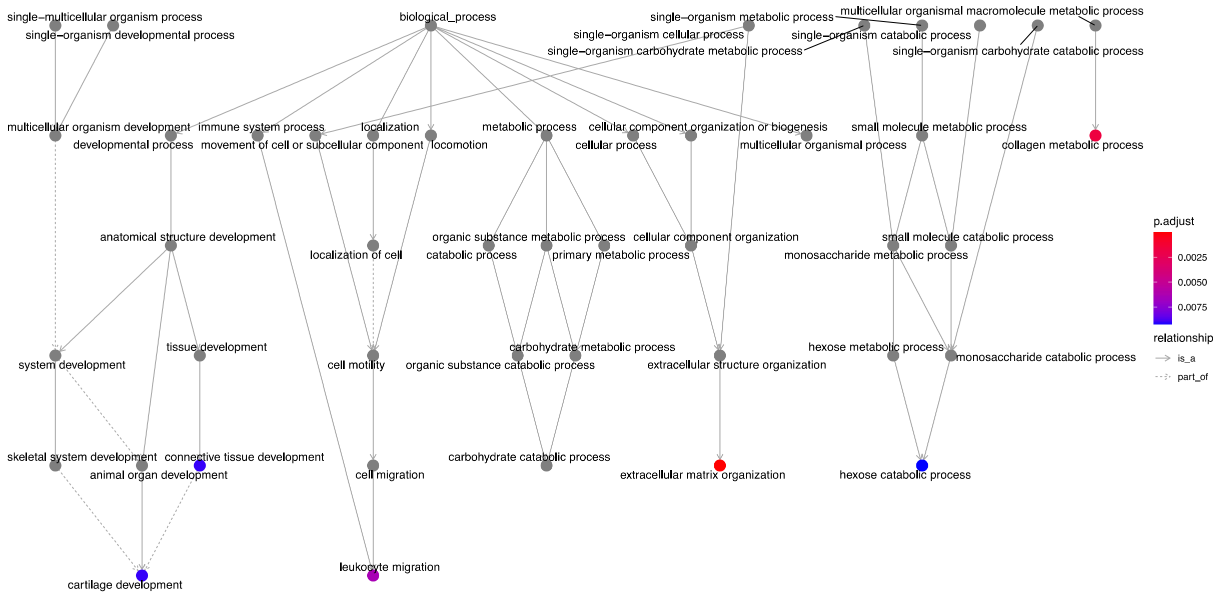

Fig. S3. Gene ontology term enrichment analysis as net plot for the biological processes of the 367 differentially expressed genes between advanced and early fibrosis stage in people with PSC. PSC, primary sclerosing cholangitis.

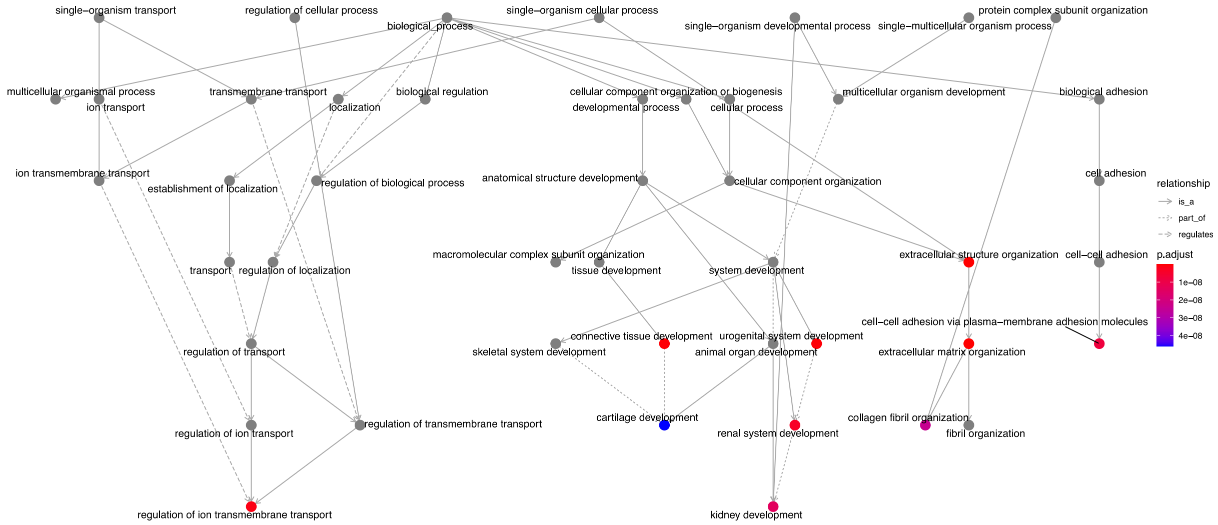

Fig. S4. Gene ontology term enrichment analysis as net plot for the biological processes of the 1351 differentially expressed genes between advanced and early fibrosis stage in people with PSC of the external cohort. PSC, primary sclerosing cholangitis.

	ID	Description	GeneRatio	BgRatio	pvalue	p.adjust	qvalue	geneID
**GO:0030198**	GO:0030198	extracellular matrix organization	24/134	368/18670	1.576242e-16	3.585950e-13	3.137551e-13	MMP2/COL22A1/COL9A2/COL1A1/FBLN1/SPP1/LAMC3/COL13A1/MMP24/COL4A2/LUM/MFAP4/FAP/TTR/CCDC80/COMP/AEBP1/MFAP2/COL16A1/COL4A1/COL5A1/COL8A2/COL1A2/FBLN5
**GO:0043062**	GO:0043062	extracellular structure organization	24/134	422/18670	3.411246e-15	3.880293e-12	3.395088e-12	MMP2/COL22A1/COL9A2/COL1A1/FBLN1/SPP1/LAMC3/COL13A1/MMP24/COL4A2/LUM/MFAP4/FAP/TTR/CCDC80/COMP/AEBP1/MFAP2/COL16A1/COL4A1/COL5A1/COL8A2/COL1A2/FBLN5
**GO:0001101**	GO:0001101	response to acid chemical	14/134	343/18670	1.794024e-07	1.360468e-04	1.190351e-04	MMP2/NFATC4/COL1A1/CCL19/BCHE/PTGES/FZD7/PTK7/OSR1/DGAT2/COL16A1/COL4A1/GLRB/COL1A2
**GO:0032963**	GO:0032963	collagen metabolic process	8/134	115/18670	1.772212e-06	1.007946e-03	8.819088e-04	MMP2/COL1A1/COL13A1/MMP24/MFAP4/FAP/COL5A1/COL1A2
**GO:0030199**	GO:0030199	collagen fibril organization	6/134	54/18670	2.378043e-06	1.031308e-03	9.023500e-04	COL1A1/LUM/COMP/AEBP1/COL5A1/COL1A2
**GO:0071229**	GO:0071229	cellular response to acid chemical	10/134	209/18670	2.719934e-06	1.031308e-03	9.023500e-04	MMP2/NFATC4/COL1A1/FZD7/PTK7/OSR1/DGAT2/COL16A1/COL4A1/COL1A2
**GO:0001503**	GO:0001503	ossification	13/134	398/18670	5.935879e-06	1.929161e-03	1.687932e-03	MMP2/PDLIM7/COL1A1/ROR2/SPP1/COL13A1/OSR1/MDK/COMP/GLI2/TMEM119/CTHRC1/COL1A2
**GO:0090596**	GO:0090596	sensory organ morphogenesis	10/134	256/18670	1.620625e-05	4.608653e-03	4.032372e-03	ROR2/FZD2/PTK7/EFEMP1/OSR1/MFAP2/GLI2/COL5A1/COL8A2/CTHRC1
**GO:0090103**	GO:0090103	cochlea morphogenesis	4/134	24/18670	2.411527e-05	6.095805e-03	5.333565e-03	FZD2/PTK7/GLI2/CTHRC1
**GO:0061448**	GO:0061448	connective tissue development	10/134	273/18670	2.812836e-05	6.399202e-03	5.599024e-03	COL1A1/ROR2/EFEMP1/LUM/OSR1/MDK/COMP/DGAT2/GLI2/COL5A1
**GO:0038063**	GO:0038063	collagen-activated tyrosine kinase receptor signaling pathway	3/134	10/18670	4.181388e-05	8.634641e-03	7.554936e-03	COL1A1/COL4A2/COL4A1
**GO:1903034**	GO:1903034	regulation of response to wounding	8/134	179/18670	4.554536e-05	8.634641e-03	7.554936e-03	PLAU/ANXA1/DUOX2/SPP1/FAP/PLAT/MDK/F3

Fig. S5. Gene ontology term enrichment analysis for the biological processes of the 150 differentially expressed genes between advanced and early fibrosis stage in people with PSC shared between people with PSC of our cohort and the external cohort. PSC, primary sclerosing cholangitis.

Supplementary Tables

Table S1. Differentially expressed genes after transcriptome analysis of liver biopsies from people with PSC with advanced versus early fibrosis. PSC, primary sclerosing cholangitis.

Gene	baseMean	log2FoldChange	padj
STPG1	66.5165213538814	1.03806382351779	0.0393619361208439
ITGA3	140.491643733775	1.29857840186187	0.0497488322542989
TMEM132A	58.8531565075408	2.04822978284	0.000241749779458621
MRC2	201.499198878481	1.15714907686606	0.0223032780992596
SYT7	2718.81624131427	-1.54886236133141	0.0152202092748826
PLAUR	38.5005642382046	1.26846629544632	0.0448373122614124
CLDN11	46.5668438654263	1.38483036426007	0.0244661722922571
CD6	55.9394183328679	1.10582450276233	0.0454614128411657
MTMR11	160.061699393749	2.37529140317237	0.000622502352025009
DPEP1	14.1874676617504	4.53579846520858	0.00458597922761639
IGF1	284.184593248002	-1.38758429731856	0.0402827850730024
VSIG2	88.9899453940755	2.4085011273085	4.30620795424635e-07
CD44	331.060984721737	1.36304811668883	0.00832512715352382
VCAN	111.491518490657	1.7665747549149	0.00850299609096921
USH2A	642.062708940655	-2.06940530406681	0.0116977238198055
ADAM28	42.8711096667586	1.58204477318708	0.0223032780992596
ARHGAP6	17.033732061928	1.40338184588059	0.0423505751194941
COL9A2	48.5289025979516	1.66437334892593	0.0128526411700704
LAMC3	144.745726033502	1.49783903480821	0.0323837981911038
FNIP2	1719.58088972418	-1.19018694637878	0.026534248976213
KCNQ1	53.5491142437102	1.35020119543227	0.0236623901567946
LAMC2	90.4082754003076	2.30673832792489	0.0233052590259399
YBX3	288.083341359193	1.05674858262818	0.026534248976213
ARSF	19.7069563651464	-2.78070088724248	0.0419993266919554
DGAT2	4579.81835160077	-1.20355082270454	0.0118928185999902
WISP2	30.7994260907607	2.29913315517945	0.0124161205408763
LPAR2	44.2142094058927	1.15420215291291	0.00655017618272789
OAT	1542.90593136032	-1.73903592573659	0.0276057703816685
CTNNA2	3.38951359877144	3.22412470987315	0.00396134687371227
PFKP	78.7534631163276	1.82304472366081	2.73654366503025e-05
PKM	693.447007056561	1.33941128472522	0.000470120997494607
DAPP1	25.7274906495985	1.28253769271701	0.0175657976800143
ASNS	77.5022963583056	1.41206549161739	0.0223032780992596
OSBPL3	60.9879587359552	1.295023216471	0.0366396814332282
LMCD1	117.828328303571	1.10178352942537	0.00207449662846941
GLI2	12.813974460162	1.6323629734446	0.0139433820757551
CA12	10.6051434031515	3.27174005204218	0.000281632096946323
RAP1GAP	496.725092147025	1.17529142663532	0.00170912887430793
FBLN1	206.813345227329	1.89761903682911	0.0124161205408763
FAP	14.0094081524046	2.77286165378191	0.00196670586334776
EDN1	57.0334020260579	1.33785526203861	8.73623709976284e-05
ITM2A	64.0061628743081	1.05390351386666	0.00136385399469593
LXN	46.618577607664	1.83555946991572	0.000700004968033066

MOXD1	45.0161043351725	2.41359941569679	5.55289415179156e-06
KCNN2	424.698370841547	-2.01954267953284	0.00563430533042114
DLG3	66.6095273696504	1.44468503699146	0.00456537744305191
COL16A1	132.807722815464	1.59894824176693	0.00386805578593389
IGSF9	485.407518999226	-1.65640195214071	0.0469638052227516
MMP2	310.375159692147	1.90588143224301	0.000872105410600536
FER1L4	16.9305112799082	1.44410382968871	0.0116556854823266
CPXM1	22.8505870528033	3.28378559007582	0.00114922710855477
TESC	61.8897802177956	1.69641918141901	0.0216007986056562
RGS1	19.3032177943152	1.43922389440659	0.0312217335423497
LYZ	933.023902413363	2.20065322302666	0.0127164446852048
NRCAM	30.0215567027906	2.85419294999241	0.00255275528777046
CCDC80	219.172506102333	2.04340547132688	0.000122016416905653
SMIM24	131.884272141246	3.34116497831848	0.0464727379851905
CRISP3	24.4015525322626	6.62424094672966	0.0359249832823872
TSPAN15	102.867531064846	1.23053762456644	0.00887682008157572
IGFALS	4997.54953887433	-1.89267883698521	2.49628243675649e-05
PIK3IP1	237.208003950012	1.03593157916488	7.66651259636765e-06
NFATC4	47.0681103360513	1.21615054928142	0.00232961698404084
EEF1A2	25.0220577236347	2.77692740926409	0.0453078967432116
FERMT1	9.88125984725863	1.48383889899852	0.0359249832823872
TLDC2	13.2741756492937	1.05369221904476	0.0462302862139009
NOL4	35.411650139348	-2.87020295233635	0.00124715670210108
PCSK1N	8.65655188989601	2.95834054224657	0.026534248976213
RGCC	26.9681730957459	1.92169546999318	0.014091852792574
TMC5	16.6940575233898	3.91808503345781	4.76206911894816e-05
IGDCC4	4.27321679994116	2.01206320183103	0.0129620692856621
PLAT	106.02582100555	1.24663586365919	0.0280786953008869
STMN2	14.0786133176183	3.40677652680793	0.000220054460930348
CLIP3	45.3149597818347	1.04465022155672	0.00155038885980495
ARRDC2	626.922737043663	1.41993997850275	0.00409024411769958
COMP	5.47313706560234	4.02993243642682	0.00239496580864581
SFRP4	15.3926277606175	2.4703086719809	0.000621270964562682
AEBP1	715.195852021125	1.53963858681242	0.00143355822134606
PTGDS	293.957087897795	2.05676291062277	0.000565400801631715
PPIF	3795.30589961834	-1.09503825028898	0.0216007986056562
GALK1	2836.11711193471	-1.17741419041636	0.0019225525183266
COL1A1	2361.88701912657	1.92255423403243	0.00569899792401553
MAP2K6	73.3327549659799	-1.39905889608863	0.044351658295654
PMP22	111.563527042939	1.2039800779637	0.014091852792574
CWH43	23.9304651301334	-1.56867433665581	0.04953582263368
GALNT7	29.8264411292846	1.11072391848587	0.0441055801676318
SOD3	91.3403125490206	1.46817637275783	0.044126307893406
GLRB	18.9041988420625	1.15458891506574	0.026534248976213
MDK	220.811932318709	1.66361320069601	0.00259562456054368
TBC1D30	18.0123796792122	2.37224404460533	0.0044913935293927
SLC16A10	293.289269506357	-1.40683231321362	0.0197471732766389
UNC93A	464.698004500009	-1.58929657515207	0.0129620692856621
MDFI	9.36919717807602	2.05644034080846	0.0245094851186537
PTK7	57.206783266752	1.09529549571709	0.0292106580113676
ENPP5	15.5638924274041	1.56372260485098	0.0411787160015384
C7	2603.77129834282	1.74983570549005	0.0018641315604665
GHR	5859.34470462983	-1.55106760768546	0.00514768431930594
DBN1	105.158815936901	1.65470903806304	0.00413244111925815

BCHE	3762.93786994956	-1.40382893150822	0.011117824620847
FGF12	13.1021743655572	1.41257542934114	0.0193437469589119
NEK11	30.9926466200761	1.1914757404014	0.000104146973540915
CISH	1050.25228895465	-2.56120238877464	0.0215700168340007
SLC4A3	12.9616067457824	1.8665617921188	0.0116977238198055
EFEMP1	266.577682527606	1.85814206512092	0.0116977238198055
CHST10	18.2051652868485	1.24475106758354	0.0428027953217387
QPCT	15.5066106597818	1.39381163842951	0.0134697203002485
PRG4	5065.54300009144	-1.05747138969852	0.0276257871124938
HAO2	7763.23881799858	-1.22691383617119	0.0161286483441998
RP11-268J15.5	13.9704884519003	1.40076617425078	0.0314440873350202
MFAP2	13.7747283707775	1.71972871655818	0.00625306630855344
RGS4	70.8948472067232	2.75547886933132	0.00559623275327865
F3	42.5794994811133	1.4094776218433	0.0253939773034529
A4GNT	9.01865779661433	3.91332881673388	0.00312573979164123
TREH	239.988855987171	-1.43553657919268	0.04953582263368
ZNF541	28.0093040514298	3.5392242888385	3.72456080498509e-05
TTR	177951.395480882	-1.23339379546909	0.0263661920557645
SPP1	1002.33839479807	2.29100358230446	0.0248623769337723
RARRES1	75.6928191718011	1.29701560483059	0.0419554492732191
CCND2	54.4660222420215	1.11515217484185	0.0167436813588506
LTBP2	162.24751375372	1.35234748023678	0.00443546492524697
DPPA4	20.964591204683	-1.94930101092816	0.00447317475911574
AKR1D1	7285.48703837096	-1.17883981481345	0.0250487557014472
PLAU	31.1890084134891	1.57831766407755	0.00665099636686889
IFT81	161.824156831574	1.01940290967059	0.0464727379851905
SSPN	18.7625624107862	1.6550446710053	0.0007133067010293
MMP19	48.1576951100384	1.52136834851111	0.0352941284316339
GPR83	5.29894658763602	1.83527490707152	0.0134697203002485
TTPAL	1559.93193303981	-1.04649863840155	0.0111238475280754
F13A1	37.3845216682584	1.69410906336621	0.0490500927582505
MMP24	26.6134893645728	1.30925215537635	0.0279165254472489
RASL11B	9.80531627061051	1.48980951893679	0.0303774718323401
POM121L9P	8.23075873377773	1.68883931165024	0.0366504396261426
KRT17	4.1879472265049	3.24078277070588	0.00255275528777046
SPECC1	32.2214990752708	1.1501215147502	0.000230976966558127
PDE11A	205.991407444703	-1.78091178710208	0.0213715871599184
ISLR	133.947539006317	1.28078816171815	0.0234331789451119
LOXL1	26.5865931190786	2.22536880442203	0.000872105410600536
BBOX1	1067.77498448175	-2.19570426853496	0.0143909455332959
LRRC6	23.8499809346267	1.01669676711197	0.00832512715352382
EGLN3	30.0410179970938	1.49085352273988	0.0279165254472489
PLVAP	205.760169453539	1.3568450342718	0.012682379528164
COL5A1	469.835515380134	1.41862474206778	0.0175657976800143
SLC6A8	31.0432112158602	1.31946115241595	0.00413244111925815
EDA2R	8.43876747674721	2.51641280405321	0.021399685810927
SLC6A6	75.870335964899	1.10090990800637	0.0448955648698778
PODNL1	4.98529719973018	1.79112892476592	0.043822453105594
SLC6A11	13.2339784647713	4.68529761462635	0.00011917986462994
JCHAIN	341.652956428773	2.00702300666729	0.0366396814332282
LOXL2	54.0912880337123	1.35308549582521	0.0176519096938576
HMGCS2	55408.2167670315	-1.06994114618196	0.0385384685924505
GRHL1	269.736226079776	-1.02397339875142	0.0390897962902708
RERG	31.6168831311096	1.22553763602753	0.0148363111542061

PIWIL4	36.5768311228057	1.86617330946368	0.00480963207387495
GGACT	187.151823243126	-1.02405763126228	0.0236623901567946
COL4A2	940.290427458998	1.26802054788983	0.00447317475911574
ANXA1	276.833916677907	1.18370856447084	0.0195864925309913
GOLM1	436.325474790801	1.49274826855451	0.012376804013246
TMEM243	44.7774246567758	1.01573874222658	0.00370180139910918
KRT87P	19.1549741317029	3.74677105597519	0.00447317475911574
KRT7	483.692143224011	2.14412865469576	0.00832512715352382
MICAL1	176.832679324196	1.05809325537701	0.0123507784191383
SLC9A5	3.9610819168653	1.79729794714746	0.0167070800306499
PCNX2	26.5065962182972	1.38679156923649	0.0113724977869291
VILL	48.2435682005507	1.07726714687503	0.014091852792574
THSD1	62.4214644431232	1.25151974113229	0.00814389363426249
NACAD	5.65326110754996	1.93153336368937	0.0138556565656711
CIB2	9.85011888977611	1.44640930610868	0.0417009252138584
ERMN	4.765738642636	1.66339992737847	0.0257592495901362
ALDOB	225490.608108383	-1.05672411873587	0.012378751560942
LRRC1	30.138605731697	2.08301397829693	1.21991855719815e-05
INHBE	2200.5448790829	-1.36604990600663	0.0490500927582505
LUM	645.922765518587	2.11377593436215	4.44549390857997e-05
FBLN5	366.176609579993	1.20372563360628	0.0257525918285535
DUOX2	137.737673843907	5.22551015302095	0.000233184730366442
FGF7	17.6938992615225	1.7743405054519	0.0022406734881307
MFGE8	229.912036723457	1.04461492188836	0.00708217259838561
GAS8	48.9946681653246	1.13118418863	0.0226308476254405
ZNF287	14.2040673739995	1.19255683046802	0.0225493448675838
ZMYND15	98.5300457083051	1.04740691943132	0.00832512715352382
SLC16A3	64.3129444700439	1.14641290162475	0.0267058371528344
FKBP10	170.002814715017	1.11472748150227	0.0118928185999902
VAV1	81.1352743029951	1.14851090642461	0.0439505299787088
COL6A2	2085.43151674754	1.05945370680476	0.0166337990848121
DNMT3L	33.011401530058	-2.43815502912347	0.0152982569172098
NTN5	7.8183396847862	1.65437060770287	0.0496868612625693
CTSK	159.40169667824	1.45056832776778	0.000123133277169609
PKLR	3256.35190480623	-1.25689609153314	0.028406839548112
OSR1	5.86629808638739	2.19393601765212	0.0152982569172098
BOC	15.3863556976769	2.04804968942634	0.000640579230835193
NCEH1	106.130482959207	1.01838463558949	0.0267058371528344
PCYOX1L	40.5801900271608	1.29993201537342	0.00447317475911574
N4BP3	9.06476519982771	1.53468244147493	0.0245094851186537
TPBG	29.9850078850926	1.32224909232844	0.00232335290843295
CXorf36	38.3443045272058	1.52340140640574	0.00358022542935344
GPC3	41.4989038436535	1.70598637832766	0.0262522606393077
CDKN2B	24.8376772001254	1.37140262073518	0.0368585835932663
PTGES	6.27469562221392	1.76046390273486	0.0352941284316339
SYT8	4.28282966245027	2.83128282943831	0.0135313281167945
ST14	587.960687558563	1.27593332239402	0.0285236322931399
TKFC	5366.13665613029	-1.08797939454806	0.0279165254472489
CNKSR2	44.2287517211804	-1.38363932505889	0.00458597922761639
LYPD1	2.20175723069016	2.75365073457383	0.0366396814332282
CCDC102B	25.9497295214434	1.05746723474594	0.0027253176849524
NOCT	100.082615238869	-1.367072513128	0.039851792529239
DPYSL4	5.26588812612898	3.42890834545507	0.00555094083037749
BICD1	22.5143423608878	1.1512958015596	0.0233052590259399

KCNE4	12.3272669303689	1.87696472279711	0.00307062914760934
DNAAF1	17.7360429672713	3.57742954004175	0.00315596331648237
ENPP3	133.006551044748	-1.06441017688131	0.0387908255640911
UCHL1	13.4192076518939	1.86758480351612	0.0403095994490255
CNTNAP3B	16.6091410821205	2.01122459618942	0.0129620692856621
TMEM55A	23.5645711951517	1.11050559845694	0.0195864925309913
TTN	6.38663401302457	1.89194552134127	0.0462253842821979
KDM8	1527.31960111126	-1.22871186808506	0.0267058371528344
FZD7	38.0236029373411	1.14178134209496	0.000591521357043573
ADCY8	4.69870414510546	2.78506807907168	0.0333653871285596
GLYATL2	3.92967031509806	4.24908328407629	0.0380156232830008
IL34	36.64854217118	1.31748393188525	0.00447317475911574
SLC30A2	6.60452970781876	3.39468760912824	1.4115576138242e-05
FANCC	1062.78660456142	-1.19360170618265	0.00114922710855477
MRAS	86.3560431101375	1.00543399356148	0.00529569835760759
FAM46B	8.86608717455505	1.84497313357988	0.0368585835932663
HK2	29.2218620661504	1.21601691197665	0.0352639480314662
ABR	215.541715129643	1.04950957723711	0.00344149508810554
UBASH3A	15.8059253623934	1.63552803295609	0.0389844835942515
GPSM1	105.351708456197	1.6475708989903	0.00686055532013383
PKN3	70.0367768352385	1.46772608201516	0.00413244111925815
BRSK1	5.14454628562609	1.55971030994676	0.0236623901567946
DMKN	47.864105182909	1.73271305385891	0.00194665288567357
ZG16B	28.0651841449293	2.57182186579996	0.0109943276702351
PDZK1IP1	149.960372848568	2.89120331579437	0.00012691068049179
NEXN	39.9467917674991	1.18943922214996	0.026534248976213
GBP2	552.661175782543	1.13537050405434	0.000871826265665489
FBXO41	29.2786595798882	1.08267717520427	0.0368585835932663
SLC16A14	57.30684770302	-1.69604872367469	0.0259968335312167
CFAP221	38.0658205658388	1.86782479813362	0.0213715871599184
MSX1	3.27343173969965	1.83757047446882	0.048291471857785
ELF3	378.618542241018	1.28381893412226	0.0214775095833773
CXCL1	43.0314288629911	2.61627544699973	0.00346378836813225
CPA3	22.3074716500728	1.56952413581876	0.0462308086170101
CDCP1	27.4316603665186	1.3701576968661	0.0352941284316339
MELTF	59.4807982425447	1.93449772091135	0.00239496580864581
MST1R	19.1880078607679	1.330345273414	0.0157651845071714
SPINK1	126.996618592167	4.76322621041791	0.00191116673853965
GPX8	34.8069291407154	1.30677455947349	0.0124161205408763
FOXQ1	18.3806187008834	1.71101329018328	0.00617008391111822
TMEM200A	7.76394771977101	2.10301759725424	0.0246358278611337
KIAA1324L	27.4609607204619	1.38082455109877	0.0221385087012464
COL1A2	1584.75397324539	1.65386485512243	0.0103344529882261
FNDC1	19.7483012589243	1.76284068389094	5.05822779988896e-05
CTHRC1	12.9179639498875	2.50391589210157	0.00132104546348734
INTS6L	58.6062911638115	1.08243753261654	0.00949989650352037
GPR176	45.6293251900392	1.29806257522318	0.0426249393078605
AMN	2353.31651169082	-1.71434931694145	0.0317486831648512
SPINT1	151.109810984131	1.74799346765198	0.00832512715352382
MFAP4	373.5431822839	1.37950067753002	0.0175657976800143
RRAD	32.5887544805902	1.62847152242031	0.0138556565656711
LDHC	12.6144920864277	1.79440553965628	0.026534248976213
MAP1A	11.5022877787756	1.58869927249401	0.0142626369324022
TTC16	4.57007182593728	1.59751961257696	0.0490423125533777

CERCAM	58.8769259762869	1.43512286801301	0.000978809314753162
PROCA1	20.7490923569601	1.0626840882965	0.00596399798120693
CACNB3	32.4711801056458	1.37982042352272	0.011117824620847
TUBA1A	323.053678525381	1.50309313950785	0.00156444875779015
TMC4	95.917559479241	2.17237155442249	0.0147784013974721
HID1	123.51895956888	1.10077416904571	0.000736517379479569
EVPL	7.0272274151282	2.06605666126713	0.0124161205408763
ABCA3	76.5568915121764	1.10199748562576	8.12005257606895e-08
VWCE	374.359918931648	-1.20414176470783	0.0307049369323104
MAP2K1	1231.39178268292	-1.10875569717594	0.0416571773360099
ROR2	8.44709734949091	1.96118407225615	0.026534248976213
COL22A1	7.98935112076064	2.92873039551097	0.000634718401513313
NRG4	27.7197494439662	-1.3057693403565	0.00261815967671356
ROBO1	103.165167259068	1.09514885431178	0.0345839313513714
ITGAM	84.5593193823196	1.21907486762045	0.0138556565656711
KRT86	10.5480684615895	3.18150050840981	0.000637186078544675
OSCAR	32.2365546907559	1.18665921215306	0.0490500927582505
PKIA	3.71229037322531	2.18776596055332	0.0351801870337779
ZNF439	23.5789460383552	1.0819369778326	0.0245094851186537
TMEM51	66.3334963328564	1.3098125020824	0.00617008391111822
COL8A2	19.3860381846532	1.48555894692282	0.011117824620847
ARNT2	69.9932217305949	1.37711856332682	0.0419848908500659
CCL19	131.817456666968	2.45637830314986	0.00850434798457618
MIR4435-2HG	45.8446638480062	1.49083961846192	0.00517958566696273
SULT1B1	463.54973421385	-1.49449197655218	0.0257592495901362
LRFN4	17.6767665302674	1.16862420605248	0.0158091077853298
MUC13	47.5057217788688	2.56761845646851	0.0440318479158445
GPR160	19.8392386650341	1.04720991189094	0.0393619361208439
CBX2	27.6182138301236	1.25393612462462	0.00832512715352382
MARCH3	19.8419977709723	1.28807683014489	0.0279469086210598
SLCO4C1	124.225326304498	-2.07747084364115	0.0318919830920021
SLC26A9	4.05257881428479	3.6380502652806	0.0174222085940857
MFSD4A	31.8269907829332	1.00612687798941	0.0336470517913728
SEZ6L2	54.2970279852555	2.46363608846924	0.00655017618272789
ASPHD1	13.275244652114	2.4233992277671	0.0351920346188881
ZG16	1525.38353122384	-1.33692878745728	0.0225493448675838
SLC22A1	24572.2468955797	-1.27835562637699	0.00653951956473021
CHST1	18.6128821541256	1.15073005108844	0.0245094851186537
SCUBE2	19.0899788725745	2.1364662921745	0.00617008391111822
MCTP1	33.4864843965165	1.07327825708491	0.00525535682332568
CLCF1	26.4288586119382	1.67104386855224	0.028386448030583
P2RY2	54.3184185810911	-1.30993261384497	0.0391541075129843
ETV4	38.8001697407004	1.96447811716801	0.000565400801631715
TUBB6	120.096168328372	1.06680821547597	0.026534248976213
JAKMIP2	62.6607816775768	-1.80569765638401	0.0257592495901362
SPHK1	53.5383521343724	1.41525754324914	0.0273912808807808
SLCO3A1	115.97625484726	1.10857999228953	0.00104025557066111
NCKAP5	105.881697215457	-1.19502722241914	0.0248896374757527
FUT2	10.4784795826099	2.53602998388858	0.014091852792574
ZDHHC13	40.016688075942	1.11305317841798	0.00832512715352382
MAMDC4	906.853315192298	-1.6823119416267	0.0339274385409484
CTXN1	3.94722254786508	2.68677642445604	0.0279165254472489
CA8	3.15255355192971	3.72472373916568	0.0325851172563534
MSC	24.1057362403481	1.51476318238163	0.0246358278611337

TMTC2	30.9656174341802	1.11235294584903	0.0393619361208439
B3GNT3	31.9215283640331	1.99026765298719	0.00686055532013383
FZD2	7.01321348520705	1.87065013065672	0.0329623078446972
BACE2	177.661615269918	1.39606898681741	0.0103479625143548
B4GALNT4	6.9841703370108	2.23592063081016	0.0263604600407009
SPACA6	12.1033208478301	1.31332386703112	0.0245094851186537
EPHB3	34.0385107082268	1.34280268182454	0.0497488322542989
ANXA2	2648.25880828215	1.2843018344508	0.0419848908500659
TMEM119	12.9777760642157	1.953616927646	0.0104071066557526
MEX3B	14.6837479329409	1.54385645837903	0.00194665288567357
PNMA3	21.6480975651754	1.16492097420367	0.0257592495901362
TACSTD2	147.706322342956	2.23732089041595	0.0236623901567946
PKP3	11.1336886150024	2.97117805865206	0.00655017618272789
PTP4A3	45.0097617522386	1.20690145086479	0.0111011461351316
ZNF93	9.6946617476483	1.61449473083614	0.0035883692724956
AHNAK2	19.6872328279866	1.18159873352536	0.0361018980635552
AGBL4	17.9279463194384	-2.19311324588356	0.0154973432397479
THBS2	221.656804958519	1.60125982627471	0.0138556565656711
DYNC2H1	58.5316728468527	1.25076765823113	0.0126567867962124
COL4A1	784.946739001199	1.13148336819659	0.0245094851186537
SAMD11	44.8336043778148	1.52481426425028	0.0114057928206436
IER5L	31.8348594385951	1.1768305920263	0.0490500927582505
C15orf52	178.395985215227	1.2863744379814	0.0373490476093378
BCL2L15	8.44444279113645	1.88138747727122	0.0477420191772099
SEMA4A	44.3688166546354	1.18566510241136	0.0095707105318286
MME	1262.26520403768	-2.05722836116042	0.00429997124754463
PDLIM7	147.063028902016	1.02539067813598	0.00443546492524697
ZNF763	25.7490621774644	1.3422029655819	0.0153852767581549
STMN3	52.1172710462064	1.20827346149613	0.000894519739610119
COL13A1	12.6653206123195	1.26212984191063	0.0497488322542989
ZNF677	20.4138086606504	1.13076848760694	0.0248890850462386
AKR1B10	400.634725848938	3.48831388059655	0.0266950007860527
ZNF607	32.472295758793	1.08006277479651	0.0104071066557526
CSF2RA	37.511121255304	1.88031821058377	0.00255275528777046
HLA-DRB5	939.974039884347	2.51436686212067	0.0221385087012464
STK39	38.7993350398025	1.45987802863234	0.00156444875779015
NEU4	1711.36286194206	-1.04814636459478	0.0273268100481388
HLA-DOA	159.768162036269	1.20151949472843	0.0428027953217387
PNMAL2	19.2384816106372	1.1527038108133	0.0298508690047339
TMEM231	54.2007906900034	1.05375752789696	0.0221385087012464
ADGRG1	90.8376098069352	1.38013166545624	0.00480963207387495
KRT81	7.01218251335316	4.19309646488982	0.000176191036074143
GYG2P1	25.4824257891795	-1.54455300946065	0.0373490476093378
IGLV6-57	46.9847950835676	1.74838601833091	0.0390713872916524
IGLV1-40	109.790807968524	2.13000776470995	0.0366504396261426
IGHA2	168.453029027125	1.8810679537933	0.0467701791836936
IGHV1-18	77.0921269189072	2.50198191067103	0.0193437469589119
IGHV3-33	34.1488167194649	2.04094070095642	0.0385384685924505
ACKR1	16.4494905504738	2.39929369816641	0.000468837537479265
SYCE3	6.62231851464434	1.89120165436044	0.0423885157123322
XKR9	63.221810046975	-1.75895370620339	0.0248623769337723
RP11-54O7.3	38.4670489958354	1.212333006838	0.00255275528777046
ASS1P2	139.356343063202	-3.25879891130639	0.000606008834802697
RP4-710M16.2	377.685975183447	-1.89093628564753	0.0408683638483783

LINC01057	11.9434237595697	1.67379454328478	0.000830722836932396
AC005077.7	15.8935025635437	-2.19936915210317	0.00569899792401553
CTAGE4	6.9845402671504	1.97493840867418	0.0312233595258778
AC091729.8	18.1904075427724	-5.40074854243956	0.00555094083037749
RP11-397G17.1	161.265839569125	-1.75001707657808	0.000550392833555507
LTB	81.201449302388	1.2641119588369	0.0365566623073179
RP11-492E3.2	3.31567310007607	3.88820587690576	0.0207421187354651
MELTF-AS1	6.29607869034823	1.64723833923156	0.0428027953217387
AC013275.2	8.6347456113208	1.94374345377122	0.0463753909679883
LINC01160	5.83124726497043	1.46787437896554	0.0373661105988245
IGHV4-31	25.9127037962404	2.22854671282826	0.0385384685924505
AP006285.7	91.1463785551054	-1.77061389543315	0.0311356973335343
AC004862.6	274.955228135826	-1.18983523889114	0.0462253842821979
LINC00665	23.7424398004553	1.16035252983897	0.0393619361208439
LINC00342	96.7345620904456	1.45854522017873	0.0126567867962124
C12orf75	46.3501322002751	1.55678234353631	0.00368141964900117
AP006216.5	61.2987085992623	-1.41880803273268	0.0334309846472487
LINC01554	1469.62489894127	-2.90967576052876	0.000634718401513313
RP11-753B14.1	15.3823216491403	-3.81492718476513	8.49527863011772e-05
LINC01625	46.6499227186037	-1.95537515048314	0.0437807765068879
IGKV1-6	30.6727222466806	2.338417725677	0.0466454391619011
IGKV1-16	36.6019505238674	2.18361380286525	0.0351801870337779
HLA-DOB	13.5432673919713	1.75422374281124	0.0248623769337723
TDGF1	18.0333906516952	1.3637355891971	0.0236623901567946
FLJ22763	227.926272494158	-1.1745914621208	0.00832512715352382
IGKV3-11	257.252602830223	2.26611379680037	0.0236623901567946
IGKV1-9	81.1075716190529	2.27743980366678	0.0437484215050008
MBL1P	144.033776301417	1.16315731735514	0.0408683638483783
PALM2	35.8453041514964	-1.46243020511586	0.0309368216268838
DRAIC	126.5794226653	-1.6461141172713	0.0066822537620625
RP11-422N16.3	107.865065453742	-2.93641016038175	0.0215700168340007
TNXA	29.8064392060392	1.97603793667219	0.0257592495901362
RP11-434D9.1	371.982439134753	-2.1671590269302	0.0428501587391136
RP11-685F15.1	13.922378964398	-2.19114118651442	0.0276257871124938
RP11-397E7.4	4.03977592303764	1.70605807556213	0.0490500927582505
CTC-558O2.1	28.6611331246595	-2.3652456074241	0.0279165254472489
RP13-870H17.3	9.53157620191968	2.82773369414234	0.00919786347198352
RP11-535A19.2	7.45630230310244	-2.62373823583742	0.0262522606393077
RP11-44N21.1	5.6153617003144	2.0098625949151	0.0022406734881307
RP3-416H24.1	12.0632345368413	2.4618790041799	0.0161286483441998
CTC-378H22.2	2.35088565420691	2.32285651085474	0.0425472231211591
RP11-753A21.1	8.45782266226598	-2.02501087008574	0.0490500927582505
RP11-1069G10.2	6.40563668438189	2.77851325882879	0.049458085377701
RP11-800A3.4	67.9821817508672	-1.29992644941092	0.0490500927582505
TMEM178B	4.52277493902139	1.89701343950311	0.0345839313513714
CTD-2529O21.2	23.2053857386813	-1.72344695027542	0.0462302862139009
LA16c-325D7.2	2.98095252463193	2.52308268494715	0.011117824620847
LRRC37A7P	82.9656953766242	-1.41903091311697	0.0248623769337723
CTD-2132N18.2	19.5783247846369	1.02576715436324	0.0257525918285535
SMUG1P1	130.068198153431	-1.45979904920527	0.0271607660138017
MIR24-2	25.6306952125367	1.01896209306957	0.0447251356454561
MILR1	24.8544695455967	1.33518109256162	0.0225148566806415
CD24	665.40107612331	1.75887878828265	0.011117824620847
XXbac-BPGBPG55C20.1	4.16929469281986	2.54941331228089	0.0245094851186537

ADIRF-AS1	13.175817208874	2.28107770934571	0.0167436813588506
RP11-631N16.4	14.8057888706164	1.10975409793905	0.0438528821398412
SGK223	71.7149796552796	1.38321183999589	0.014091852792574
CCL18	41.4764287511523	2.57443683194739	0.0128526411700704
CNTNAP3P2	4.10394818822346	2.25175055382107	0.00617008391111822
RAB7B	22.6023000456575	1.7029270336662	0.0007133067010293
AC233755.2	13.463726285018	5.24570880996466	0.0175657976800143
RP11-39H3.2	82.8210483406775	-1.80336243027007	0.0082079776748903
RP11-329N15.3	13.9882548990521	1.31502956739264	0.0439505299787088
RP11-40A7.2	58.1593611699522	-1.33818350704963	0.0462253842821979

Table S2. Differentially expressed genes after transcriptome analysis of liver biopsies from people with PBC with advanced versus early fibrosis. PBC, primary biliary cholangitis.

Gene	baseMean	log2FoldChange	padj
SEMA3B	106.252673745257	1.01792631305576	0.00299320894590054
ARG2	32.0729270466563	3.20777520349771	0.0222148662092035
SLC22A17	13.7403940201114	1.68833664617511	0.0234164759451462
B3GAT1	330.785632228951	-4.06290649959507	0.0021060449852488
VIPR1	575.717882931384	-1.08356171629421	0.0389246839940229
FBXO2	845.560539466882	2.03335159840117	0.0464395152480275
SLITRK3	141.796227599134	-7.54955745924069	0.020980880193268
LRRC61	432.346917701521	1.12733983229235	0.0197207464992929
GDF15	196.926422673467	2.7718079466055	0.0197207464992929
RAMP1	2407.52480009748	2.39915590002154	0.0263117180533465
SPG20	299.961313984638	-1.0868660759023	0.0418273408769321
GSTM1	1582.75926057157	9.87650162240374	5.50522552777621e-63
SLC44A3	268.581554387947	1.95878251671691	0.0389246839940229
FAHD2B	93.4973695611101	1.81902185279525	0.0272233175025397
ALDH1L1	18991.0908116763	-1.24856661210005	0.0163438287425528
KAAG1	3.9127703136235	4.47240667553635	0.0234164759451462
POMZP3	57.3373983706961	2.11335071041715	0.020980880193268
ST14	537.75367632175	1.66834506720528	0.00164111199165595
ADGRA3	2911.92614174655	-1.4201106423066	0.0268043877341672
SLC5A11	22.4309546667405	2.33753037361579	0.0222148662092035
DMKN	75.3862580409731	2.2323493083895	0.020980880193268
COX7A1	163.159863296235	3.40787881125903	0.0222148662092035
SPINK1	482.533821635518	5.14793339101465	0.023214474538679
CMYA5	272.527747876613	-1.08305755179008	0.0291244846392159
LARP6	88.7326086555368	2.22629880634019	0.0222148662092035
FABP4	60.7504922995816	2.82329635806906	0.00280602875809337
HSD17B13	9158.00730163744	-2.30217962782736	9.10407076759242e-05
TMC7	15.1572375186213	1.62354104838993	0.0144297275592029
UGT2A1	85.8256205688496	-4.03488888125007	0.0222148662092035
MUC13	73.6972753508615	3.46238039206834	0.0298076303153813
SLC6A19	35.1362617579552	-5.82473646587567	0.0234164759451462
ASPHD1	25.9078522249864	3.44364123907278	0.0202044571838998
SCUBE2	33.1664438125838	2.64246704344229	0.0234164759451462
FAM9B	96.3013702358622	-3.67503944350075	0.00346566614437743
PCDH9	63.0913127052523	-2.22133963532328	0.0234164759451462
CIDEC	31.8597573790422	3.02180518001379	0.0234164759451462
DKFZp779M0652	153.39051284766	2.78732121501196	0.0272233175025397
HRAT92	10.6580372010041	2.77106236476161	0.00164111199165595
CES1P1	435.508701204926	-4.49100042465018	0.0268043877341672
CYP2B7P	1288.72065215491	-3.41646189956983	0.00204099697165222
CTD-2325P2.4	26.0616631966413	3.07767230723641	0.0389246839940229

Table S3. Differentially expressed genes after transcriptome analysis of liver biopsies from people with MASLD with advanced versus early fibrosis. MASLD, metabolic dysfunction-associated steatotic liver disease.

Gene	baseMean	log2FoldChange	padj
CFTR	273.956395227161	2.94378755232819	7.72809766076411e-06
DBNDD1	119.513371036829	1.39286767360068	0.002730647434486
CD38	44.6779935254418	-1.08190358047328	0.026588573140817
PRSS22	18.917215775178	2.91495841633327	6.76233427497206e-07
ITGA3	110.705723528929	1.56911638433046	0.00750288892446449
TMEM132A	29.5774447647859	1.97638240705748	0.00281405758548834
TNFRSF12A	128.597475726922	1.80452711873048	0.0098742846633194
USH1C	28.8605399812367	2.47616347759235	0.000190430625450584
PROM1	90.9543797449458	2.09447965369921	0.000147723553602656
SEMA3G	72.5929765591515	1.61336344825135	0.0233231940731534
EHD3	392.515678810604	-1.38956761307103	6.66860366658118e-06
MTMR11	89.7292961554225	1.36138756357488	0.0188508661606287
IL20RA	14.271065138535	1.74693439392454	0.0264252006666996
VSIG2	67.5656688691542	2.39115248663795	1.45553036620174e-07
SYT13	24.3774284668038	3.16271905903779	1.02685554441465e-05
PLEKHB1	40.7556629833221	1.91941844785035	0.000691768336020804
GRAMD1B	99.6374210556334	2.27221871854293	0.000416531132867255
ARNTL2	65.578509903385	1.32677204922387	0.017582772565213
SH3YL1	318.301668078193	1.02123995151687	0.00720883168484891
DEPDC1B	14.6887984754936	1.75537635227251	0.0250617882312635
CASR	3.83873371630235	2.72795124018841	0.0117542840360053
VCAN	93.678704067049	1.58860626496817	0.0459168154350462
FOXC1	8.30892123154557	1.51176842617186	0.00757856413671407
MCOLN3	11.9356672802958	1.87865193232783	0.0250617882312635
LAMC2	75.1860785012479	3.30091109498489	6.48338826169106e-07
SLC12A2	201.348120348318	2.0496162392261	0.00075136325920484
EYA2	24.232463636465	1.52834439324295	0.0323289214077222
FGFR2	1322.5864068305	1.07365289298412	0.0138366825828019
PFKP	46.4929768369819	1.48330087290251	0.0459132693690945
RASGRP2	195.997908845571	-1.10459096357444	0.0457779717529985
KCNAB2	374.749245988332	1.83401448905355	0.0103850146392027
LMCD1	57.5281559492406	1.06148042783556	0.015889114721459
EVC	202.971290819002	1.49963875638329	0.0428164876502561
IGF2BP2	58.8373777475369	1.66125400438036	0.000274068718191157
CA12	81.4990740812502	3.81113397274314	0.00100743593241414
SLC12A1	9.4912022475983	-3.22992001131789	0.00106400310315629
MCAM	154.746133108518	1.66492951675722	0.0030844140288367
CAPN6	25.7245070976755	2.57623607046331	0.00017245513278913
LXN	26.7829499672959	1.11555364360328	0.0414718509567788
MOXD1	27.0426238732717	2.29546909975761	0.00640417735103604
GRHL2	21.251223493569	2.44045950032557	0.00422497003299876
TTC39A	19.5587648951474	1.36291656855599	0.0243355551232263
PTHLH	3.5346941669612	3.1258279738989	0.0259270057926525
TESC	39.6753972156991	2.17614137794829	0.000301168748959087
SLC26A3	51.5146861925741	2.579470773864	0.0312975677988687
SEL1L3	285.367397479363	1.069664324964	0.0098742846633194

SLC7A8	113.545082513508	-1.2140976528748	0.0300113422931293
TGFB2	19.2197916359792	2.40617214093687	0.0051539398427581
BAMBI	195.099554174906	1.219152262725	0.00580256742107098
GADD45B	883.992078465281	-1.36767781131579	0.0170304921618695
SLC5A1	96.9461678655945	1.93868631018605	0.00582804038374654
RAB36	12.2627302990534	1.46629492859641	0.00150439781754773
PVALB	20.0338925820189	-1.99510061462099	0.00632935922343394
UPK3A	5.16842232869413	3.21380966844653	0.00481231192534056
IL2RB	172.322765245668	-1.1828522473503	0.00481231192534056
TRIM9	70.1889140952072	2.75953935084994	0.0278807281060706
FERMT1	14.4480663857714	1.56734824702434	0.0133283150988447
JAG1	256.558871065499	1.41641594110788	0.0113578674616
LAMA1	20.0926689637424	2.05814630734097	0.00453914872692047
KLF5	31.6555217512223	1.79442568152322	0.00932816435671946
RGCC	19.394646523958	1.77351057137454	0.00180575762272094
RHOV	3.78402127399666	4.14316463478596	0.00112877693793146
NIPAL2	298.41918299032	1.14233970579664	0.0428164876502561
ESRP1	22.3138756594283	2.96947259158155	0.000190430625450584
TUBB4A	22.3471926678759	3.36526064621246	0.00432955555923546
CLEC4M	848.920161722724	-1.48537480963343	0.0405601786791036
APLP1	5.30449306729883	2.28338671429657	0.0439165864549174
ITGB8	29.7700622769209	2.23144971649875	0.00432955555923546
WNT2	32.8636427310125	-1.74505643771252	0.0030844140288367
NPTX2	9.62965827166842	2.28099968479639	0.00010972240660449
GLIS3	117.447804577222	1.07937567093929	0.000576738884052627
LGI1	87.0148182808396	-2.21086876236801	0.00432955555923546
KRT23	31.9040646868593	6.44433455423202	1.055566537679e-10
CCL2	74.4731058458238	1.46705869777161	0.0184083632545901
RND2	154.11597205453	-1.79962691426358	0.00825076375194256
SULT1E1	867.301872810941	-1.0540593673883	0.0437897070425335
MS4A6A	872.201217197026	-1.00135325909988	0.0249987913529739
VWF	422.228116416635	2.22083996694831	8.11948860429638e-07
SYT10	5.66402832519141	-2.21992135086148	0.00191202906015148
AKAP3	35.4861500843647	-1.20430449235509	0.00542262729805956
RASAL1	6.75767456731976	2.43171967225187	0.0034596658758818
PERP	2554.8063773124	1.05161591303283	0.0030844140288367
ARFGEF3	18.8381666362298	1.56001324870948	0.0378120764537426
MDFI	5.26386280500746	1.44161062449007	0.0389309643321594
ENPP5	21.2379518790502	2.15876516546056	3.34407532579434e-05
CDH6	79.899737775005	3.45653499062753	5.27280140732182e-06
DBN1	69.648953329083	1.45233978277631	0.00123909456152555
LRRC31	128.229369121918	-1.03730551318859	0.0318563223610417
C3orf52	8.78598729592234	2.39190407024375	0.0110404412403313
ITGB6	10.7080373287067	2.43771762613553	7.68602748320055e-08
CLIP4	76.6578809992056	1.99662687990729	0.00293915918529691
GNLY	102.544384008063	-1.05924611792409	0.0412761019867418
PLEK	116.136734869643	-1.16081068634345	0.0232382282203324
RGS2	72.4924460510633	-1.07436032435649	0.0233004564834127
RGS4	35.6422141792003	2.74452005358152	0.00559261786876192
MUC5B	121.482888122141	4.91199231756705	1.98653836294689e-06
C1orf198	236.399794466022	1.23061931064125	0.00144116367888336
EPCAM	118.791236917673	3.47993702169548	5.3146446381688e-09
SFRP5	231.396053413547	2.13014363720182	0.00144116367888336
MTHFD1L	71.6191728277431	1.1563052848102	0.0152767163956546

TNFSF11	16.0290576897159	1.34021412218905	0.0250617882312635
EGR1	348.845021504932	1.94303803670496	0.0287505088820584
DUSP4	20.8092476773847	1.44601396829712	0.0158752642106027
DPPA4	14.4878664199243	-1.5841549977971	0.0182154902826885
SLITRK3	50.0610564224719	-3.66332427171644	0.00404843348648364
AKR1D1	7057.38610879044	-1.02373102886345	0.00100743593241414
BICC1	151.874414635224	2.64867522694715	2.67049408802529e-08
BHLHE41	18.5064626878415	1.93853404042077	0.00281405758548834
ITIH5	91.0881114902835	1.74182149210598	0.0162000350184996
IL13RA2	75.9542591173228	-1.43460604623915	0.0158448998322581
ZNF831	15.0628688553483	-1.21091283977868	0.0133377594372195
PMEPA1	84.8715388888103	1.97846650749119	0.0014766262011178
POF1B	17.9500187664721	2.62381931700134	2.46105204118366e-05
SOX4	113.832893209089	1.85286904938975	0.000848963706513276
CXCL6	59.2735048812414	3.45276205035066	1.11974582565716e-05
MYRF	683.291918751718	1.15829751481604	0.00609207158992444
ABCC4	106.149430303538	1.56605321973715	0.013535186573776
SOX9	135.149058128648	2.32872835102171	1.19297217759789e-06
SDCBP2	203.929238348985	1.93316640470917	0.000238869507890472
FFAR2	4.69822643394887	-2.12760032256322	0.0155604187503747
GLIS2	63.1104088371323	1.36623302146514	0.0212727837334848
AIF1L	71.2808595263687	1.85896426949124	0.00100743593241414
ATP13A4	23.9289231988848	2.44836333190039	0.00551329508444726
LIF	19.6913229071714	1.86496384042434	0.0430410608322617
APOBEC3A	6.67870316332191	-1.9971835100411	0.0314190177282974
PODXL	233.385484654723	1.35088852162055	0.00819399936198138
LOXL1	23.5522050897924	1.83300850915256	0.040800537219139
AP1M2	32.064819679853	3.35647611177966	2.54464653987023e-06
FOXJ1	2.56224443458611	2.82348415577464	0.0428164876502561
ACE2	162.280233974437	1.45537048489443	0.0051539398427581
PLVAP	163.764499325707	2.21459143075861	7.41509620540167e-06
STK33	7.91193433609077	2.06384322229834	0.0381104889837708
SLC6A8	39.2830186191414	1.78057868283452	0.000783727542836514
EDA2R	23.066458815281	2.78332053763417	0.00135459449026624
GALNT15	43.9800761115508	1.93796984905335	0.000936734324309568
G6PC	8331.58384402622	-1.17231821836811	0.0462692498716562
MAP1B	50.7246600916965	1.52073453672494	0.000407252060612292
LGALS3	323.535743021319	1.76119897370354	0.0249987913529739
RHBG	408.547771348027	-2.07101733575314	0.00842206070849562
RAB25	22.5173011650079	2.75087461292229	8.16306946934542e-06
KANK4	132.008977788563	-1.88582063112856	0.0141336435239488
CHI3L1	1631.63453347042	3.07808802625336	0.0084129154964436
POSTN	140.664715968913	-2.01695451690739	0.0011205224833021
BEX2	19.7480716721464	2.14228548875203	0.000891470775464358
FAM83F	115.707901400542	2.0865989685762	0.0337359259141619
PTPN22	19.5874322623027	-1.02860052624984	0.0404584384867764
VTCN1	63.993997322709	3.48043422226593	3.17597506228603e-06
EMP1	97.5976191544653	1.5055872219038	0.0178756357498322
LRP4	22.1394151296128	-1.85160117301385	3.85980426232745e-05
DTNA	132.976673710269	2.49827263916898	0.0030844140288367
CLDN10	86.9139645291705	2.28659936683511	0.0011205224833021
GOLM1	489.864080635488	1.51614381243484	6.55469323607001e-05
TES	112.054611666529	1.01443777201495	0.0321883402287623
AGAP2	19.1325296773105	-1.00767862790203	0.0297604124593965

KRT87P	3.74875939323019	3.1414813253456	0.0320262237216149
KRT7	270.88212646969	2.99020820614743	5.56346254377866e-08
KCNH3	13.852629585378	1.58770528790664	0.000218073354201783
PCNX2	24.872139551175	1.3397509274904	0.0138366825828019
NACAD	4.40608032949385	1.85830164050558	0.0199122952052687
LRRC1	27.0382304190859	2.21934810666234	1.0106569813408e-07
FGFBP2	9.55120805198759	-1.5867194857175	0.0167334898999925
MMP7	70.0100115135461	3.52231707278672	1.92137708021331e-05
FXYD2	21.7111312625585	1.83579873545454	0.00641610437425617
PAQR5	40.8567718366731	2.08552677223154	0.000912493021418272
SLC44A5	39.327734400206	2.56472258156113	0.0119371554539066
LOXL4	134.598358403672	2.72015184218308	1.79742007358844e-05
CH25H	6.36648183199183	2.12660756847687	0.0167322315388678
ANXA3	49.7288641894889	2.14587333480769	0.00276953180104953
LGR5	68.7272578279518	-1.74394707009969	0.00609207158992444
PDX1	12.0279485436482	3.86751776909744	3.55139650692923e-10
ITGB7	53.4310461856919	-1.05296012230236	0.0227178205471219
CDH24	18.4180989007269	1.17271860759729	0.0314190177282974
RDH12	25.3928606747335	1.99273552281282	0.0408480755356006
TPM1	895.649057181195	1.01785343055298	0.000691768336020804
CHST4	130.911601053616	2.29633514123295	0.000211770285476572
PLPP2	56.2381531469487	2.60512626475309	2.52509702421378e-06
SIK1	18.3492810123153	-4.49023014576764	0.0167334898999925
LMTK3	6.56762863833589	2.04669652195219	0.0428164876502561
CNKSR1	48.7240097738705	2.34251202323256	2.36410071749572e-09
IGSF3	62.3709334748936	1.64158676030097	0.0285791890560433
KCNN3	17.4941326480817	1.76785538321193	0.00905971810371764
ILDR1	14.8090898970221	1.92013956603932	0.00300971518681784
SCD5	62.7381746684434	1.87939865796819	0.000288656335607061
NKD2	30.0181598757526	2.4054535025151	0.00609119130522683
N4BP3	6.83742489468198	1.94447568974889	0.0167250815282735
TENM2	172.346977467918	-1.92520613298517	4.06594109645072e-06
DCDC2	178.540123027816	2.99732904676201	1.15664899931939e-08
GPC3	187.845873905032	4.24839973023085	2.32472366575292e-07
RSPO2	6.30199785141297	-1.94121019790573	0.00839750595380541
CDKN2B	18.748704011878	1.76951132445228	0.0202114433626093
CDKN2A	12.4389842351509	3.19062814335691	1.03664770992397e-07
SLC25A25	1113.28442596917	-1.05623276044506	0.000967190608896738
ANKRD1	6.91907612355167	4.26400718527473	0.000420993901394291
TENM4	26.6798411046478	3.42640779259204	0.000407252060612292
ST14	324.474229540643	1.56498841090422	0.00265740477102986
KLRF1	20.7606531767704	-1.69234532252702	0.000131288052812213
ADRA2A	16.7870487365538	2.51446377363624	0.000211770285476572
HSPB8	54.6610528179813	2.30223575057668	0.000174169071927295
CAMK4	50.199407830084	-1.22485055129566	0.00432955555923546
KCNJ16	36.5701082397949	2.15502867946187	0.0281124596557948
HS3ST3A1	11.4315765792697	-1.80807674332036	0.0121861301065523
CABYR	6.25893577066717	1.72671515751886	0.00798254909073145
CHST9	359.699021885274	1.27602793419875	0.0188244561715107
THY1	64.0329134505905	1.79518353977381	0.00275108668890254
TNIK	94.7636547232015	1.510592899715	0.006018021923541
NCAM2	360.857929481584	-2.58945921303705	0.00111669869872824
SLFN13	90.21186014795	1.38124396073847	0.0459653960939531
APCDD1	69.9463432531852	2.13012614361304	0.0246009943736072

HKDC1	292.14117362158	4.16197299114733	0.00100743593241414
B3GNT7	24.5791982591513	1.44156926071535	0.0450505085663366
SLC34A2	45.5886127435572	2.39534156686705	0.00112674115958887
RHPN1	56.1315712662335	1.0996639749254	0.00649244532455086
NPM2	42.5191063223894	1.88672333971341	0.00300971518681784
STC1	12.9452309027455	2.21465423292732	0.0128761393458746
CLIC6	80.8222935983723	3.90987114619447	2.67049408802529e-08
TUBBP5	13.2258630278127	2.20224202906223	0.0167334898999925
TPPP3	32.3407057824836	1.71568097645499	0.000187416073431572
ZNF233	10.2120985099774	1.19430467923913	0.0264543226196962
TMPRSS3	34.9604367391879	1.80738020841205	0.000190228330270655
FCRL3	19.2352562407679	-1.31697051836582	0.0233830015628059
CYP3A7	1687.06824133455	4.07416709544267	1.51759952480241e-32
DMKN	45.1454260417267	1.38800520970579	0.0143841850225964
BICDL2	16.1211538139145	2.24208822007416	0.00288617702502002
PAQR4	10.5116671263937	1.37167654299249	0.0254323530355539
PDZK1IP1	100.767995718006	3.91570490975977	6.23666247764746e-10
RBP7	34.7734111811087	1.62378617427301	0.0252148934991824
VANGL2	15.8587279757996	1.33225408051525	0.0481400419624993
FCGR3B	18.6224483860033	-2.4896765703469	0.0201972235737074
FCMR	41.6634951230528	-1.02753217955464	0.0463397316186124
CAPN2	1159.40868899413	1.71500240084142	4.58705048914487e-05
CFAP221	27.3517664090116	1.48111859118732	0.0356961035817483
SGPP2	12.8051337465279	1.94068873885236	0.00382917857248763
MSX1	8.03299023606069	2.43371122780529	0.0218743063404119
C1orf106	14.3441766098807	2.66247085943241	0.0018773796283953
SLC22A15	30.2961938430104	2.64084572656995	0.000311169642624131
CXCR1	7.80377041773006	-3.49596632130543	2.6497441837039e-06
ADORA1	12.9473425682977	1.87289051759139	0.00978559145720976
CD200R1	27.1231476923504	-1.04910479288536	0.0304342953126775
CDS1	22.9423461899464	1.76321812683843	0.0064876260201619
CXCL5	4.12581002323635	2.93878230695996	0.0113578674616
LIPH	11.4325020054119	2.16168799641677	0.000312011722617842
MELTF	39.3117902603292	1.30899968038144	0.00979579818716153
HSPA4L	239.260588399495	1.18079636218087	0.040482681771472
NDST3	11.9743196503147	-1.25607472377574	0.0367200838917854
ELOVL7	28.2397278705085	2.27861704386459	0.000153734707388776
F2RL1	114.616890707237	1.34596439689038	0.00971051447101889
CDC20B	2.08855721947496	3.62190147705162	0.0250617882312635
ANKRD55	38.3185192298889	-1.01829828239268	0.00655654407497125
NEUROD6	5.76957360109236	-1.92931027733888	0.0246247824486426
ADCY1	3015.11518592518	-1.24937382839212	0.00150103734898597
DIRAS2	9.66394433734045	2.33034621304709	0.00802104581970176
TRPV6	36.7677254484637	2.04797603612547	0.00458501429160891
WNK2	93.2700973360636	2.30175844992689	0.000702561967553672
SLC18A2	10.5146201613699	-1.47729846326369	0.0323289214077222
FRMD7	16.0336764949393	-1.74559047257965	0.0475722529832353
TC2N	89.8695406563552	1.64762842469497	0.0213939185481315
SERPINA12	23.1660323330146	1.84524564657229	8.28471150222208e-05
SPINT1	121.127635381052	2.90398684274081	5.77250993253188e-09
AVPR1A	536.249032927358	-1.96917620110797	0.0493936369290227
LARP6	62.1548850668966	1.49957348792564	0.00144116367888336
SERPINB8	140.474028744239	1.19902918388396	0.0018773796283953
PRKCB	66.9864578537001	-1.17526829030645	0.0283736619858364

ACSM1	235.361774083964	3.2048299867035	0.000137129437320616
LDHC	15.029599246359	-3.05885784014164	0.0138682000737234
PRR15L	48.154181446739	1.60601993535184	3.77668358978971e-05
GPRC5B	174.818796233463	1.55249660828712	0.00632622442174225
CACNB3	27.0333548203108	1.11524990843021	0.0188244561715107
TMC4	123.541552540364	2.5918816276229	0.0229569191686067
SPINT2	170.498992900721	1.77267233803538	0.000691768336020804
TMIGD2	5.38495834275817	-2.15768660317907	0.0475761980232135
TRPV3	32.6310196377814	1.97598748591612	0.0233830015628059
GGT6	17.4939085516002	2.20686550484609	0.0167322315388678
KRT80	15.384203396269	3.58008823867684	3.85697240142595e-07
FILIP1L	61.9034657661441	1.47345822166823	0.00170083927469687
BDKRB2	16.171139355695	3.12875306226143	2.6497441837039e-06
NPNT	97.6422249647255	1.57467149919878	0.0404470943770781
MFSD7	193.28413591076	1.42140564033137	0.0182154902826885
CXCL8	33.0676667263316	3.30111566716251	0.0020881563310238
GPR183	19.967155050269	-1.127088561633	0.0290199700510326
CTNND2	32.7371784232079	1.91369620924636	0.0391110189590975
ZPLD1	7.82418282294768	2.54510857355321	0.0462434834791338
SERPINA9	5.74663507075758	1.83210339657191	0.0182154902826885
SEMA3E	12.8670242666986	2.43663143088534	0.00203141951634407
PDGFD	92.7339696655831	1.87273745499261	0.00265740477102986
CDC42BPG	53.1662001412751	1.02087584282704	0.0224359652488166
KCNK3	13.3715007294084	-2.31833642506816	9.57712459232721e-05
KRT19	109.045910593279	1.58421559766842	0.0351337049417762
NMRAL2P	2.70931245300819	3.6623347093524	0.00736520068953118
LGALS4	3556.2926685393	2.17874460431091	0.000205442545474132
ID4	24.1829972612084	1.73015057197744	0.018240899465426
CXCR6	31.2924570759311	-1.21905010256794	0.000190430625450584
ZMAT3	476.470358546244	1.14234186647571	0.0409103150052263
OVOL1	5.65677090275559	3.4614871625576	0.0084129154964436
EVC2	20.5517799798371	1.35531735315639	0.006018021923541
TNFRSF10C	18.6584576458778	1.66909882387277	0.0227821604592961
TRAPPC3L	2.91905846450316	-3.19360151891037	0.0166374089363057
MUC13	12.0947001828063	2.77382774920228	0.0167250815282735
C11orf80	46.4087241834896	1.25556350326975	0.00905460395833625
HAP1	12.7256675651183	-1.81803568362595	0.00606154277413209
CBX2	22.722512519823	1.37987100762411	0.000174169071927295
CD34	132.378307047735	1.60219518796443	1.0605677257676e-05
PHLDA3	134.74177183553	1.50561449347081	0.0145961075081613
SLCO2A1	103.483987282796	1.62151298846683	1.79742007358844e-05
SEZ6L2	34.9154688833304	3.52885689033166	1.16531647905751e-10
ASPHD1	10.5474657601908	2.32203825073915	0.00234382487208137
CADM2	10.6827479168621	-1.58177526796777	0.0204391548681969
CLCF1	14.1517832571113	1.58011838166206	2.79700656622325e-06
ETV4	23.4101644136363	2.29364451333381	0.000190228330270655
A2M	48555.8090720097	1.9003837716149	0.00770605137475211
SPHK1	38.9172931345356	1.94709110922283	1.11974582565716e-05
GCNT4	49.4413365352155	1.65600739041472	0.0059483407919081
ERICH5	275.843746259717	1.50957669534836	0.0175160008109977
TMEM125	18.8962546651319	2.25222080533715	0.0002823200517001
B3GNT3	55.970185181497	3.30305338943673	5.36890763592517e-07
TMEM150B	42.3443945519295	2.26293104088348	0.00559261786876192
PRR26	53.7413706091044	1.04748102334193	0.0027402060544844

PRF1	88.9427984973331	-1.18652866316514	0.00288617702502002
S1PR5	15.2347315014194	-1.61427003972749	0.0076289543246958
NQO1	60.1297461266686	2.56394450545357	3.85980426232745e-05
MAB21L2	23.5731920079204	1.75510893242981	0.017802909543641
C6orf223	2.89652328758261	3.55186143638918	0.00205530857572877
TIGIT	18.8911747231124	-1.23967061681955	0.0224359652488166
CLDN7	312.410297825051	1.17662616164442	0.0246009943736072
LDOC1	30.6133631534872	1.07907537637686	0.0217021873995268
BACE2	130.87372784978	1.59856084824656	1.11974582565716e-05
ANXA2	1718.03887058873	1.85883657816179	0.000537628569965476
C1orf116	43.267498887363	2.49441079487337	5.62107719892863e-05
MACC1	14.3500554937651	2.34762622588452	0.00404796741325496
B3GALT5	33.9486118248473	2.34261267189738	0.0064876260201619
FAM3B	28.2182777449149	2.33362545091002	0.00125745304025186
LRRC55	60.4566843511476	-2.7749400390224	0.00149264398537461
PRKX	92.1871314894785	1.05315493866298	0.00909347498297562
ADRA2C	7.37860606512618	1.76281866114793	0.0249987913529739
TACSTD2	74.5266675697977	3.30971218054287	9.70745076520113e-12
PKP3	17.0378387368375	3.60361391976998	2.21112630486719e-05
MUC6	159.84410536703	2.49893176126082	2.88399462836292e-06
MUC1	27.8329079077513	2.68390964362566	0.00828343598520972
MYBL1	39.8138660941157	-1.00636718936458	0.00149264398537461
CTD-2600O9.1	8.14457480120482	1.67428796517939	0.0212076146064624
KCNJ11	10.2959937901769	1.34015032841868	0.0167334898999925
FAM69C	4.82687956031115	-2.3019934358275	0.0212076146064624
ZCCHC16	31.8475984406533	-1.37807955102661	0.0206225480614195
C6orf132	19.1972718355066	1.93313098048023	0.0250764420402524
SELL	58.4368311351513	-1.06292594381106	0.0108683883805256
COL25A1	34.2846060661438	-1.15730334535238	0.0184083632545901
CLDN4	60.2023378256625	2.18010920694708	0.000268498183231101
FAM150B	20.2410742213558	1.76581007630888	0.0208332181088841
KIF19	37.2853722661503	-1.05723008190392	0.0287505088820584
SFTA2	4.45000020250681	3.63785612125317	0.0316325373474369
SRC	237.625644588053	1.08800841668622	0.0101082821407059
ADGRA1	15.1987896096287	-2.64061456327214	2.6497441837039e-06
VEPH1	25.989057974463	2.49915761464664	0.000185125625702886
PDGFA	84.800506404564	1.29204252825083	0.0167632508683847
SLC28A3	22.0722405513318	1.96141532062135	0.00276953180104953
CR1L	6.55086869666096	-1.79226326011592	0.0190362712954183
PTMAP2	13.1029701767519	-2.85910494939105	0.0450505085663366
S100A6	403.414875283625	1.41194532345975	0.0339022265046587
AKR1B10	1259.98574684702	5.19860322624065	2.91673528742692e-08
SULT1C4	33.8329564736917	2.0112785608967	0.000591476557368028
SULT1C2	177.108925726975	3.35170775720995	0.00432955555923546
PLXNB3	28.3704412270167	1.60091340796152	0.0327146077307638
RP11-163F15.1	8.07521369206726	3.81471782633397	3.63732358748245e-05
FOXO6	5.74120921067184	2.73818966118391	0.0212076146064624
GPRIN2	10.9348156483118	2.14881629240991	0.0290199700510326
COL15A1	28.6861775048506	3.05484687338102	0.000756548420221007
LAYN	13.4514474390093	1.85963880751812	0.00369386031568281
NCR3	9.16821248844234	-1.92041164219053	0.00400064912913566
PSORS1C3	12.6269420396467	1.90233966436206	0.0167250815282735
NAT8B	46.1141534087114	2.03260846330064	0.000186476713306085
ADGRG1	112.157227794878	1.77292227261987	3.61018822115176e-06

SLCO1B7	8.67631183693816	-1.53381352331243	0.0331525124947241
CYS1	55.8181024087922	1.32172238534021	0.00495154037194302
RP11-473M20.5	3.58770366181201	2.59994532440938	0.0140793972501447
DOK6	56.1494538677904	-2.37355378379383	1.11974582565716e-05
TRDC	17.5251828287832	-1.59685442993819	0.00432955555923546
HNRNPA3P6	15.1006535142831	1.00720406282526	0.00349596603688623
RPS7P14	7.07998042845581	-1.85194221126916	0.0481400419624993
AC098614.2	11.8669792464292	1.91029113753828	0.00256475499622181
TAX1BP3	11.8320635818049	1.83616052388111	0.0327708975963136
UCA1	16.6652460933323	2.99388627456121	0.00165388237508148
COL28A1	34.6390368382426	-1.60717558925102	0.000808378223708392
ATAD3C	172.458405161439	-1.70253522237402	0.0385328725554505
RP11-465B22.3	22.6123950530654	3.69556425649595	3.77668358978971e-05
TDRD15	33.6961628093778	1.44673191272292	0.0285791890560433
FAM19A5	43.7306361698797	3.67829182873725	0.00263337601118412
PPP1R3G	48.9118083964407	-1.42956858662328	0.0279156838638082
FXYD7	4.87246383437442	-2.12226904443334	0.0110220395089333
CYTOR	7.13472465304243	1.79127090562487	0.015624415409214
RP3-395M20.2	9.63681729951276	2.8312600061987	0.0428164876502561
AP001626.1	5.9055442967529	2.07095200539748	0.0249987913529739
SATB2-AS1	2.70279616550456	2.6822714598245	0.0259181332209531
NPY6R	62.7950543932824	-3.0723702374125	0.000635672940337574
CROCC2	34.1065949521483	-1.03542523231329	0.0123126598225338
RP11-289F5.1	8.43185359276993	-3.7543611674887	0.0169049801711688
HORMAD2-AS1	30.879660059516	-1.36475699275344	0.0198047036434074
MEOX2-AS1	3.07047508629508	-3.13042588125311	0.0405773474729345
RP11-367G18.1	7.24313527747639	1.41332562036293	0.0250617882312635
AC013275.2	6.35471976689444	2.33686097242912	0.0162575867271289
RP1-27K12.2	56.8492464561322	1.61208936017501	0.00258745032730043
PIK3CD-AS2	4.23399912480022	2.04465915964753	0.0459653960939531
HS1BP3-IT1	50.4141543644466	1.04634992795912	0.0155604187503747
AC016831.7	12.3549282983167	1.93617401986097	0.0386148265187823
RP11-255H23.2	10.3438471006139	2.18671597113784	8.14014681362477e-05
DPP10-AS1	5.11469487906772	3.43629889340516	0.0435900571581891
RP3-417L20.4	2.60434976388868	4.23038127041578	0.0459168154350462
C12orf75	33.9893638435778	2.06350745615055	4.54149597540626e-06
AL162759.1	4.59553724813656	2.87779485398159	0.00552885059502126
RP11-439H9.1	7.45826313449111	-3.28141296730994	0.0133648855951827
RP11-59D5__B.2	4.11714814729297	2.05337422796687	0.0144880932042998
ARHGEF38	6.26795281210967	1.67362235002981	0.0402168114494961
AC005550.3	12.6240140881412	-2.92203699953352	0.0167250815282735
RP11-278H7.3	8.71105015724727	-3.49797923307226	0.00713468839547332
RP3-395M20.8	167.140503284087	1.21993036301373	0.00432955555923546
GATS	46.2690252758026	1.20805237983573	0.0120627087443952
AQP1	796.381774002583	2.33473668057738	8.66563444501805e-08
LILRA6	19.0250332883082	-1.40786667208577	0.0407834791376932
PTCHD4	7.60007152854065	3.67108086646054	2.32472366575292e-07
LINC00861	25.2206991170647	-1.25915018276663	0.026588573140817
LINC01550	10.3431603417752	2.78973783138949	0.0239890221581195
CCAT1	12.3640086223785	2.79086761008397	0.0098742846633194
PCDHA11	3.86616280720519	2.32329375844963	0.00993464473283081
LINC01021	29.7842232330075	2.6549919015747	0.0155604187503747
CTD-2005D20.1	18.0863834368643	2.9256231790172	0.00955536283373315
TRNP1	64.0446362541457	2.59858846962653	0.000363395389786314

KB-1615E4.2	5.91710384093901	2.29777145974479	0.0449202886206357
RP11-150O12.3	5.55089371667177	3.28723661097312	0.0263235010898177
RP11-626H12.1	17.2954094276102	-1.32287275314516	0.026588573140817
RP11-575F12.2	9.92044743895397	-1.28840410030493	0.0413499757674839
ADGRA1-AS1	3.64681103159751	-2.66868187135587	0.0463397316186124
KRT17P8	283.029798097511	-2.24840181290957	0.0067505709028866
MGAM	6.83173272606938	-2.17128905839766	0.0316718817400346
RP11-44N21.1	11.0637347206739	2.45457384002552	0.0290199700510326
RP3-416H24.1	6.25102075264105	2.49508819308651	0.0051539398427581
RP11-25E2.1	11.5525351146803	-1.65654523446589	0.0151790249257851
RP11-510M2.5	13.9847633414542	1.73697925814988	0.0405601786791036
RP1-239B22.5	8.10061130045011	1.48161625278702	0.0405601786791036
RP11-114G11.5	48.2674484582793	-2.90241815377775	0.000137129437320616
RP4-555D20.2	5.17574382424085	3.07884932140233	0.0486110083705687
RP11-353N14.4	3.234701729639	2.89889638163957	0.0314190177282974
RP11-353N14.5	29.4973608766557	4.33251295004476	0.000185125625702886
GJA5	32.1052898721038	1.61307557630264	0.00955536283373315
RP11-484N16.1	9.81002433055178	2.49311361597493	0.0170955675492222
CTD-2008P7.9	15.5819786552878	3.38185038104782	0.000184363280707364
LINC01482	34.6978146815048	-2.15334573074272	0.0140234132088284
AC004156.3	12.0839761356464	-1.47296177883282	0.049929857576501
RP11-120K24.3	19.7029990728073	1.19311206254778	0.0232382282203324
RP11-465B22.8	10.9562518491914	2.07468322911156	0.000367648192977179
CD24	523.70812706959	2.82404623715818	2.3804977305159e-07
RP11-284F21.10	8.33405605911352	3.49751856032156	0.00609207158992444
RP11-468N14.13	7.61182933692702	3.66133562765461	0.00432955555923546
RP11-54O7.18	4.20933702665961	4.67196700717462	0.0132050038443973
RP4-568C11.4	163.831473389198	1.34935625860033	0.0249987913529739
PI4KAP1	131.400760016476	1.05455885019877	0.0345933136021904
RP11-569G13.3	6.91090464227913	2.67183775868181	0.00432955555923546
RP11-96A15.1	7.41326736538114	2.11176220131674	0.0387120965413674
HIST1H3H	24.7190464961488	-2.16267480258624	0.00312763116945927
RP11-114G11.4	6.11421554138446	-3.2863478096481	0.00432955555923546
RP11-437J2.3	5.81399535845764	-3.16291986782852	0.00609207158992444
CYP3A7-CYP3A51P	38.8467796908418	3.71399674806295	2.36410071749572e-09
BISPR	90.3193289382196	-1.60461584490272	0.0231528039663219
RP11-403F21.4	11.5519788923604	3.33357695850581	0.00428056428386065
CTB-76P12.1	11.8732265299321	3.79749841839338	0.00265740477102986

Table S4. Baseline Demographics and Clinical Characteristics of the external Study Population. All data are presented as mean (SD) or n (%). Serum bile acids, C4, and FGF19 data are available for n = 40 subjects.

*UDCA and UDCA conjugates were removed from the BA pool.

Abbreviations: PIII-NP, type III procollagen peptide; TIMP-1, tissue inhibitor of metalloproteinase 1.

**Characteristic**	**Total (n = 74)**
Demographics	
Age, years	46.3 (11.3)
Female	22 (30)
Body mass index, kg/m^2	26.9 (4.8)
UDCA use	50 (68)
Ulcerative colitis	37 (50)
Ethnicity	
European descent	60 (81)
African-American	12 (16)
Asian	2 (3)
Liver tests	
Alanine aminotransferase, U/L	85.1 (72.7)
Alkaline phosphatase, U/L	332.6 (302.4)
Gamma-glutamyltransferase, U/L	408.6 (457.4)
Bilirubin, mg/dL	1.1 (1.3)
Albumin, g/dL	4.0 (0.4)
International normalized ratio	1.0 (0.2)
Platelets, ×10^3/uL	260.5 (98.0)
Other biomarkers	
ELF	9.47 (1.35)
Hyaluronic acid, ng/mL	100.8 (188.2)
TIMP-1, ng/mL	331.3 (163.7)
PIII-NP, ng/mL	9.6 (5.7)
Hemoglobin, g/dL	13.9 (1.7)
Total bile acids, pg/mL	1590.2 (586.2, 3649.2)*
C4, ng/mL	8.6 (4.3, 24.6)
FGF19, pg/mL	135.8 (60.4, 221.4)
IL-8, pg/mL	19.5 (12.0, 41.0)
Liver histology	
Ishak fibrosis stage	
F0-F1	26 (35)

**Characteristic**	**Total (n = 74)**
F2-F4	37 (50)
F5-F6	11 (15)
Hepatic collagen content, %	5.3 (5.1)
α-SMA expression, %	5.3 (7.7)
