# Supplementary material for: Liver transcriptome analysis reveals PSC-attributed gene set associated with fibrosis progression
Source: JHEP Rep. 2024 Nov 12;7(3):101267. doi: 10.1016/j.jhepr.2024.101267 (PMC11848773; doi:10.1016/j.jhepr.2024.101267)
Supplement: Multimedia component 2 [file mmc2.docx]

**Journal of Hepatology**

**CTAT methods**

Tables for a “Complete, Transparent, Accurate and Timely account” (CTAT) are now mandatory for all revised submissions. The aim is to enhance the reproducibility of methods.

- Only include the parts relevant to your study
- Refer to the CTAT in the main text as ‘Supplementary CTAT Table’
- Do not add subheadings
- Add as many rows as needed to include all information
- Only include one item per row

**If the CTAT form is not relevant to your study, please outline the reasons why:**

|  |
| --- |

- 1. **Antibodies**

| **Name** | **Citation** | **Supplier** | **Cat no.** | **Clone no.** |
| --- | --- | --- | --- | --- |
|  |  |  |  |  |

- 1. **Cell lines**

| **Name** | **Citation** | **Supplier** | **Cat no.** | **Passage no.** | **Authentication test method** |
| --- | --- | --- | --- | --- | --- |
|  |  |  |  |  |  |

- 1. **Organisms**

| **Name** | **Citation** | **Supplier** | **Strain** | **Sex** | **Age** | **Overall n number** |
| --- | --- | --- | --- | --- | --- | --- |
|  |  |  |  |  |  |  |

- 1. **Sequence based reagents**

| **Name** | **Sequence** | **Supplier** |
| --- | --- | --- |
| NucleoSpinKit |  | Macherey-Nagel, Düren |
| High capacity cDNA Reverse Transcriptase Kit |  | applied biosystems, by Thermo Fisher Scientific |
| Taqman probes |  | applied biosystems, by Thermo Fisher Scientific |
| TruSeq stranded mRNA library Preparation Kit |  | Illumina |
|  |  |  |
|  |  |  |
|  |  |  |

- 1. **Biological samples**

| **Description** | **Source** | **Identifier** |
| --- | --- | --- |
| Liver samples, needle biopsies, taken between 2011-2018 | University Medical Center Hamburg-Eppendorf, Germany | **N/A** |

- 1. **Deposited data**

| **Name of repository** | **Identifier** | **Link** |
| --- | --- | --- |
| **In progress…** |  |  |

- 1. **Software**

| **Software name** | **Manufacturer** | **Version** |
| --- | --- | --- |
| R |  | 3.6.3 |
| ggplot2 | https://github.com/tidyverse/ggplot2 | 3.4.2 |
| DESeq2 | https://bioconductor.org/packages/release/bioc/html/DESeq2.html | 1.26.0 |
| Clusterprofiler | https://guangchuangyu.github.io/software/clusterProfiler/ | 3.14.3 |
| EnhancedVolcano | https://github.com/kevinblighe/EnhancedVolcano | 1.4.0 |
| Python |  | 3.8.3 |
| Scanpy | https://doi.org/10.1186/s13059-017-1382-0 | 1.5.1 |
| Anndata | https://doi.org/10.1101/2021.12.16.473007 | 0.7.4 |
| Pandas | https://doi.org/10.5281/zenodo.3509134 | 1.0.5 |
| Numpy | https://doi.org/10.1038/s41586-020-2649-2 | 1.18.5 |
| Seaborn | https://doi.org/10.21105/joss.03021 | 0.10.1 |

- 1. **Other (e.g. drugs, proteins, vectors etc.)**

| **qPCR** | **Manufacturer** | **Identifier** |
| --- | --- | --- |
| AEBP1 | Thermofisher Scientific | Hs00371239_m1 |
| CCL19 | Thermofisher Scientific | Hs00171149_m1 |
| CXCL1 | Thermofisher Scientific | Hs00236937_m1 |
| FBLN1 | Thermofisher Scientific | Hs00972609_m1 |
| MFAP4 | Thermofisher Scientific | Hs00412974_m1 |
| NFATC4 | Thermofisher Scientific | Hs00190037_m1 |
| SFRP4 | Thermofisher Scientific | Hs00180066_m1 |

- 1. **Please provide the details of the corresponding methods author for the manuscript:**

| Alena Laschtowitz, MD, Charité Berlin, mail: alena.laschtowitz@charite.de |
| --- |

**2.0 Please confirm for randomised controlled trials all versions of the clinical protocol are included in the submission. These will be published online as supplementary information.**

|  |
| --- |
