# Supplementary material for: Liver transcriptome analysis reveals PSC-attributed gene set associated with fibrosis progression
Source: JHEP Rep. 2024 Nov 12;7(3):101267. doi: 10.1016/j.jhepr.2024.101267 (PMC11848773; doi:10.1016/j.jhepr.2024.101267)
Supplement: Multimedia component 4 [file mmc4.pdf]

Liver transcriptome analysis reveals PSC-attributed gene set associated with fibrosis progression

Alena Laschtowitz[1,2,3,4,5,*], Eric L. Lindberg[3,6,7], Anna-Maria Liebhoff[8], Laura Anne Liebig[1,3], Christian Casar[1,5,9], Silja Steinmann[1,5], Adrien Guillot[2], Jun Xu[10], Dorothee Schwinge[1,5], Michael Trauner[5,11], Ansgar Wilhelm Lohse[1,5], Stefan Bonn[8], Norbert Hübner[3,12,13], Christoph Schramm[1,5,14,15,*]

JHEP Reports **2025**. vol. 7 | 1–11

Check for updates

Background & Aims: Primary sclerosing cholangitis (PSC) is a chronic heterogenous cholangiopathy with unknown etiology where chronic inflammation of the bile ducts leads to multifocal biliary strictures and biliary fibrosis with consecutive cirrhosis development. We here aimed to identify a PSC-specific gene signature associated with biliary fibrosis development.

Methods: We performed RNA-sequencing of 47 liver biopsies from people with PSC (n = 16), primary biliary cholangitis (PBC, n = 15), and metabolic dysfunction-associated steatotic liver disease (MASLD, n = 16) with different fibrosis stages to identify a PSC-specific gene signature associated with biliary fibrosis progression. For validation, we compared an external transcriptome data set of liver biopsies from people with PSC (n = 73) with different fibrosis stages (baseline samples from NCT01672853).

Results: Differential gene expression analysis of the liver transcriptome from patients with PSC with advanced *vs.* early fibrosis revealed 431 genes associated with fibrosis development. Of those, 367 were identified as PSC-associated when compared with PBC or MASLD. Validation against an external data set of 73 liver biopsies from patients with PSC with different fibrosis stages led to a condensed set of 150 (out of 367) differentially expressed genes. Cell type specificity assignment of those genes by using published single-cell RNA-Seq data revealed genetic disease drivers expressed by cholangiocytes (e.g. *CXCL1, SPP1*), fibroblasts, innate, and adaptive immune cells while deconvolution along fibrosis progression of the PSC, PBC, and MASLD samples highlighted an early involvement of macrophage- and neutrophil-associated genes in PSC fibrosis.

Conclusions: We reveal a PSC-attributed gene signature associated with biliary fibrosis development that may enable the identification of potential new biomarkers and therapeutic targets in PSC-related fibrogenesis.

© 2024 The Author(s). Published by Elsevier B.V. on behalf of European Association for the Study of the Liver (EASL). This is an open access article under the CC BY license (http://creativecommons.org/licenses/by/4.0/).

Introduction

Primary sclerosing cholangitis (PSC) is a chronic heterogenous cholangiopathy that is characterized by multifocal inflammation of bile ducts and subsequent biliary fibrosis.[1] Progression of fibrosis leads to end-stage liver disease in a considerable number of patients and together with hepatobiliary malignancy represents the main cause of mortality in PSC. To date, no clinically approved antifibrotic or causal treatments are available and liver transplantation remains the only effective therapy.[2] Pre-clinical rodent models including bile duct ligation and Mdr2-knockout mice have been developed to discover the mechanisms underlying chronic cholestasis and subsequent fibrosis. However, translation to human PSC has been hampered because of the many drawbacks of murine models based on the lack of understanding of disease pathogenesis in PSC.[3] Recent RNA-sequencing (RNA-Seq) studies aimed to decipher the complex cell composition in cirrhosis identifying multiple cell types of mesenchymal, endothelial, and myeloid origin.[4–6] However, those studies mostly focused on end-stage liver disease and neglected to examine earlier stages of fibrosis while often failing to include other non-PSC control cohorts.

In the current study, we used an unbiased RNA-Seq approach to identify a PSC-specific pro-fibrogenic gene signature. Thinking of fibrosis development as a therapeutic target, it is important to assess the progression from early fibrosis stages to advanced stage disease. We therefore analyzed the liver transcriptome in people with PSC with different stages of fibrosis and added biliary disease controls (primary biliary cholangitis [PBC]) and non-biliary controls (metabolic dysfunction-associated liver disease [MASLD]), to identify key drivers for PSC-specific biliary fibrosis progression.

Patients and methods

Patient population and liver samples

In our retrospective study 47 liver biopsies from people with PSC, PBC and MASLD who had undergone liver biopsy between 2011 and 2016 at the University Medical Center Hamburg-Eppendorf, Germany were included for RNA-Seq

* Corresponding authors. Address: I.Medizinische Klinik, Universitätsklinikum Hamburg-Eppendorf, Martinistraße 52, 20251 Hamburg, Germany; Tel.: +49 40 7410 52545 (C. Schramm); Tel.: +49 30 450 630127 (A. Laschtowitz).
E-mail addresses: alena.laschtowitz@charite.de (A. Laschtowitz), c.schramm@uke.de (C. Schramm).
https://doi.org/10.1016/j.jhepr.2024.101267

analysis. Diagnosis of the underlying chronic liver disease was based on clinical, biochemical, serological, radiological, and histopathological findings according to current guidelines[7–9] and biopsies were taken as part of routine clinical practice according to the standard procedure in our center via TruCut needle biopsy (TruCut, South Jordan, UT, USA) during a mini-laparoscopic procedure. Liver samples of all etiologies were categorized as early (Desmet/Scheuer stage 0–2/4) or advanced fibrosis (3–4/4) by local pathologists using the Desmet classification for better comparability.[10] Liver tissue was stored in liquid nitrogen for further analysis.

For the qPCR validation liver tissue from explanted livers of patients with PSC cirrhosis who had undergone liver transplantation at the University Medical Center Hamburg-Eppendorf between 2015 and 2018 and from margins of resected liver adenomas that were undertaken at the University Medical Center Hamburg-Eppendorf between 2015 and 2019 were used.

Clinical and biochemical parameters

We assessed data on clinical, biochemical and serological parameters at the time of liver biopsy. The following laboratory values were assessed: alanine aminotransferase (ALT), aspartate aminotransferase (AST), alkaline phosphatase (ALP), IgG, gamma-glutamyltransferase (γ-GT), albumin, bilirubin, creatinine, hemoglobin, platelet count, and international normalized ratio (INR).

RNA purification

Total RNA was isolated from frozen liver tissue using the NucleoSpinKit (Macherey-Nagel, Düren, Germany) and complementary DNA (cDNA) was revere-transcribed from total RNA (High capacity cDNA Reverse Transcriptase Kit, applied biosystems, by Thermo Fisher Scientific). Expression was measured using the Kappa probe Fast Universal qPCR mastermix in combination with Taqman probes (applied biosystems, by Thermo Fisher Scientific) that were used for amplification.

Validation with qPCR

To analyze the gene expression data, the ΔCt (delta Ct) method was used, whereby the Ct values of the target genes were normalized to the expression levels of the housekeeping gene Hypoxanthine Guanine Phosphoribosyltransferase (HPRT). This normalization approach allows for the comparison and interpretation of relative gene expression levels across different samples. The resulting values were reported as fold changes, indicating the increase or decrease in expression relative to the control samples. The fold changes were calculated using formula $2^{(-\Delta\Delta Ct)}$, which provides a quantitative measure of the relative changes in gene expression levels compared with the control group.

RNA-Seq and analyses

RNA quality was assessed using Bioanalyzer, and samples with and RNA integrity number >7 were included for RNA-Seq. Up to 1 µg RNA was used to synthesize mRNA libraries using TruSeq stranded mRNA library Preparation Kit (Illumina) on a Hiseq 4000 system (Illumina). We used FastQC (version 0.11.5, Babraham Institute, Babraham, UK) for a general quality check of the raw fastq files. TruSeq2-PE adapter and low quality read trimming was performed with Trimmomatic[11] (version 0.36) using the options ILLUMINACLIP:TruSeq2-PE.fa:2:30:10:2:falseSLIDINGWINDOW:4:15. Subsequently, the reads were aligned against the ensemble 87 reference genome and ensemble 87 reference annotation with STAR (version 2.7.3a).[12] On average read depth for the 47 samples was 40.310.540 (range: 8.318.152–61.856.384) and on average 92.76% (range: 79.27–95.88%) of the reads were uniquely mapped to the reference genome.

Further analysis was performed with R (version 3.6.3, R Foundation for Statistical Computing, Vienna, Austria). To filter the data, a threshold of ≥10 counts in all samples was set. We used DESeq2 for normalization and differential gene expression analysis.[13] Data were inspected for possible confounding effects using principal component analysis (PCA) based on regularized logarithm transformed counts. We compared different diseases and early *vs.* advanced stages of fibrosis across different etiologies including covariates for sex. We corrected for multiple testing with the Benjamini–Hochberg method. Adjusted p values of ≤0.05 were considered significantly different and an absolute cut-off $\log_2$ foldchange ($\log_2$FC) of 1 was set.

Pathway analysis and enrichment analysis

Pathway and enrichment analysis was performed using KEGG pathway analysis or GO term analysis via the ClusterProfiler package in the R environment. If reasonable, the simplify-function was used to remove redundancy of enriched GO terms.

Cell type mapping

Cell type mapping for the identified genes was determined by using a public available single-cell RNA-Seq data set only from lean people without chronic liver disease.[14] Dot size represents the fraction of cells within a cell type cluster where transcripts of the gene were detected, whereas only cell types that showed expression of the gene in at least 0.5% of the cell fraction are shown. Color scale illustrates enrichment of expression (fold-change; logFC) in relation to all other cell types. Cell type specificity was defined as 50% higher mRNA expression levels in comparison with cell types with the next highest mRNA expression.

Cell type mapping along fibrosis progression trajectory

Each gene weight was extracted per principal component (PC) describing the disease progression from fibrosis stages 0–4 for PSC, PBC, and MASLD respectively. For all genes, cell type specificity was calculated based on the fraction of single cell per cell type expressing the gene from a previously published scRNA-Seq study.[14] To map gene expression signatures onto specific cell types at various stages of fibrosis progression, we normalized the gene expression data from the published scRNA-Seq study by library size, performed a log transformation, and scaled the values for each gene to unit variance and zero mean. We then multiplied these normalized gene expression values by the gene weights in the PC. For each fibrosis-course describing PC, the cell type specificity for each gene was determined and ranked according to its coefficient. The same sign for each PC was chosen, so that negative signs

are associated with lower fibrosis scores, whereas positive signs indicate higher fibrosis scores.

Statistical analysis

Percentages and counts are given for categorical data. Median values with the corresponding range were calculated for continuous data. To test for differences between groups, non-parametric tests, including the Wilcoxon signed rank test, were performed. A comparison of categorical data between groups was performed using Pearson's X^2 test. All *p* values were two-tailed, a *p* value <0.05 was considered statistically significant. Figure design and statistical testing were carried out using R version 3.6.0 and R Studio version 1.2.1335.

Study approval

Informed written consent was obtained from each person. The study has been approved by the local ethics committee (ethics number PV4081).

Results

Patient characteristics at time of liver biopsy

In total, 47 liver samples from people with PSC (n = 16), PBC (n = 15), and MASLD (n = 16) with different fibrosis stages were included in the RNA-Seq analysis. Clinical characteristics of all patients are displayed in Table 1. Distribution of sex was different between people with PSC and PBC (PSC: 19% female, PBC: 87% female, *p* <0.001), and age differed between people with PSC and PBC (PSC median 37 years *vs.* PBC median 53 years, *p* <0.001) as well as MASLD (PSC median 37 years *vs.* MASLD median 49.5 years, *p* <0.001).

RNA-Seq of liver biopsies reveals etiology-specific pro-fibrogenic gene signatures

To explore a common gene signature for fibrosis development, we undertook RNA-Seq of liver biopsies from people with PSC, PBC, and MASLD. PCA for all samples revealed a clustering mainly based on the underlying disease and sex, as shown in Fig. 1 and

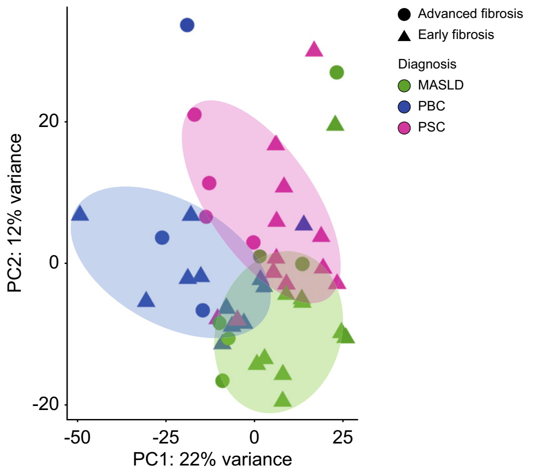

Fig. 1. Disease- and sex-based clustering of liver samples of patients with PSC, PBC, and MASLD after RNA-sequencing analysis. Principal component analysis after RNA-sequencing of liver samples of patients with PSC (n = 16), PBC (n = 15) and MASLD (n = 16) with advanced and early fibrosis. Counts of the filtered genes are used. MASLD, metabolic dysfunction-associated steatotic liver disease; PBC, primary biliary cholangitis; PC, principal component; PSC, primary sclerosing cholangitis.

Table 1. Clinical characteristics at the time of liver biopsy.

Parameters	PSC (n = 16)	PBC (n = 15)	MASLD (n = 16)	*p* value*	
				PSC *vs.* PBC	PSC *vs.* MASLD
Sex (female), n (%)	3 (19)	13 (87)	8 (50)	<0.001	0.137
Age (range), years	37 (18–59)	53 (40–82)	49.5 (23–73)	<0.001	0.01
Hemoglobin (range), g/dl	14.4 (10.4–16.3)	13.1 (9.5–15.9)	15.1 (12.5–16.3)	0.135	0.156
Platelets (range), 10^9/L	248 (82–448)	241 (125–448)	243 (48–368)	0.989	0.269
Albumin (range), g/L	38 (24–43)	35 (21–41)	38 (37–42)	0.173	0.39
Bilirubin (range), mg/dl	1 (0.3–9.9)	1.5 (0.4–3)	0.5 (0.3–2.3)	0.303	0.533
AST (range), U/L	88 (16–263)	74 (20–487)	40.5 (21–142)	0.489	0.037
ALT (range), U/L	100 (19–723)	61 (17–515)	72 (26–290)	0.406	0.052
γ-GT (range), U/L	450 (62–1,310)	245 (73–839)	117 (30–901)	0.205	0.081
ALP (range), U/L	336 (115–672)	231 (85–746)	96 (54–137)	0.809	<0.001
IgG (range), g/L	17.3 (10.6–23.8)	15.1 (8.7–46.5)	–	0.676	–
Creatinine (range), mg/dl	0.9 (0.4–1)	0.8 (0.6–1.1)	1 (0.7–1.2)	0.919	0.037
INR (range)	1 (0.9–1.3)	1 (0.9–1.1)	1 (0.8–1.4)	0.157	0.594
Fibrosis stage (Desmet *et al.*[15]), n (%)					
0	1 (6)	2 (13)	4 (25)		
1	7 (44)	5 (33)	5 (31)		
2	4 (25)	5 (33)	1 (6)		
3	2 (12.5)	0	4 (25)		
4	2 (12.5)	3 (20)	2 (13)		
Systemic immunosuppressive treatment, n (%)	4 (25)	3 (20)	0	0.679	<0.001
Treatment with UDCA, n (%)	8 (50)	10 (66)	0	0.565	<0.001
Chronic inflammatory bowel disease, n (%)	9 (69)	0	0	<0.001	<0.001

Median values are presented with range in brackets. *Continuous variables were compared using Wilcoxon singed rank test. Pearson's X^2 test was used for comparing percentages. ALT, alanine aminotransferase; ALP, alkaline phosphatase; AST, aspartate aminotransferase; γ-GT, gamma-glutamyltransferase; IgG, immunoglobulin G; INR, international normalized ratio; MASLD, metabolic dysfunction-associated steatotic liver disease; PBC, primary biliary cholangitis; PSC, primary sclerosing cholangitis; UDCA, ursodesoxycholic acid.

Fig. S1A. Other clinical characteristics such as age, immunosuppression or UDCA intake at the time of biopsy did not show a clear impact on PCA clustering, as shown in our Fig. S1A and B, and could therefore be neglected as covariates. Consequently, we included sex as covariate for our further differential gene expression analyses between the diseases. As expected, categorization of all patient samples into early fibrosis (stages 0–2) and advanced fibrosis (stages 3–4) according to Desmet *et al.*[15] without considering the underlying etiology did not result in a clear clustering between people with advanced or early fibrosis, as shown in Fig. 1, indicating strong disease-specific pro-fibrogenic mechanisms. Accordingly, we continued our analysis comparing the samples with advanced *vs.* early fibrosis strictly classified by the underlying etiology. Clinical characteristics within the groups are displayed in Table 2.

Identification of a PSC-attributed gene signature in biliary fibrosis

To identify a PSC-specific pro-fibrogenic gene set, we categorized the samples of people with PSC into early (stages 0–2) and advanced fibrosis (stages 3–4) and the PCA showed a clear clustering based on the fibrosis score along the first principal component (PC1) as displayed in Fig. 2A. After differential gene expression analysis comparing the PSC samples with advanced fibrosis against early fibrotic PSC samples, 431 differentially expressed genes (DEGs) were revealed (threshold: p-adj ≤0.05, $\log_2$FC ≥|-1|). Results are displayed in a volcano plot in Fig. 2B and in Table S1. Gene ontology enrichment analysis identified expected biological processes such as *extracellular matrix organization* and the *collagen metabolic process* while KEGG pathway analyses revealed cellular processes such as *ECM-receptor interaction* and pathways linked with cancer such as *PI3K-AKT signaling pathway* as displayed in Fig. 2C and D. Similarly, we categorized the samples of patients with PBC or MASLD into early and advanced fibrosis and analyzed them separately. The samples of patients with PBC showed a less stringent clustering based on fibrosis stages in the PCA, as shown in Fig. S2A, and only 41 genes were differentially expressed between advanced and early fibrotic PBC samples, displayed in Table S2, which may relate to the smaller sample size of PBC livers with advanced fibrosis included. However, the samples of people with MASLD showed a clear clustering according to fibrosis stages, as displayed in Fig. S2B with 462 genes being differentially expressed between the two fibrosis categories, as listed in Table S3.

After identifying the DEGs between advanced *vs.* early fibrosis within each disease cohort separately, we aimed to analyze the unique features and overlaps of the disease-specific pro-fibrogenic gene sets: The majority of PSC-associated differentially expressed pro-fibrogenic genes (367/431 DEGs), were specifically seen in PSC fibrosis progression but not in the control cohorts, as displayed in Fig. 3A illustrating a strong etiology-dependent influence on biliary fibrosis progression. Gene set enrichment analysis of those 367 DEGs identified biological processes such as the extracellular matrix organization, but also leukocyte migration, as shown in Fig. S3.

Only four genes were shown to be pro-fibrogenic genes between all three diseases (*ASPHD1, DMKN, MUC13, ST14*). Amongst them was DMKN, encoding for the protein Dermokine, which had recently been identified as a regulator for hepatic stellate cell (HSC) activation,[16] the main cellular players in liver fibrosis. Both chronic cholestatic diseases, PBC and PSC, shared additional two genes between their pro-fibrogenic DE gene sets: *SCUBE2* and *SPINK1*.

External validation confirms PSC-associated pro-fibrogenic gene signature

In a next step, we aimed to validate our findings by means of an external validation cohort. In a recently published paper, Gindin *et al.*[17] performed bulk RNA-Seq of 74 liver biopsies from people with PSC with different fibrosis stages (baseline samples from NCT01672853) which clustered based on their degree of fibrosis (along PC1). Given the fibrosis score according to the Ishak classification,[10] we subdivided the samples into advanced fibrosis (4–6/6) and early fibrosis (0–3/6) and performed the differential gene expression analysis with DESeq2 on those samples with sex as covariate according to our first analysis (threshold: p-adj ≤0.05, $\log_2$FC ≥|-1|). In total, 1,351 genes were differentially expressed between PSC samples with advanced *vs.* early fibrosis contributing to biological processes such as *extracellular matrix organization* in the gene ontology enrichment analysis (Fig. S4). We compared this DE gene set to our DEGs generated from liver biopsies of patients with PSC with advanced *vs.* early fibrosis (Table S1). Indeed, 150 DEGs were shared between those two

Table 2. Clinical characteristics at the time of liver biopsy of study patients divided into advanced (stage 3–4) and early fibrosis (stage 0–2).

	PSC			PBC			MASLD		
	Advanced fibrosis (n = 4)	Early fibrosis (n = 12)	*p* value*	Advanced fibrosis (n = 3)	Early fibrosis (n = 12)	*p* value*	Advanced fibrosis (n = 6)	Early fibrosis (n = 10)	*p* value*
Sex (female), n (%)	1 (25)	2 (17)	0.712	3 (100)	10 (83)	0.448	3 (50)	5 (50)	1
Age (range), years	37 (24–48)	35 (18–59)	0.969	55 (53–66)	49 (40–82)	0.379	65.5 (48–73)	48.5 (23–60)	0.251
Systemic immunosuppressive treatment, n (%)	1 (25)	4 (36)	0.679	0	3 (25)	0.333	0	0	–
MELD	11.5 (9–14)	–	–	10 (6–10)	–	–	8 (7–15)	–	–
Child–Pugh	6.5 (5–8)	–	–	5 (5–7)	–	–	5 (5–6)		
Presence of varices	1 (25)	–	–	0	–	–	1 (17)	–	–
Episode of decompensation at time of biopsy	0	–	–	0	–	–	1† (17)	–	–
Percentage of steatosis (%)	–	–	–	–	–	–	10 (10)	30 (10–60)	0.037
NAFLD activity score	–	–	–	–	–	–	3 (3–5)	4 (0–5)	0.292

Median values are presented with range in brackets. MASLD, metabolic dysfunction-associated steatotic liver disease; NAFLD, non-alcoholic fatty liver disease; PBC, primary biliary cholangitis; PSC, primary sclerosing cholangitis.
*Continuous variables were compared using Wilcoxon singed rank test. Pearson's X^2 test was used for comparing percentages.
†One patient with ascites and variceal bleeding at time of biopsy (2 weeks around biopsy).

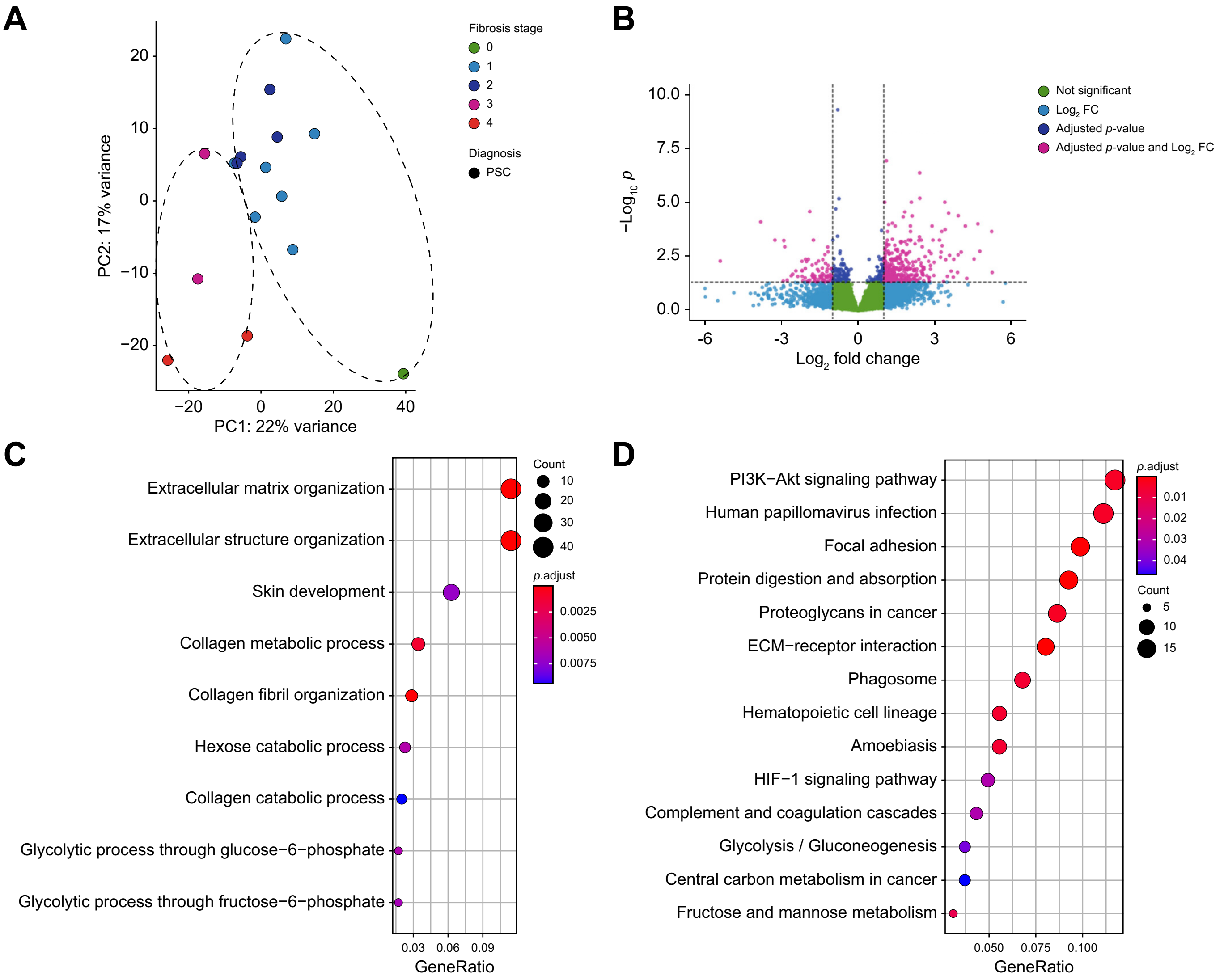

Fig. 2. Differential gene expression analysis between advanced and early fibrosis in patients with PSC. (A) Principal component analysis after RNA-sequencing of liver samples of patients with PSC (n = 16) subdivided into advanced (3–4/4) and early (0–2/4) fibrosis. Corrected counts of the filtered genes are used. (B) Volcano plot illustrating genes from the filtered data set, that show significant differential expression between advanced and early fibrosis in 16 patients with PSC. (C) Gene ontology term enrichment analysis for the biological processes of the 431 DEGs between advanced and early fibrosis stage in patients with PSC (n = 16). (D) KEGG pathway analysis of the 431 DEGs between advanced and early fibrosis stage in patients with PSC (n = 16). PC, principal component; PSC, primary sclerosing cholangitis.

data sets contributing to biological processes such as *extracellular matrix organization* (Fig. S5), and molecular functions as *Wnt-protein binding* in gene ontology enrichment analysis. Accordingly, we found several genes amongst the 150 PSC-specific DEGs, being associated with *Wnt signaling pathway* (e.g. *AEBP1, CTHRC1, FZD2, FZD7, PTK7, ROR2, SFRP4*). Of note, the established fibrosis driver TGF-beta was differentially expressed in the external data set in PSC fibrosis but not in our analysis. Nevertheless, multiple genes known to be involved in TGF-beta signaling were detected within the 150 overlapping DEGs (*CTHRC1, FZD2, GLI2, MFAP2, PDLIM7, PMP22, PTK7, SPECC1*).

Further, according to KEGG pathway analysis, several genes were linked to the *PI3K/Akt pathway* (*BRSK1, CCND2, COL1A1, COL4A1, COL4A2, COMP, CXCL1, FGF7, LAMC3, SLCO4A2, SPP1*), which controls cellular processes such as cell division, survival, and differentiation.

Cell type specificity assignment deciphers complexity of cell composition in biliary fibrosis

To enable assignment of the validated 150 PSC-specific fibrosis-associated genes to specific cell types, we used published single-cell RNA-Seq data of human livers from lean people without chronic liver disease.[14] A total of 147 out of 150 genes could be attributed within the single-cell RNA-Seq data to the different cell types as described in the Patients and methods section. Of those, 39 showed fibroblast specificity, as displayed in Fig. 3B, amongst them well characterized genes mostly coding for extracellular matrix (ECM) proteins (e.g. *COL1A1, COL1A2, FBLN1, LUM*) or genes being previously associated to non-PSC liver fibrosis such as *AEBP1*,[18] *MFAP4*,[19,20] and *NFATC4*.[21]

Although 12 genes were assigned to cholangiocytes, other genes could be assigned to innate and adaptive immune cells,

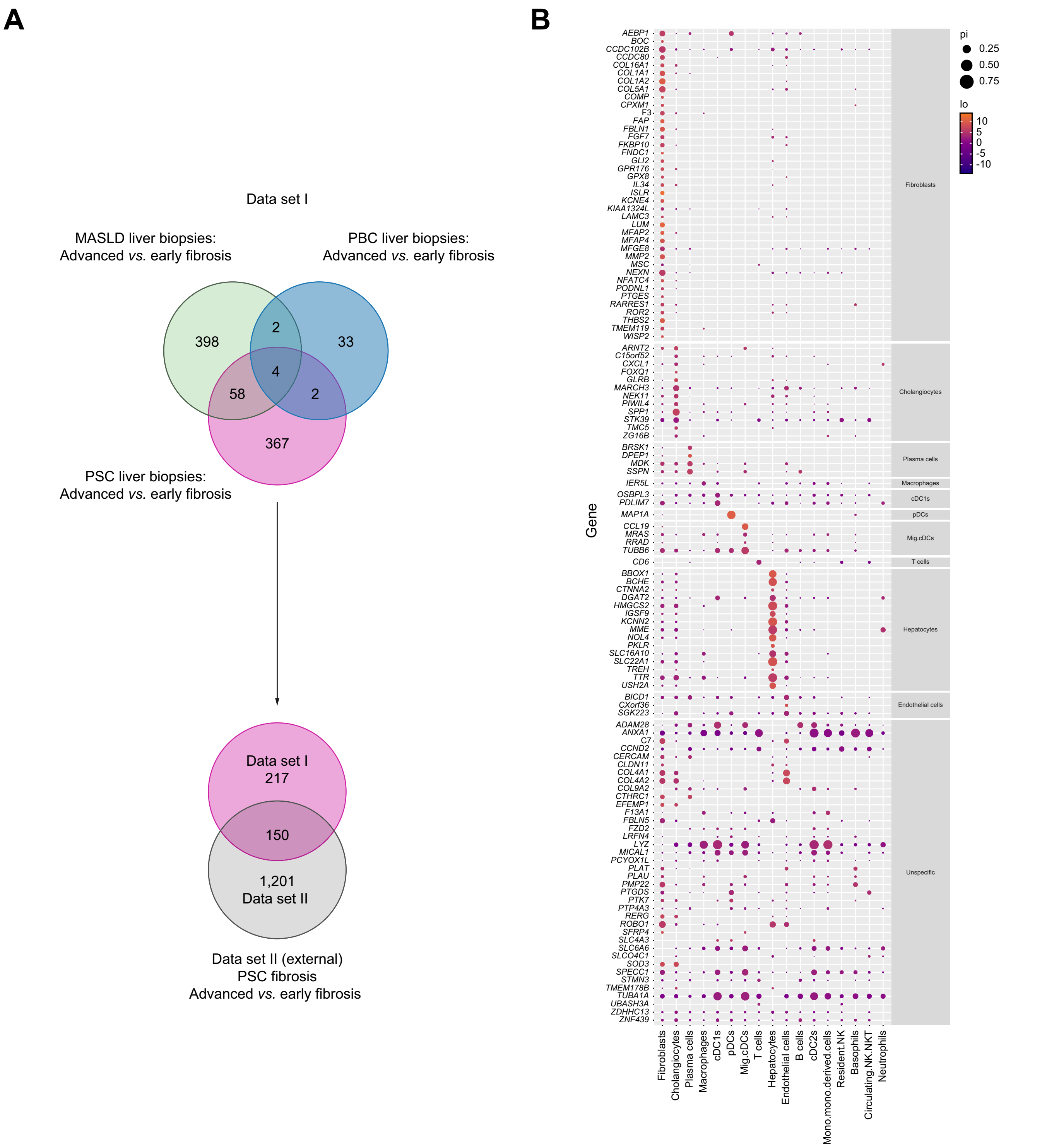

Fig. 3. Validated PSC-specific pro-fibrogenic DEGs and their cell type specificity. (A) First Venn diagram showing the overlap and disease-specific DEGs of advanced *vs.* early fibrosis comparison in PSC, PBC, and MASLD in data set I. Second Venn diagram highlighting the overlap of PSC-specific pro-fibrogenic DEGs in data set I and external data set II. (B) Mapping cell type specificity of the 150 PSC-specific pro-fibrogenic genes as overlap from both data sets by using published single-cell RNA-Seq data.[14] Dot size represents the fraction of nuclei within a cell type where transcripts of the gene were detected, while only cell types that showed expression of the gene in at least 0.5% of the cell type fraction are shown. Color scale illustrates enrichment of expression (foldchange; logFC) compared with all other cell types. Cell type specificity was defined as 50% higher mRNA expression levels in comparison with cell types with next the highest mRNA expression. cDC1, Type-I conventional dendritic cell; DEG, differentially expressed genes; mig. cDC, migratory conventional dendritic cell; MASLD, metabolic dysfunction-associated steatotic liver disease; PBC, primary biliary cholangitis; pDC, plasmacytoid dendritic cell; PSC, primary sclerosing cholangitis.

notably plasma cells, T lymphocytes, and macrophages and genes attributed to different subsets of dendritic cells such as type 1 conventional dendritic cells (cDC1) and plasmacytoid DCs (pDC), underlining the complex cell composition and interplay in PSC-associated biliary fibrosis progression. For a further 38 genes, a cell type-specific assignment was not easily possible, as the expression was detectable in several cell types and we decided to label them as unspecific.

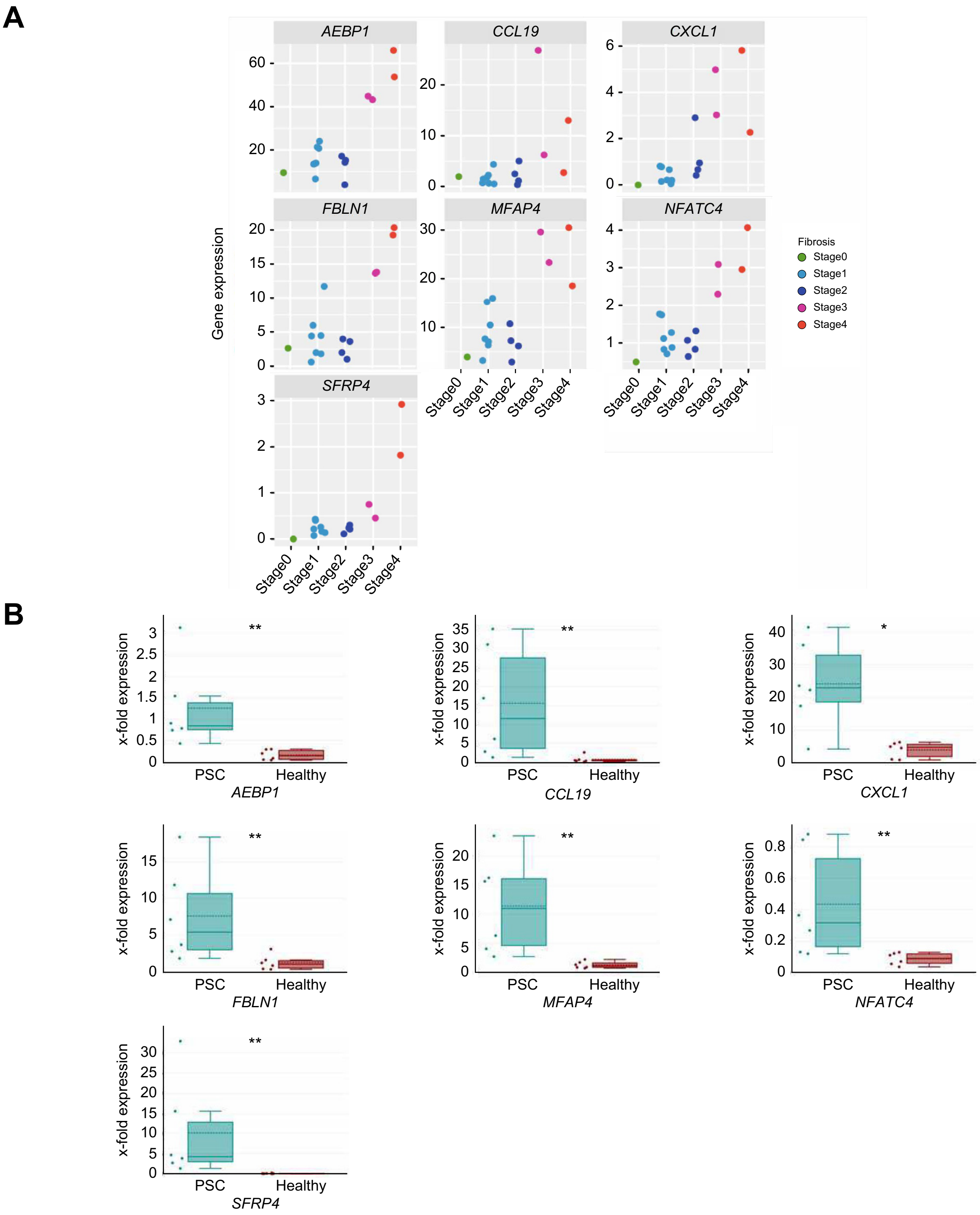

Fig. 4. Gene expression along fibrosis progression and validation via qPCR of genes associated with PSC-specific fibrosis progression. (A) Display of the normalized counts (rpm, reads per million) of seven genes from each PSC liver biopsy with the according fibrosis stage. (B) Boxplots representing the gene expression of different genes in liver tissue from persons with PSC cirrhosis (n = 6) or healthy controls (n = 6). Horizontal bars indicating median and IQR, unpaired two-sided Wilcoxon test was performed (*p ≤0.05, **p ≤0.01). PSC, primary sclerosing cholangitis.

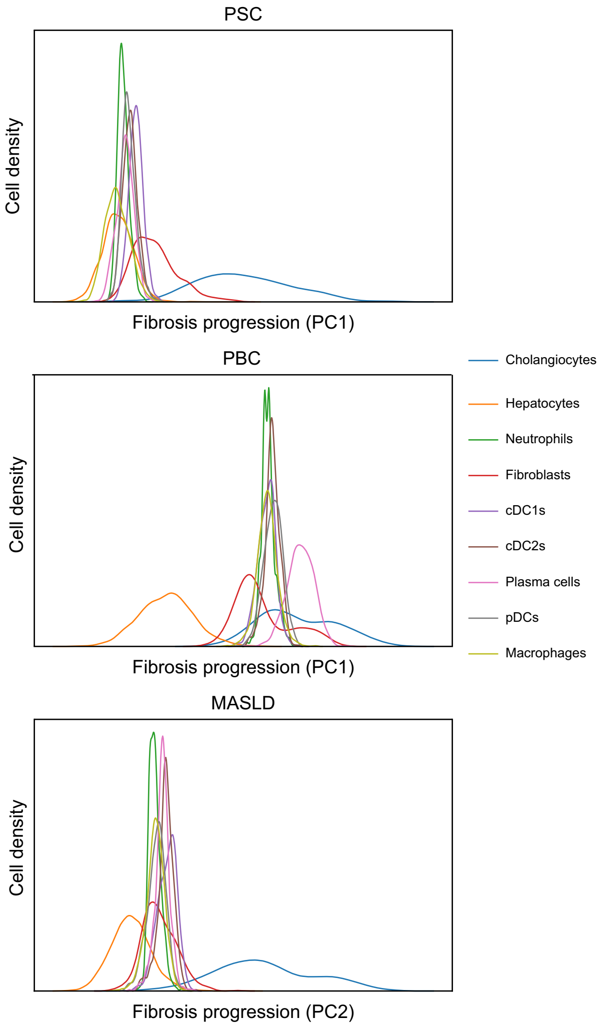

Fig. 5. Cell type-specific enrichment over the time course of fibrosis development. Density distributions show cell type scoring along the fibrosis trajectories in PSC, PBC, or MASLD as obtained from the principal component analysis as displayed in Fig. 2A for PSC (along PC1), Fig. S2A for PBC (along PC1) and Fig. S2B for MASLD (along PC2). cDC1, Type-I conventional dendritic cell; mig. cDC, migratory conventional dendritic cell; MASLD, metabolic dysfunction-associated steatotic liver disease; PBC, primary biliary cholangitis; PC, principal component; pDC, plasmacytoid dendritic cell; PSC, primary sclerosing cholangitis.

Next, we identified those seven out of 150 genes with a steady increase in expression across the PSC fibrosis stages, as shown in Fig. 4A: *AEBP1, CCL19, CXCL1, FBLN1, MFAP4, NFATC4*, and *SFRP4*. The qPCR analysis of liver tissue samples from an independent cohort of persons with PSC cirrhosis (n = 6) and healthy controls (n = 6) validated an upregulated gene expression as displayed in Fig. 4B.

Macrophage-, neutrophil-, and hepatocyte-associated genes are enriched in PSC fibrosis initiation

Considering the low overlap of DEGs from advanced to early fibrosis between PSC, PBC, and MASLD, we wanted to investigate potential cellular drivers of fibrogenesis in PSC by assigning cell type specificity for the enriched genes during fibrosis progression within each disease cohort. For this aim, we first looked at the PCA of the three different diseases and noticed that the fibrosis progression was mostly attributed to the PC1 in PSC and PBC (Fig. 2A and Fig. S2A) and to the PC2 in MASLD (Fig. S2B). We extracted the gene weights of the PCs, respectively, as described above in the methods section and subsequently determined the enrichment of cell type specific gene signatures during fibrosis progression. Finally, we ended up with a density plot showing the assumed contribution of cell types using gene weights during fibrosis progression in PSC, PBC, and MASLD in comparison (Fig. 5). While in early phases of fibrosis in PBC and MASLD mainly hepatocyte-specific genes were enriched, in PSC also macrophages and neutrophils were already assigned at earlier time points, which may indicate an important role of macrophages and neutrophils in PSC fibrosis initiation. Our analyses also suggest an earlier contribution of fibroblast-specific genes in PBC compared with PSC, while in PBC, plasma cell-specific genes appear to be enriched rather late in fibrosis compared with PSC and MASLD. A common factor of all three diseases was that cholangiocyte-specific genes accumulate in late fibrosis stages.

Discussion

Development of biliary fibrosis with progression to cirrhosis is one of the major life-limiting consequences of PSC.[1,22] It is hypothesized that PSC fibrosis is driven by a complex interplay between

inflammatory and parenchymal cells as hepatocytes and cholangiocytes, but to date, the detailed underlying pathomechanisms leading from initial insults to peribiliary fibrosis with subsequent cirrhosis development are still unknown.[1] Recently, several studies have used extensive proteomic and transcriptomic analyses at the single-cell level to demonstrate the complexity and heterogeneity of immune cell composition and their potential interaction with parenchymal and non-parenchymal cells in chronic liver injury and fibrosis.[4–6,14,23] However, these studies mostly focused on advanced fibrosis and often lacked appropriate control cohorts. By incorporating all stages of PSC fibrosis and comparing with fibrosis development in PBC and MASLD (Fig. 1), our bulk RNA-Seq study provides an unbiased comprehensive transcriptome data set identifying 367 DEGs associated with PSC fibrosis progression (Fig. 2). Further, we validated our analysis with the help of an external, independent dataset of PSC liver-derived transcriptomes thus being able to present a condensed set of 150 genes associated with PSC-specific fibrosis progression. Using published single-cell data, we attributed those genes to specific cell types (Fig. 3). Accordingly, this analysis highlighted the complexity and heterogeneity of cell type composition in PSC fibrosis: Besides cholangiocyte- and fibroblast-specific genes, other genes attributed to plasma cells, T lymphocytes, macrophages and several dendritic cell subtypes. Finally, we compared the contribution of genes along fibrosis progression combined with cell type annotation analysis between PSC, PBC, and MASLD. This indicates an early involvement of macrophages, neutrophils, and hepatocytes in the initiation phase of PSC fibrosis, whereas in PBC and MASLD hepatocyte-associated genes were enriched during early stages of fibrosis (Fig. 5).

The implementation of an external validation cohort led to a condensed data set of 150 DEGs associated with PSC fibrosis. Our analyses provide compelling evidence for the involvement of various signaling pathways such as *Wnt* and *PI3K/Akt*, which represent promising targets for future mechanistic studies. The latter appears to be particularly important for macrophage differentiation in chronic liver disease as recently elucidated.[24] Accordingly, our data suggest TGF-beta involvement in PSC fibrosis. TGF-beta interacts with the *canonical Wnt/beta-catenin pathway*, whose selective inhibition was shown to decrease inflammatory processes and to reduce growth of activated HSCs and collagen synthesis, thus decelerating the progression of liver fibrosis.[25] The gene *LOXL2*, which promotes collagen crosslinking in liver fibrogenesis,[26] was found as differentially expressed in our PSC-specific gene set but not in the external cohort. This may be because of differences in sample acquisition and sequencing performed.

The annotation of those genes to the different cell types deciphered the potential cell types involved in PSC fibrosis and underlined the complexity of the cellular landscape in PSC fibrosis development. Twelve genes were assigned to cholangiocytes with *SPP1* as a well-known marker for activated cholangiocytes. *SPP1* encodes the chemokine osteopontin and has been linked to hepatic fibrosis via activation of HSC[26] but was recently also shown as marker for recruited lipid-associated macrophages,[27] underlining the important role of the heterogenous population of myeloid cells in fibrosis progression.

Although cell type-specific assignment was not possible for several genes, some of them had already been attributed to non-PSC liver fibrosis such as *CTHRC1*[28] or to non-liver fibrosis such as *SFRP4*.[29,30] We also found *TMEM178B*, which has recently been discovered as being upregulated in biliary fibrosis in people with biliary atresia.[31]

Moreover, our analyses shed light on new aspects of immune cell populations possibly involved. Our group could recently show that type 2 conventional dendritic cells (cDC2s) may play a role in cholangitis pathogenesis.[32] Interestingly, the present data herein suggest that in PSC fibrosis, also pDCs, conventional DCs (cDC1), and migratory conventional DCs (mig. cDC) may be involved in fibrosis progression.

Macrophage- and neutrophil-associated genes seemed to be involved already at earlier time points of fibrogenesis in PSC as compared with PBC and MASLD. Recently published data from Guillot *et al.*[23] may support this finding. Further, Govaere *et al.*[33] found that in PSC the peribiliary invasion with macrophages occurred already in the early stages of PSC, in contrast to chronic HCV hepatitis. Neutrophils have pathogenic and protective effects in liver fibrosis: they secrete IL-17, thereby upregulating the expression of the TGF-beta receptor in HSCs.[34] IL-17A promotes the recruitment of neutrophils in the liver and favors liver fibrosis in the model of bile duct ligation in mice.[35] In patients with PSC, it has recently been shown that neutrophils infiltrate the biliary microenvironment.[36] Accordingly, we found *CXCL1* increased in PSC fibrosis progression which is known to be involved in the recruitment especially of neutrophils and which has recently been shown to participate in early inflammatory responses and biliary proliferation via the inflammatory CXCL1–CXCR2–neutrophil axis induced by Hedgehog signaling in a mouse model of extrahepatic bile duct ligation.[37] Additionally, it has been described that CXCL1 expression can be regulated by TH17 cells which are increased in PSC.[38,39] In addition, we found *DPEP1* to be upregulated in PSC fibrotic livers, which may also have a role in neutrophil recruitment in lungs and liver.[40] Taken together, our results suggest the contribution of neutrophils in PSC fibrosis initiation.

Notably, there was small overlap of genes associated with fibrosis progression between PSC, PBC, or MASLD, highlighting the strong etiology-specific mechanisms of fibrogenesis. Nevertheless, PSC and PBC shared two further profibrogenic genes: *SPINK1* and *SCUBE2*. The former has been attributed to chronic pancreatitis,[41] idiopathic pulmonary fibrosis,[42] and hepatocellular carcinoma.[43] The latter belongs to a secreted and membrane-associated multi-domain protein family which was found to play a role in the Hedgehog signaling pathway, a critical regulator in liver fibrosis.[44] Interestingly, in a mouse model of cholestasis, Hedgehog signaling was found to demarcate a niche of fibrogenic peribiliary mesenchymal cells, indicating that the pathway might play an important role in cholestatic liver fibrosis.[22,45] Nevertheless, the small overlap between PSC- and PBC-induced fibrotic gene signatures might not only be caused by already known differences in pathomechanisms – the typical periductular fibrosis in PSC in contrast to the lymphoplasmacellular infiltrates initiating bile duct damage in the portal tracts of PBC livers[46] – but also attributable to a small sample size of PBC samples with advanced fibrosis.

Several strengths and limitations of our study need to be mentioned. Our analysis provides an unbiased approach that identifies the important gene signatures of biliary fibrosis development and offers the possibility to focus on a PSC-related fibrosis gene set by including different comparison

groups and an external validation cohort. Nevertheless, not all fibrosis stages could be equally represented in the PBC group, with only two samples with advanced fibrosis, which may contribute to the small overlap of the DEGs between PSC and PBC fibrosis progression. In addition, the relatively small overlap of the two different data sets is striking, which is mainly attributable to technical differences (e.g. biopsy technique, sequencing technique, *etc*.). Although we provide a method for the interpretation of unbiased bulk RNA-Seq data that allows the assignment of genes to specific cell types, our cell type assignment analysis is based on the fact that the reference data underweigh hepatocyte and cholangiocyte populations because of the underlying single-cell RNA-Seq technique and that data derives from liver samples of people without chronic liver diseases.[14] A similar bias is also present in the PCA of bulk RNA-Seq data, with gene expression values of lowly abundant cell types less included compared with higher abundant cell types such as hepatocytes.

Moreover, our bulk RNA-Seq data only measures the average expression level of a population of cells, information on the heterogeneity of the cell population is unavailable. Therefore, further unbiased single-cell studies, such as single nuclei RNA-Seq, are needed to study the role of rare immune cell populations and parenchymal cells at different fibrosis stages whereas spatial RNA-Seq is needed to determine the interaction of the different cell types. Lastly, our analysis is restricted to mRNA expression data which require validation at the protein level. Given the small amount of tissue obtained by needle biopsy in our study, no further material was available for validation of protein expression.

In summary, we provide an unbiased study of genes expressed in human livers at different fibrosis stages. We reveal PSC-associated gene signatures of fibrosis as a resource that will enable validation studies, as well as mechanistic studies needed to understand the differences and similarities of fibrosis progression between different liver diseases.

Affiliations

[1]*Department of Medicine, University Medical Center Hamburg-Eppendorf, Hamburg, Germany;* [2]*Department of Hepatology and Gastroenterology, Charité - Universitätsmedizin Berlin, Berlin, Germany;* [3]*Cardiovascular and Metabolic Sciences, Max-Delbrueck-Center for Molecular Medicine, Berlin, Germany;* [4]*Berlin Institute of Health (BIH), Berlin, Germany;* [5]*European Reference Network for Hepatological Diseases (ERN-RARE LIVER), Hamburg, Germany;* [6]*Department of Medicine I, LMU University Hospital, LMU Munich, Munich, Germany;* [7]*Gene Center, Department of Biochemistry, Ludwig Maximilians Universität, Munich, Germany;* [8]*Institute of Medical Systems Biology, Center for Molecular Neurobiology, University Medical Center Hamburg-Eppendorf, Hamburg, Germany;* [9]*Bioinformatics Core, University Medical Center Hamburg-Eppendorf; Hamburg, Germany;* [10]*Department of Biomarker Sciences, Gilead Sciences Inc., San Mateo, California, United States of America;* [11]*Division of Gastroenterology and Hepatology, Department of Medicine III, Medical University of Vienna, Vienna, Austria;* [12]*German Center for Cardiovascular Research (DZHK), partner site Berlin, Berlin, Germany;* [13]*Charité - Universitätsmedizin Berlin, Berlin, Germany;* [14]*Martin-Zeitz-Center for Rare Diseases, University Medical Center Hamburg-Eppendorf, Hamburg, Germany;* [15]*Hamburg Center for Translational Immunology (HCTI), Hamburg, Germany*

Abbreviations

γ-GT, gamma-glutamyltransferase; ALP, alkaline phosphatase; ALT, alanine aminotransferase; AST, aspartate aminotransferase; cDC1, type 1 conventional dendritic cell; cDC2, type 2 conventional dendritic cell; DEG, differentially expressed genes; ECM, extracellular matrix; HSC, hepatic stellate cell; INR, international normalized ratio; log_2FC, log_2(foldchange); MASLD, metabolic dysfunction-associated steatotic liver disease; mig. cDC, migratory conventional dendritic cell; NAFLD, non-alcoholic fatty liver disease; PBC, primary biliary cholangitis; PC, principal component; PCA, principal component analysis; pDC, plasmacytoid dendritic cell; PSC, primary sclerosing cholangitis; RNA-Seq, RNA-sequencing.

Financial support

The project was supported by the German Research Foundation (DFG, Deutsche Forschungsgemeinschaft (SFB841, KFO250, KFO306, No 290522633), 'YAEL Foundation', the 'Helmut and Hannelore Greve Foundation'. AL was supported by the Clinician Scientist Program of the KFO306 and the Digital Clinician Scientist Program of the Berlin Institute of Health.

Conflicts of interest

Please refer to the accompanying ICMJE disclosure forms for further details.

Authors' contributions

Substantial contribution to the conception and design: AL, CS. Data acquisition: AL, CS, ELL, SS. Bioinformatical analysis: AL, AML, CC, ELL, JX. Interpretation of data: AL, AG, ELL, LAL, CS. Drafting of the article: AL, CS. Critical revision: AML, AWL, CS, DS, ELL, MT, NH, SB.

Data availability statement

The transcriptome data are available at the following link: https://zenodo.org/records/13990103.

Acknowledgements

We are grateful for excellent technical assistance by Marko Hilken, Nina Verse, Angelika Schmidt, and Jennifer Wigger. The graphical abstract was created with BioRender.com.

Supplementary data

Supplementary data to this article can be found online at https://doi.org/10.1016/j.jhepr.2024.101267.

References

Author names in bold designate shared co-first authorship

[1] Karlsen TH, Folseraas T, Thorburn D, et al. Primary sclerosing cholangitis – a comprehensive review. J Hepatol 2017;67:1298–1323.
[2] Penz-Österreicher M, Österreicher CH, Trauner M. Fibrosis in autoimmune and cholestatic liver disease. Best Pract Res Clin Gastroenterol 2011;25:245–258.
[3] Mariotti V, Cadamuro M, Spirli C, et al. Animal models of cholestasis: an update on inflammatory cholangiopathies. Biochim Biophys Acta Mol Basis Dis 2019:1865954–1865964.
[4] Ramachandran P, Dobie R, Wilson-Kanamori JR, et al. Resolving the fibrotic niche of human liver cirrhosis at single-cell level. Nature 2019;575:512–518.
[5] **Poch T**, **Krause J**, Casar C, et al. Single-cell atlas of hepatic T cells reveals expansion of liver-resident naive-like CD4+ T cells in primary sclerosing cholangitis. J Hepatol 2021;75:414–423.
[6] Chung BK, Øgaard J, Reims HM, et al. Spatial transcriptomics identifies enriched gene expression and cell types in human liver fibrosis. Hepatol Commun 2022;6:2538–2550.
[7] European Association for the Study of the Liver (EASL), European Association for the Study of Diabetes (EASD), European Association for the Study of Obesity (EASO). EASL-EASD-EASO clinical practice guidelines for the management of non-alcoholic fatty liver disease. Obes Facts 2016;9:65–90.
[8] Chazouilleres O, Beuers U, Bergquist A, et al. EASL clinical practice guidelines on sclerosing cholangitis. J Hepatol 2022;77:761–806.

[9] Hirschfield GM, Beuers U, Corpechot C, et al. EASL Clinical Practice Guidelines: the diagnosis and management of patients with primary biliary cholangitis. J Hepatol 2017;67:145–172.
[10] Ishak K, Baptista A, Bianchi L, et al. Histological grading and staging of chronic hepatitis. J Hepatol 1995;22:696–699.
[11] Bolger AM, Lohse M, Usadel B. Trimmomatic: a flexible trimmer for Illumina sequence data. Bioinformatics 2014;30:2114–2120.
[12] Dobin A, Davis CA, Schlesinger F, et al. STAR: ultrafast universal RNA-seq aligner. Bioinformatics 2013;29:15–21.
[13] Love MI, Huber W, Anders S. Moderated estimation of fold change and dispersion for RNA-seq data with DESeq2. Genome Biol 2014;15:550.
[14] Guilliams M, Bonnardel J, Haest B, et al. Spatial proteogenomics reveals distinct and evolutionarily conserved hepatic macrophage niches. Cell 2022;185:379. 96.e38.
[15] Desmet VJ, Gerber M, Hoofnagle JH, et al. Classification of chronic hepatitis: diagnosis, grading and staging. Hepatology 1994;19:1513–1520.
[16] **Huang HM**, **Zhou XR**, Liu YJ, et al. Histone deacetylase inhibitor givinostat alleviates liver fibrosis by regulating hepatic stellate cell activation. Mol Med Rep 2021;23:305.
[17] Gindin Y, Chung C, Jiang Z, et al. A fibrosis-independent hepatic transcriptomic signature identifies drivers of disease progression in primary sclerosing cholangitis. Hepatology 2021;73:1105–1116.
[18] Gerhard GS, Hanson A, Wilhelmsen D, et al. AEBP1 expression increases with severity of fibrosis in NASH and is regulated by glucose, palmitate, and miR-372-3p. PLoS One 2019;14:e0219764.
[19] Madsen BS, Thiele M, Detlefsen S, et al. Prediction of liver fibrosis severity in alcoholic liver disease by human microfibrillar-associated protein 4. Liver Int 2020;40:1701–1712.
[20] **Bracht T**, **Mölleken C**, Ahrens M, et al. Evaluation of the biomarker candidate MFAP4 for non-invasive assessment of hepatic fibrosis in hepatitis C patients. J Transl Med 2016;14:201.
[21] Du M, Wang X, Yuan L, et al. Targeting NFATc4 attenuates non-alcoholic steatohepatitis in mice. J Hepatol 2020;73:1333–1346.
[22] **Wu H**, **Chen C**, Ziani S, et al. Fibrotic events in the progression of cholestatic liver disease. Cells 2021;10:1107.
[23] Guillot A, Winkler M, Silva Afonso M, et al. Mapping the hepatic immune landscape identifies monocytic macrophages as key drivers of steatohepatitis and cholangiopathy progression. Hepatology 2023;78:150–166.
[24] Yang Y, Jia X, Qu M, et al. Exploring the potential of treating chronic liver disease targeting the PI3K/Akt pathway and polarization mechanism of macrophages. Heliyon 2023;9:e17116.
[25] **Duspara K**, **Bojanic K**, Pejic JI, et al. Targeting the Wnt signaling pathway in liver fibrosis for drug options: an update. J Clin Transl Hepatol 2021;9:960.
[26] **Pollheimer MJ**, **Racedo S**, Mikels-Vigdal A, et al. Lysyl oxidase-like protein 2 (LOXL2) modulates barrier function in cholangiocytes in cholestasis. J Hepatol 2018;69:368–377.
[27] Remmerie A, Martens L, Thoné T, et al. Osteopontin expression identifies a subset of recruited macrophages distinct from Kupffer cells in the fatty liver. Immunity 2020;53:641. 57.e14.
[28] Li J, Wang Y, Ma M, et al. Autocrine CTHRC1 activates hepatic stellate cells and promotes liver fibrosis by activating TGF-β signaling. EBioMedicine 2019;40:43–55.
[29] **Gay D**, **Ghinatti G**, Guerrero-Juarez CF, et al. Phagocytosis of Wnt inhibitor SFRP4 by late wound macrophages drives chronic Wnt activity for fibrotic skin healing. Sci Adv 2020;6:3704.
[30] Bläuer M, Laaninen M, Sand J, et al. Wnt/β-catenin signalling plays diverse functions during the process of fibrotic remodelling in the exocrine pancreas. Pancreatology 2019;19:252–257.
[31] Kyrönlahti A, Godbole N, Akinrinade O, et al. Evolving up-regulation of biliary fibrosis–related extracellular matrix molecules after successful portoenterostomy. Hepatol Commun 2021;5:1036.
[32] Müller AL, Casar C, Preti M, et al. Inflammatory type 2 conventional dendritic cells contribute to murine and human cholangitis. J Hepatol 2022;77:1532–1544.
[33] Govaere O, Cockell S, Van Haele M, et al. High-throughput sequencing identifies aetiology-dependent differences in ductular reaction in human chronic liver disease. J Pathol 2019;248:66–76.
[34] Fabre T, Molina MF, Soucy G, et al. Type 3 cytokines IL-17A and IL-22 drive TGF-β-dependent liver fibrosis. Sci Immunol 2018;3:eaar7754.
[35] O'Brien KM, Allen KM, Rockwell CE, et al. IL-17A synergistically enhances bile acid-induced inflammation during obstructive cholestasis. Am J Pathol 2013;183:1498–1507.
[36] Zimmer CL, von Seth E, Buggert M, et al. A biliary immune landscape map of primary sclerosing cholangitis reveals a dominant network of neutrophils and tissue-resident T cells. Sci Transl Med 2021;13:eabb3107.
[37] Zaki NHM, Shiota J, Calder AN, et al. C-X-C motif chemokine ligand 1 induced by Hedgehog signaling promotes mouse extrahepatic bile duct repair after acute injury, 76; 2022. p. 936–950.
[38] **Liliang J**, **Batra S**, Douda DN, et al. CXCL1 contributes to host defense in polymicrobial sepsis via modulating T cell and neutrophil functions. J Immunol 2014;193:3549.
[39] Ma K, Yang L, Shen R, et al. Th17 cells regulate the production of CXCL1 in breast cancer. Int Immunopharmacol 2018;56:320–329.
[40] Choudhury SR, Babes L, Rahn JJ, et al. Dipeptidase-1 is an adhesion receptor for neutrophil recruitment in lungs and liver. Cell 2019;178:1205. 21.e17.
[41] Wertheim-Tysarowska K, Oracz G, Rygiel AM. Genetic risk factors in early-onset nonalcoholic chronic pancreatitis: an update. Genes (Basel) 2021; 12:785.
[42] Qiu L, Gong G, Wu W, et al. A novel prognostic signature for idiopathic pulmonary fibrosis based on five-immune-related genes. Ann Transl Med 2021;9:1570.
[43] **Marshall A**, **Lukk M**, Kutter C, et al. Global gene expression profiling reveals SPINK1 as a potential hepatocellular carcinoma marker. PLoS One 2013;8:e59459.
[44] Zhang D, Zhang Y, Sun B. The molecular mechanisms of liver fibrosis and its potential therapy in application. Int J Mol Sci 2022;23:12572.
[45] Gupta V, Gupta I, Park J, et al. Hedgehog signaling demarcates a niche of fibrogenic peribiliary mesenchymal cells. Gastroenterology 2020;159: 624–638.
[46] Pinzani M, Luong TV. Pathogenesis of biliary fibrosis. Biochim Biophys Acta Mol Bas Dis 2018;1864:1279–1283.

Keywords: Primary sclerosing cholangitis; Biliary fibrosis; Bulk RNA-sequencing; Liver transcriptome.

Received 9 March 2024; received in revised form 29 October 2024; accepted 3 November 2024; Available online 12 November 2024

Journal of Hepatology, Volume *7*

Supplemental information

Liver transcriptome analysis reveals PSC-attributed gene set associated with fibrosis progression

Alena Laschtowitz, Eric L. Lindberg, Anna-Maria Liebhoff, Laura Anne Liebig, Christian Casar, Silja Steinmann, Adrien Guillot, Jun Xu, Dorothee Schwinge, Michael Trauner, Ansgar Wilhelm Lohse, Stefan Bonn, Norbert Hübner, and Christoph Schramm

Liver transcriptome analysis reveals PSC-attributed gene set associated with fibrosis progression

Alena Laschtowitz, Eric L. Lindberg, Anna-Maria Liebhoff, Laura Anne Liebig, Christian Casar, Silja Steinmann, Adrien Guillot, Jun Xu, Dorothee Schwinge, Michael Trauner, Ansgar Wilhelm Lohse, Stefan Bonn, Norbert Hübner, Christoph Schramm

Table of contents

Supplementary Figures……………………………………………………………..…2

Supplementary Tables……………………………………………………………………7

Supplementary Figures

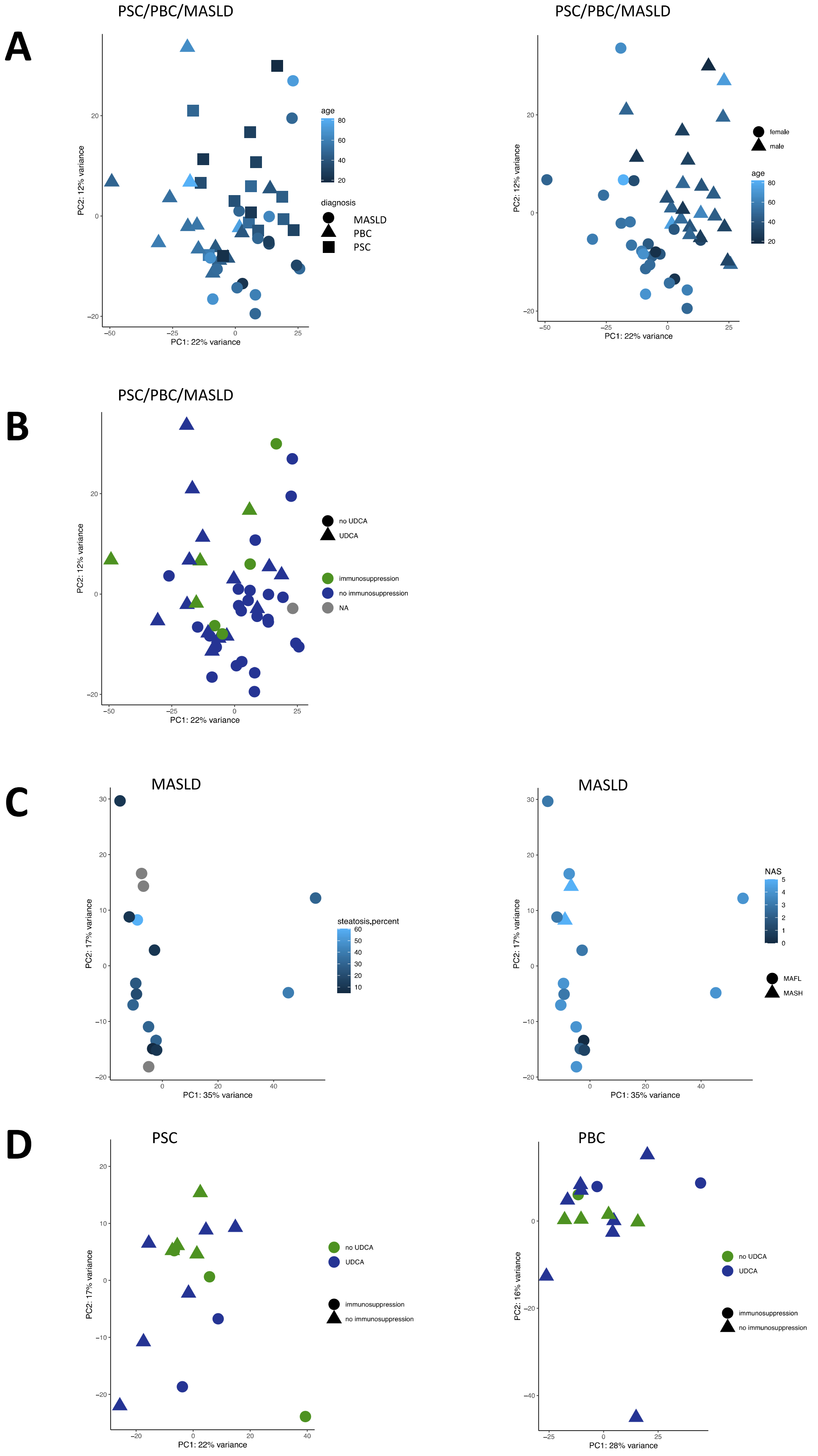

Fig. S1. Principal component analysis between samples of people with PSC, PBC and MASLD after RNA-sequencing analysis. (A) Principal component analysis of liver samples of people with PSC (n=16), PBC (n=15) and MASLD (n=16) regarding age or sex. (B) Principal component analysis after RNA-sequencing of liver samples of people with PSC (n=16), PBC (n=15) and MASLD (n=16) advanced or early fibrosis and immunosuppression or UDCA therapy at time of liver biopsy. (C) Principal component analysis of liver samples of people with MASLD regarding percentage of steatosis and NAFLD activity score. (D) Principal component analysis of liver samples of people with PSC or PBC regarding immunosuppression or UDCA therapy at time of liver biopsy. Counts of the filtered genes are used. MASLD, metabolic dysfunction-associated steatotic liver disease; PBC, primary biliary cholangitis; PC, principal component; PSC, primary sclerosing cholangitis.

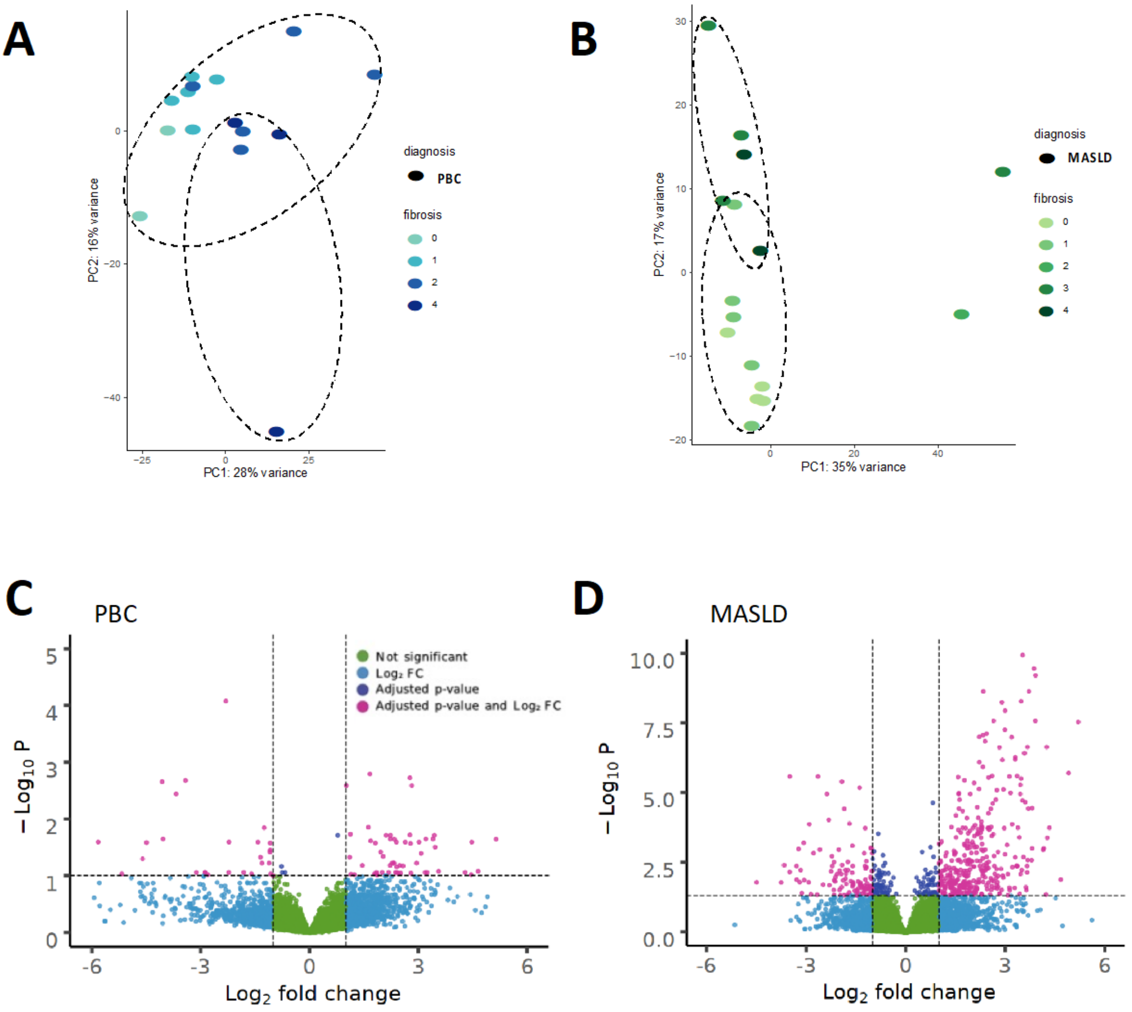

Fig. S2. Principal component analysis between samples of people with PBC and MASLD after RNA-sequencing analysis. (A)(B) Principal component analysis of liver samples of people with PBC (n=15) or MASLD (n=16) subdivided into advanced and early fibrosis. (C)(D) Volcano plot illustrating genes from the filtered data set, that show significant differential expression between advanced and early fibrosis in 15 PBC or 16 MASLD patients. Corrected counts of the filtered genes are used. MASLD, metabolic dysfunction-associated steatotic liver disease; PC, principal component; PBC, primary biliary cholangitis.

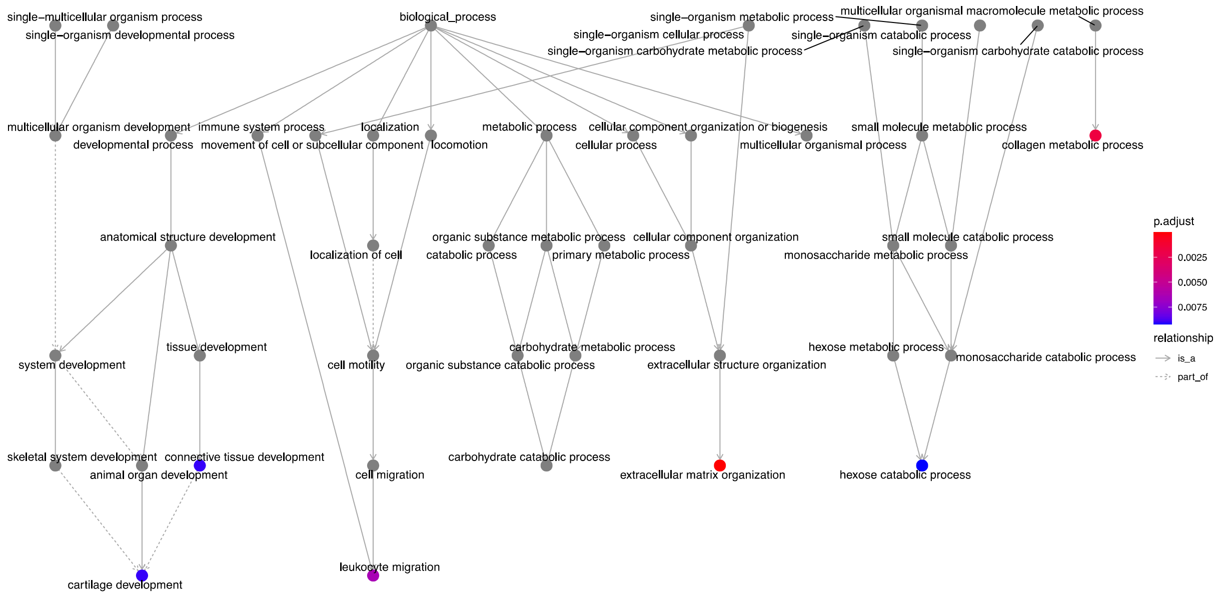

Fig. S3. Gene ontology term enrichment analysis as net plot for the biological processes of the 367 differentially expressed genes between advanced and early fibrosis stage in people with PSC. PSC, primary sclerosing cholangitis.

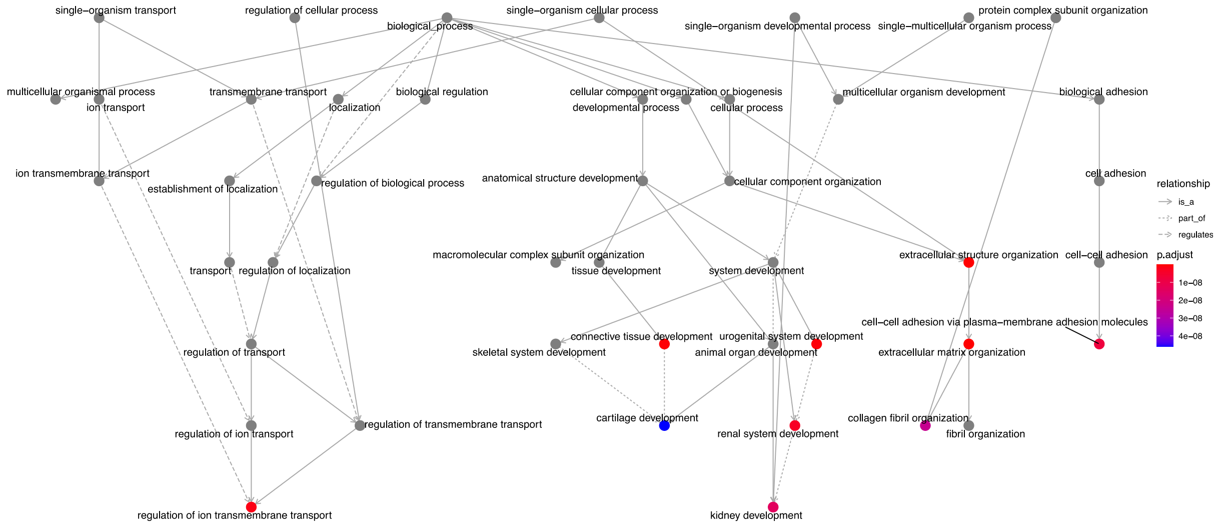

Fig. S4. Gene ontology term enrichment analysis as net plot for the biological processes of the 1351 differentially expressed genes between advanced and early fibrosis stage in people with PSC of the external cohort. PSC, primary sclerosing cholangitis.

	ID	Description	GeneRatio	BgRatio	pvalue	p.adjust	qvalue	geneID
**GO:0030198**	GO:0030198	extracellular matrix organization	24/134	368/18670	1.576242e-16	3.585950e-13	3.137551e-13	MMP2/COL22A1/COL9A2/COL1A1/FBLN1/SPP1/LAMC3/COL13A1/MMP24/COL4A2/LUM/MFAP4/FAP/TTR/CCDC80/COMP/AEBP1/MFAP2/COL16A1/COL4A1/COL5A1/COL8A2/COL1A2/FBLN5
**GO:0043062**	GO:0043062	extracellular structure organization	24/134	422/18670	3.411246e-15	3.880293e-12	3.395088e-12	MMP2/COL22A1/COL9A2/COL1A1/FBLN1/SPP1/LAMC3/COL13A1/MMP24/COL4A2/LUM/MFAP4/FAP/TTR/CCDC80/COMP/AEBP1/MFAP2/COL16A1/COL4A1/COL5A1/COL8A2/COL1A2/FBLN5
**GO:0001101**	GO:0001101	response to acid chemical	14/134	343/18670	1.794024e-07	1.360468e-04	1.190351e-04	MMP2/NFATC4/COL1A1/CCL19/BCHE/PTGES/FZD7/PTK7/OSR1/DGAT2/COL16A1/COL4A1/GLRB/COL1A2
**GO:0032963**	GO:0032963	collagen metabolic process	8/134	115/18670	1.772212e-06	1.007946e-03	8.819088e-04	MMP2/COL1A1/COL13A1/MMP24/MFAP4/FAP/COL5A1/COL1A2
**GO:0030199**	GO:0030199	collagen fibril organization	6/134	54/18670	2.378043e-06	1.031308e-03	9.023500e-04	COL1A1/LUM/COMP/AEBP1/COL5A1/COL1A2
**GO:0071229**	GO:0071229	cellular response to acid chemical	10/134	209/18670	2.719934e-06	1.031308e-03	9.023500e-04	MMP2/NFATC4/COL1A1/FZD7/PTK7/OSR1/DGAT2/COL16A1/COL4A1/COL1A2
**GO:0001503**	GO:0001503	ossification	13/134	398/18670	5.935879e-06	1.929161e-03	1.687932e-03	MMP2/PDLIM7/COL1A1/ROR2/SPP1/COL13A1/OSR1/MDK/COMP/GLI2/TMEM119/CTHRC1/COL1A2
**GO:0090596**	GO:0090596	sensory organ morphogenesis	10/134	256/18670	1.620625e-05	4.608653e-03	4.032372e-03	ROR2/FZD2/PTK7/EFEMP1/OSR1/MFAP2/GLI2/COL5A1/COL8A2/CTHRC1
**GO:0090103**	GO:0090103	cochlea morphogenesis	4/134	24/18670	2.411527e-05	6.095805e-03	5.333565e-03	FZD2/PTK7/GLI2/CTHRC1
**GO:0061448**	GO:0061448	connective tissue development	10/134	273/18670	2.812836e-05	6.399202e-03	5.599024e-03	COL1A1/ROR2/EFEMP1/LUM/OSR1/MDK/COMP/DGAT2/GLI2/COL5A1
**GO:0038063**	GO:0038063	collagen-activated tyrosine kinase receptor signaling pathway	3/134	10/18670	4.181388e-05	8.634641e-03	7.554936e-03	COL1A1/COL4A2/COL4A1
**GO:1903034**	GO:1903034	regulation of response to wounding	8/134	179/18670	4.554536e-05	8.634641e-03	7.554936e-03	PLAU/ANXA1/DUOX2/SPP1/FAP/PLAT/MDK/F3

Fig. S5. Gene ontology term enrichment analysis for the biological processes of the 150 differentially expressed genes between advanced and early fibrosis stage in people with PSC shared between people with PSC of our cohort and the external cohort. PSC, primary sclerosing cholangitis.

Supplementary Tables

Table S1. Differentially expressed genes after transcriptome analysis of liver biopsies from people with PSC with advanced versus early fibrosis. PSC, primary sclerosing cholangitis.

Gene	baseMean	log2FoldChange	padj
STPG1	66.5165213538814	1.03806382351779	0.0393619361208439
ITGA3	140.491643733775	1.29857840186187	0.0497488322542989
TMEM132A	58.8531565075408	2.04822978284	0.000241749779458621
MRC2	201.499198878481	1.15714907686606	0.0223032780992596
SYT7	2718.81624131427	-1.54886236133141	0.0152202092748826
PLAUR	38.5005642382046	1.26846629544632	0.0448373122614124
CLDN11	46.5668438654263	1.38483036426007	0.0244661722922571
CD6	55.9394183328679	1.10582450276233	0.0454614128411657
MTMR11	160.061699393749	2.37529140317237	0.000622502352025009
DPEP1	14.1874676617504	4.53579846520858	0.00458597922761639
IGF1	284.184593248002	-1.38758429731856	0.0402827850730024
VSIG2	88.9899453940755	2.4085011273085	4.30620795424635e-07
CD44	331.060984721737	1.36304811668883	0.00832512715352382
VCAN	111.491518490657	1.7665747549149	0.00850299609096921
USH2A	642.062708940655	-2.06940530406681	0.0116977238198055
ADAM28	42.8711096667586	1.58204477318708	0.0223032780992596
ARHGAP6	17.033732061928	1.40338184588059	0.0423505751194941
COL9A2	48.5289025979516	1.66437334892593	0.0128526411700704
LAMC3	144.745726033502	1.49783903480821	0.0323837981911038
FNIP2	1719.58088972418	-1.19018694637878	0.026534248976213
KCNQ1	53.5491142437102	1.35020119543227	0.0236623901567946
LAMC2	90.4082754003076	2.30673832792489	0.0233052590259399
YBX3	288.083341359193	1.05674858262818	0.026534248976213
ARSF	19.7069563651464	-2.78070088724248	0.0419993266919554
DGAT2	4579.81835160077	-1.20355082270454	0.0118928185999902
WISP2	30.7994260907607	2.29913315517945	0.0124161205408763
LPAR2	44.2142094058927	1.15420215291291	0.00655017618272789
OAT	1542.90593136032	-1.73903592573659	0.0276057703816685
CTNNA2	3.38951359877144	3.22412470987315	0.00396134687371227
PFKP	78.7534631163276	1.82304472366081	2.73654366503025e-05
PKM	693.447007056561	1.33941128472522	0.000470120997494607
DAPP1	25.7274906495985	1.28253769271701	0.0175657976800143
ASNS	77.5022963583056	1.41206549161739	0.0223032780992596
OSBPL3	60.9879587359552	1.295023216471	0.0366396814332282
LMCD1	117.828328303571	1.10178352942537	0.00207449662846941
GLI2	12.813974460162	1.6323629734446	0.0139433820757551
CA12	10.6051434031515	3.27174005204218	0.000281632096946323
RAP1GAP	496.725092147025	1.17529142663532	0.00170912887430793
FBLN1	206.813345227329	1.89761903682911	0.0124161205408763
FAP	14.0094081524046	2.77286165378191	0.00196670586334776
EDN1	57.0334020260579	1.33785526203861	8.73623709976284e-05
ITM2A	64.0061628743081	1.05390351386666	0.00136385399469593
LXN	46.618577607664	1.83555946991572	0.000700004968033066

MOXD1	45.0161043351725	2.41359941569679	5.55289415179156e-06
KCNN2	424.698370841547	-2.01954267953284	0.00563430533042114
DLG3	66.6095273696504	1.44468503699146	0.00456537744305191
COL16A1	132.807722815464	1.59894824176693	0.00386805578593389
IGSF9	485.407518999226	-1.65640195214071	0.0469638052227516
MMP2	310.375159692147	1.90588143224301	0.000872105410600536
FER1L4	16.9305112799082	1.44410382968871	0.0116556854823266
CPXM1	22.8505870528033	3.28378559007582	0.00114922710855477
TESC	61.8897802177956	1.69641918141901	0.0216007986056562
RGS1	19.3032177943152	1.43922389440659	0.0312217335423497
LYZ	933.023902413363	2.20065322302666	0.0127164446852048
NRCAM	30.0215567027906	2.85419294999241	0.00255275528777046
CCDC80	219.172506102333	2.04340547132688	0.000122016416905653
SMIM24	131.884272141246	3.34116497831848	0.0464727379851905
CRISP3	24.4015525322626	6.62424094672966	0.0359249832823872
TSPAN15	102.867531064846	1.23053762456644	0.00887682008157572
IGFALS	4997.54953887433	-1.89267883698521	2.49628243675649e-05
PIK3IP1	237.208003950012	1.03593157916488	7.66651259636765e-06
NFATC4	47.0681103360513	1.21615054928142	0.00232961698404084
EEF1A2	25.0220577236347	2.77692740926409	0.0453078967432116
FERMT1	9.88125984725863	1.48383889899852	0.0359249832823872
TLDC2	13.2741756492937	1.05369221904476	0.0462302862139009
NOL4	35.411650139348	-2.87020295233635	0.00124715670210108
PCSK1N	8.65655188989601	2.95834054224657	0.026534248976213
RGCC	26.9681730957459	1.92169546999318	0.014091852792574
TMC5	16.6940575233898	3.91808503345781	4.76206911894816e-05
IGDCC4	4.27321679994116	2.01206320183103	0.0129620692856621
PLAT	106.02582100555	1.24663586365919	0.0280786953008869
STMN2	14.0786133176183	3.40677652680793	0.000220054460930348
CLIP3	45.3149597818347	1.04465022155672	0.00155038885980495
ARRDC2	626.922737043663	1.41993997850275	0.00409024411769958
COMP	5.47313706560234	4.02993243642682	0.00239496580864581
SFRP4	15.3926277606175	2.4703086719809	0.000621270964562682
AEBP1	715.195852021125	1.53963858681242	0.00143355822134606
PTGDS	293.957087897795	2.05676291062277	0.000565400801631715
PPIF	3795.30589961834	-1.09503825028898	0.0216007986056562
GALK1	2836.11711193471	-1.17741419041636	0.0019225525183266
COL1A1	2361.88701912657	1.92255423403243	0.00569899792401553
MAP2K6	73.3327549659799	-1.39905889608863	0.044351658295654
PMP22	111.563527042939	1.2039800779637	0.014091852792574
CWH43	23.9304651301334	-1.56867433665581	0.04953582263368
GALNT7	29.8264411292846	1.11072391848587	0.0441055801676318
SOD3	91.3403125490206	1.46817637275783	0.044126307893406
GLRB	18.9041988420625	1.15458891506574	0.026534248976213
MDK	220.811932318709	1.66361320069601	0.00259562456054368
TBC1D30	18.0123796792122	2.37224404460533	0.0044913935293927
SLC16A10	293.289269506357	-1.40683231321362	0.0197471732766389
UNC93A	464.698004500009	-1.58929657515207	0.0129620692856621
MDFI	9.36919717807602	2.05644034080846	0.0245094851186537
PTK7	57.206783266752	1.09529549571709	0.0292106580113676
ENPP5	15.5638924274041	1.56372260485098	0.0411787160015384
C7	2603.77129834282	1.74983570549005	0.0018641315604665
GHR	5859.34470462983	-1.55106760768546	0.00514768431930594
DBN1	105.158815936901	1.65470903806304	0.00413244111925815

BCHE	3762.93786994956	-1.40382893150822	0.011117824620847
FGF12	13.1021743655572	1.41257542934114	0.0193437469589119
NEK11	30.9926466200761	1.1914757404014	0.000104146973540915
CISH	1050.25228895465	-2.56120238877464	0.0215700168340007
SLC4A3	12.9616067457824	1.8665617921188	0.0116977238198055
EFEMP1	266.577682527606	1.85814206512092	0.0116977238198055
CHST10	18.2051652868485	1.24475106758354	0.0428027953217387
QPCT	15.5066106597818	1.39381163842951	0.0134697203002485
PRG4	5065.54300009144	-1.05747138969852	0.0276257871124938
HAO2	7763.23881799858	-1.22691383617119	0.0161286483441998
RP11-268J15.5	13.9704884519003	1.40076617425078	0.0314440873350202
MFAP2	13.7747283707775	1.71972871655818	0.00625306630855344
RGS4	70.8948472067232	2.75547886933132	0.00559623275327865
F3	42.5794994811133	1.4094776218433	0.0253939773034529
A4GNT	9.01865779661433	3.91332881673388	0.00312573979164123
TREH	239.988855987171	-1.43553657919268	0.04953582263368
ZNF541	28.0093040514298	3.5392242888385	3.72456080498509e-05
TTR	177951.395480882	-1.23339379546909	0.0263661920557645
SPP1	1002.33839479807	2.29100358230446	0.0248623769337723
RARRES1	75.6928191718011	1.29701560483059	0.0419554492732191
CCND2	54.4660222420215	1.11515217484185	0.0167436813588506
LTBP2	162.24751375372	1.35234748023678	0.00443546492524697
DPPA4	20.964591204683	-1.94930101092816	0.00447317475911574
AKR1D1	7285.48703837096	-1.17883981481345	0.0250487557014472
PLAU	31.1890084134891	1.57831766407755	0.00665099636686889
IFT81	161.824156831574	1.01940290967059	0.0464727379851905
SSPN	18.7625624107862	1.6550446710053	0.0007133067010293
MMP19	48.1576951100384	1.52136834851111	0.0352941284316339
GPR83	5.29894658763602	1.83527490707152	0.0134697203002485
TTPAL	1559.93193303981	-1.04649863840155	0.0111238475280754
F13A1	37.3845216682584	1.69410906336621	0.0490500927582505
MMP24	26.6134893645728	1.30925215537635	0.0279165254472489
RASL11B	9.80531627061051	1.48980951893679	0.0303774718323401
POM121L9P	8.23075873377773	1.68883931165024	0.0366504396261426
KRT17	4.1879472265049	3.24078277070588	0.00255275528777046
SPECC1	32.2214990752708	1.1501215147502	0.000230976966558127
PDE11A	205.991407444703	-1.78091178710208	0.0213715871599184
ISLR	133.947539006317	1.28078816171815	0.0234331789451119
LOXL1	26.5865931190786	2.22536880442203	0.000872105410600536
BBOX1	1067.77498448175	-2.19570426853496	0.0143909455332959
LRRC6	23.8499809346267	1.01669676711197	0.00832512715352382
EGLN3	30.0410179970938	1.49085352273988	0.0279165254472489
PLVAP	205.760169453539	1.3568450342718	0.012682379528164
COL5A1	469.835515380134	1.41862474206778	0.0175657976800143
SLC6A8	31.0432112158602	1.31946115241595	0.00413244111925815
EDA2R	8.43876747674721	2.51641280405321	0.021399685810927
SLC6A6	75.870335964899	1.10090990800637	0.0448955648698778
PODNL1	4.98529719973018	1.79112892476592	0.043822453105594
SLC6A11	13.2339784647713	4.68529761462635	0.00011917986462994
JCHAIN	341.652956428773	2.00702300666729	0.0366396814332282
LOXL2	54.0912880337123	1.35308549582521	0.0176519096938576
HMGCS2	55408.2167670315	-1.06994114618196	0.0385384685924505
GRHL1	269.736226079776	-1.02397339875142	0.0390897962902708
RERG	31.6168831311096	1.22553763602753	0.0148363111542061

PIWIL4	36.5768311228057	1.86617330946368	0.00480963207387495
GGACT	187.151823243126	-1.02405763126228	0.0236623901567946
COL4A2	940.290427458998	1.26802054788983	0.00447317475911574
ANXA1	276.833916677907	1.18370856447084	0.0195864925309913
GOLM1	436.325474790801	1.49274826855451	0.012376804013246
TMEM243	44.7774246567758	1.01573874222658	0.00370180139910918
KRT87P	19.1549741317029	3.74677105597519	0.00447317475911574
KRT7	483.692143224011	2.14412865469576	0.00832512715352382
MICAL1	176.832679324196	1.05809325537701	0.0123507784191383
SLC9A5	3.9610819168653	1.79729794714746	0.0167070800306499
PCNX2	26.5065962182972	1.38679156923649	0.0113724977869291
VILL	48.2435682005507	1.07726714687503	0.014091852792574
THSD1	62.4214644431232	1.25151974113229	0.00814389363426249
NACAD	5.65326110754996	1.93153336368937	0.0138556565656711
CIB2	9.85011888977611	1.44640930610868	0.0417009252138584
ERMN	4.765738642636	1.66339992737847	0.0257592495901362
ALDOB	225490.608108383	-1.05672411873587	0.012378751560942
LRRC1	30.138605731697	2.08301397829693	1.21991855719815e-05
INHBE	2200.5448790829	-1.36604990600663	0.0490500927582505
LUM	645.922765518587	2.11377593436215	4.44549390857997e-05
FBLN5	366.176609579993	1.20372563360628	0.0257525918285535
DUOX2	137.737673843907	5.22551015302095	0.000233184730366442
FGF7	17.6938992615225	1.7743405054519	0.0022406734881307
MFGE8	229.912036723457	1.04461492188836	0.00708217259838561
GAS8	48.9946681653246	1.13118418863	0.0226308476254405
ZNF287	14.2040673739995	1.19255683046802	0.0225493448675838
ZMYND15	98.5300457083051	1.04740691943132	0.00832512715352382
SLC16A3	64.3129444700439	1.14641290162475	0.0267058371528344
FKBP10	170.002814715017	1.11472748150227	0.0118928185999902
VAV1	81.1352743029951	1.14851090642461	0.0439505299787088
COL6A2	2085.43151674754	1.05945370680476	0.0166337990848121
DNMT3L	33.011401530058	-2.43815502912347	0.0152982569172098
NTN5	7.8183396847862	1.65437060770287	0.0496868612625693
CTSK	159.40169667824	1.45056832776778	0.000123133277169609
PKLR	3256.35190480623	-1.25689609153314	0.028406839548112
OSR1	5.86629808638739	2.19393601765212	0.0152982569172098
BOC	15.3863556976769	2.04804968942634	0.000640579230835193
NCEH1	106.130482959207	1.01838463558949	0.0267058371528344
PCYOX1L	40.5801900271608	1.29993201537342	0.00447317475911574
N4BP3	9.06476519982771	1.53468244147493	0.0245094851186537
TPBG	29.9850078850926	1.32224909232844	0.00232335290843295
CXorf36	38.3443045272058	1.52340140640574	0.00358022542935344
GPC3	41.4989038436535	1.70598637832766	0.0262522606393077
CDKN2B	24.8376772001254	1.37140262073518	0.0368585835932663
PTGES	6.27469562221392	1.76046390273486	0.0352941284316339
SYT8	4.28282966245027	2.83128282943831	0.0135313281167945
ST14	587.960687558563	1.27593332239402	0.0285236322931399
TKFC	5366.13665613029	-1.08797939454806	0.0279165254472489
CNKSR2	44.2287517211804	-1.38363932505889	0.00458597922761639
LYPD1	2.20175723069016	2.75365073457383	0.0366396814332282
CCDC102B	25.9497295214434	1.05746723474594	0.0027253176849524
NOCT	100.082615238869	-1.367072513128	0.039851792529239
DPYSL4	5.26588812612898	3.42890834545507	0.00555094083037749
BICD1	22.5143423608878	1.1512958015596	0.0233052590259399

KCNE4	12.3272669303689	1.87696472279711	0.00307062914760934
DNAAF1	17.7360429672713	3.57742954004175	0.00315596331648237
ENPP3	133.006551044748	-1.06441017688131	0.0387908255640911
UCHL1	13.4192076518939	1.86758480351612	0.0403095994490255
CNTNAP3B	16.6091410821205	2.01122459618942	0.0129620692856621
TMEM55A	23.5645711951517	1.11050559845694	0.0195864925309913
TTN	6.38663401302457	1.89194552134127	0.0462253842821979
KDM8	1527.31960111126	-1.22871186808506	0.0267058371528344
FZD7	38.0236029373411	1.14178134209496	0.000591521357043573
ADCY8	4.69870414510546	2.78506807907168	0.0333653871285596
GLYATL2	3.92967031509806	4.24908328407629	0.0380156232830008
IL34	36.64854217118	1.31748393188525	0.00447317475911574
SLC30A2	6.60452970781876	3.39468760912824	1.4115576138242e-05
FANCC	1062.78660456142	-1.19360170618265	0.00114922710855477
MRAS	86.3560431101375	1.00543399356148	0.00529569835760759
FAM46B	8.86608717455505	1.84497313357988	0.0368585835932663
HK2	29.2218620661504	1.21601691197665	0.0352639480314662
ABR	215.541715129643	1.04950957723711	0.00344149508810554
UBASH3A	15.8059253623934	1.63552803295609	0.0389844835942515
GPSM1	105.351708456197	1.6475708989903	0.00686055532013383
PKN3	70.0367768352385	1.46772608201516	0.00413244111925815
BRSK1	5.14454628562609	1.55971030994676	0.0236623901567946
DMKN	47.864105182909	1.73271305385891	0.00194665288567357
ZG16B	28.0651841449293	2.57182186579996	0.0109943276702351
PDZK1IP1	149.960372848568	2.89120331579437	0.00012691068049179
NEXN	39.9467917674991	1.18943922214996	0.026534248976213
GBP2	552.661175782543	1.13537050405434	0.000871826265665489
FBXO41	29.2786595798882	1.08267717520427	0.0368585835932663
SLC16A14	57.30684770302	-1.69604872367469	0.0259968335312167
CFAP221	38.0658205658388	1.86782479813362	0.0213715871599184
MSX1	3.27343173969965	1.83757047446882	0.048291471857785
ELF3	378.618542241018	1.28381893412226	0.0214775095833773
CXCL1	43.0314288629911	2.61627544699973	0.00346378836813225
CPA3	22.3074716500728	1.56952413581876	0.0462308086170101
CDCP1	27.4316603665186	1.3701576968661	0.0352941284316339
MELTF	59.4807982425447	1.93449772091135	0.00239496580864581
MST1R	19.1880078607679	1.330345273414	0.0157651845071714
SPINK1	126.996618592167	4.76322621041791	0.00191116673853965
GPX8	34.8069291407154	1.30677455947349	0.0124161205408763
FOXQ1	18.3806187008834	1.71101329018328	0.00617008391111822
TMEM200A	7.76394771977101	2.10301759725424	0.0246358278611337
KIAA1324L	27.4609607204619	1.38082455109877	0.0221385087012464
COL1A2	1584.75397324539	1.65386485512243	0.0103344529882261
FNDC1	19.7483012589243	1.76284068389094	5.05822779988896e-05
CTHRC1	12.9179639498875	2.50391589210157	0.00132104546348734
INTS6L	58.6062911638115	1.08243753261654	0.00949989650352037
GPR176	45.6293251900392	1.29806257522318	0.0426249393078605
AMN	2353.31651169082	-1.71434931694145	0.0317486831648512
SPINT1	151.109810984131	1.74799346765198	0.00832512715352382
MFAP4	373.5431822839	1.37950067753002	0.0175657976800143
RRAD	32.5887544805902	1.62847152242031	0.0138556565656711
LDHC	12.6144920864277	1.79440553965628	0.026534248976213
MAP1A	11.5022877787756	1.58869927249401	0.0142626369324022
TTC16	4.57007182593728	1.59751961257696	0.0490423125533777

CERCAM	58.8769259762869	1.43512286801301	0.000978809314753162
PROCA1	20.7490923569601	1.0626840882965	0.00596399798120693
CACNB3	32.4711801056458	1.37982042352272	0.011117824620847
TUBA1A	323.053678525381	1.50309313950785	0.00156444875779015
TMC4	95.917559479241	2.17237155442249	0.0147784013974721
HID1	123.51895956888	1.10077416904571	0.000736517379479569
EVPL	7.0272274151282	2.06605666126713	0.0124161205408763
ABCA3	76.5568915121764	1.10199748562576	8.12005257606895e-08
VWCE	374.359918931648	-1.20414176470783	0.0307049369323104
MAP2K1	1231.39178268292	-1.10875569717594	0.0416571773360099
ROR2	8.44709734949091	1.96118407225615	0.026534248976213
COL22A1	7.98935112076064	2.92873039551097	0.000634718401513313
NRG4	27.7197494439662	-1.3057693403565	0.00261815967671356
ROBO1	103.165167259068	1.09514885431178	0.0345839313513714
ITGAM	84.5593193823196	1.21907486762045	0.0138556565656711
KRT86	10.5480684615895	3.18150050840981	0.000637186078544675
OSCAR	32.2365546907559	1.18665921215306	0.0490500927582505
PKIA	3.71229037322531	2.18776596055332	0.0351801870337779
ZNF439	23.5789460383552	1.0819369778326	0.0245094851186537
TMEM51	66.3334963328564	1.3098125020824	0.00617008391111822
COL8A2	19.3860381846532	1.48555894692282	0.011117824620847
ARNT2	69.9932217305949	1.37711856332682	0.0419848908500659
CCL19	131.817456666968	2.45637830314986	0.00850434798457618
MIR4435-2HG	45.8446638480062	1.49083961846192	0.00517958566696273
SULT1B1	463.54973421385	-1.49449197655218	0.0257592495901362
LRFN4	17.6767665302674	1.16862420605248	0.0158091077853298
MUC13	47.5057217788688	2.56761845646851	0.0440318479158445
GPR160	19.8392386650341	1.04720991189094	0.0393619361208439
CBX2	27.6182138301236	1.25393612462462	0.00832512715352382
MARCH3	19.8419977709723	1.28807683014489	0.0279469086210598
SLCO4C1	124.225326304498	-2.07747084364115	0.0318919830920021
SLC26A9	4.05257881428479	3.6380502652806	0.0174222085940857
MFSD4A	31.8269907829332	1.00612687798941	0.0336470517913728
SEZ6L2	54.2970279852555	2.46363608846924	0.00655017618272789
ASPHD1	13.275244652114	2.4233992277671	0.0351920346188881
ZG16	1525.38353122384	-1.33692878745728	0.0225493448675838
SLC22A1	24572.2468955797	-1.27835562637699	0.00653951956473021
CHST1	18.6128821541256	1.15073005108844	0.0245094851186537
SCUBE2	19.0899788725745	2.1364662921745	0.00617008391111822
MCTP1	33.4864843965165	1.07327825708491	0.00525535682332568
CLCF1	26.4288586119382	1.67104386855224	0.028386448030583
P2RY2	54.3184185810911	-1.30993261384497	0.0391541075129843
ETV4	38.8001697407004	1.96447811716801	0.000565400801631715
TUBB6	120.096168328372	1.06680821547597	0.026534248976213
JAKMIP2	62.6607816775768	-1.80569765638401	0.0257592495901362
SPHK1	53.5383521343724	1.41525754324914	0.0273912808807808
SLCO3A1	115.97625484726	1.10857999228953	0.00104025557066111
NCKAP5	105.881697215457	-1.19502722241914	0.0248896374757527
FUT2	10.4784795826099	2.53602998388858	0.014091852792574
ZDHHC13	40.016688075942	1.11305317841798	0.00832512715352382
MAMDC4	906.853315192298	-1.6823119416267	0.0339274385409484
CTXN1	3.94722254786508	2.68677642445604	0.0279165254472489
CA8	3.15255355192971	3.72472373916568	0.0325851172563534
MSC	24.1057362403481	1.51476318238163	0.0246358278611337

TMTC2	30.9656174341802	1.11235294584903	0.0393619361208439
B3GNT3	31.9215283640331	1.99026765298719	0.00686055532013383
FZD2	7.01321348520705	1.87065013065672	0.0329623078446972
BACE2	177.661615269918	1.39606898681741	0.0103479625143548
B4GALNT4	6.9841703370108	2.23592063081016	0.0263604600407009
SPACA6	12.1033208478301	1.31332386703112	0.0245094851186537
EPHB3	34.0385107082268	1.34280268182454	0.0497488322542989
ANXA2	2648.25880828215	1.2843018344508	0.0419848908500659
TMEM119	12.9777760642157	1.953616927646	0.0104071066557526
MEX3B	14.6837479329409	1.54385645837903	0.00194665288567357
PNMA3	21.6480975651754	1.16492097420367	0.0257592495901362
TACSTD2	147.706322342956	2.23732089041595	0.0236623901567946
PKP3	11.1336886150024	2.97117805865206	0.00655017618272789
PTP4A3	45.0097617522386	1.20690145086479	0.0111011461351316
ZNF93	9.6946617476483	1.61449473083614	0.0035883692724956
AHNAK2	19.6872328279866	1.18159873352536	0.0361018980635552
AGBL4	17.9279463194384	-2.19311324588356	0.0154973432397479
THBS2	221.656804958519	1.60125982627471	0.0138556565656711
DYNC2H1	58.5316728468527	1.25076765823113	0.0126567867962124
COL4A1	784.946739001199	1.13148336819659	0.0245094851186537
SAMD11	44.8336043778148	1.52481426425028	0.0114057928206436
IER5L	31.8348594385951	1.1768305920263	0.0490500927582505
C15orf52	178.395985215227	1.2863744379814	0.0373490476093378
BCL2L15	8.44444279113645	1.88138747727122	0.0477420191772099
SEMA4A	44.3688166546354	1.18566510241136	0.0095707105318286
MME	1262.26520403768	-2.05722836116042	0.00429997124754463
PDLIM7	147.063028902016	1.02539067813598	0.00443546492524697
ZNF763	25.7490621774644	1.3422029655819	0.0153852767581549
STMN3	52.1172710462064	1.20827346149613	0.000894519739610119
COL13A1	12.6653206123195	1.26212984191063	0.0497488322542989
ZNF677	20.4138086606504	1.13076848760694	0.0248890850462386
AKR1B10	400.634725848938	3.48831388059655	0.0266950007860527
ZNF607	32.472295758793	1.08006277479651	0.0104071066557526
CSF2RA	37.511121255304	1.88031821058377	0.00255275528777046
HLA-DRB5	939.974039884347	2.51436686212067	0.0221385087012464
STK39	38.7993350398025	1.45987802863234	0.00156444875779015
NEU4	1711.36286194206	-1.04814636459478	0.0273268100481388
HLA-DOA	159.768162036269	1.20151949472843	0.0428027953217387
PNMAL2	19.2384816106372	1.1527038108133	0.0298508690047339
TMEM231	54.2007906900034	1.05375752789696	0.0221385087012464
ADGRG1	90.8376098069352	1.38013166545624	0.00480963207387495
KRT81	7.01218251335316	4.19309646488982	0.000176191036074143
GYG2P1	25.4824257891795	-1.54455300946065	0.0373490476093378
IGLV6-57	46.9847950835676	1.74838601833091	0.0390713872916524
IGLV1-40	109.790807968524	2.13000776470995	0.0366504396261426
IGHA2	168.453029027125	1.8810679537933	0.0467701791836936
IGHV1-18	77.0921269189072	2.50198191067103	0.0193437469589119
IGHV3-33	34.1488167194649	2.04094070095642	0.0385384685924505
ACKR1	16.4494905504738	2.39929369816641	0.000468837537479265
SYCE3	6.62231851464434	1.89120165436044	0.0423885157123322
XKR9	63.221810046975	-1.75895370620339	0.0248623769337723
RP11-54O7.3	38.4670489958354	1.212333006838	0.00255275528777046
ASS1P2	139.356343063202	-3.25879891130639	0.000606008834802697
RP4-710M16.2	377.685975183447	-1.89093628564753	0.0408683638483783

LINC01057	11.9434237595697	1.67379454328478	0.000830722836932396
AC005077.7	15.8935025635437	-2.19936915210317	0.00569899792401553
CTAGE4	6.9845402671504	1.97493840867418	0.0312233595258778
AC091729.8	18.1904075427724	-5.40074854243956	0.00555094083037749
RP11-397G17.1	161.265839569125	-1.75001707657808	0.000550392833555507
LTB	81.201449302388	1.2641119588369	0.0365566623073179
RP11-492E3.2	3.31567310007607	3.88820587690576	0.0207421187354651
MELTF-AS1	6.29607869034823	1.64723833923156	0.0428027953217387
AC013275.2	8.6347456113208	1.94374345377122	0.0463753909679883
LINC01160	5.83124726497043	1.46787437896554	0.0373661105988245
IGHV4-31	25.9127037962404	2.22854671282826	0.0385384685924505
AP006285.7	91.1463785551054	-1.77061389543315	0.0311356973335343
AC004862.6	274.955228135826	-1.18983523889114	0.0462253842821979
LINC00665	23.7424398004553	1.16035252983897	0.0393619361208439
LINC00342	96.7345620904456	1.45854522017873	0.0126567867962124
C12orf75	46.3501322002751	1.55678234353631	0.00368141964900117
AP006216.5	61.2987085992623	-1.41880803273268	0.0334309846472487
LINC01554	1469.62489894127	-2.90967576052876	0.000634718401513313
RP11-753B14.1	15.3823216491403	-3.81492718476513	8.49527863011772e-05
LINC01625	46.6499227186037	-1.95537515048314	0.0437807765068879
IGKV1-6	30.6727222466806	2.338417725677	0.0466454391619011
IGKV1-16	36.6019505238674	2.18361380286525	0.0351801870337779
HLA-DOB	13.5432673919713	1.75422374281124	0.0248623769337723
TDGF1	18.0333906516952	1.3637355891971	0.0236623901567946
FLJ22763	227.926272494158	-1.1745914621208	0.00832512715352382
IGKV3-11	257.252602830223	2.26611379680037	0.0236623901567946
IGKV1-9	81.1075716190529	2.27743980366678	0.0437484215050008
MBL1P	144.033776301417	1.16315731735514	0.0408683638483783
PALM2	35.8453041514964	-1.46243020511586	0.0309368216268838
DRAIC	126.5794226653	-1.6461141172713	0.0066822537620625
RP11-422N16.3	107.865065453742	-2.93641016038175	0.0215700168340007
TNXA	29.8064392060392	1.97603793667219	0.0257592495901362
RP11-434D9.1	371.982439134753	-2.1671590269302	0.0428501587391136
RP11-685F15.1	13.922378964398	-2.19114118651442	0.0276257871124938
RP11-397E7.4	4.03977592303764	1.70605807556213	0.0490500927582505
CTC-558O2.1	28.6611331246595	-2.3652456074241	0.0279165254472489
RP13-870H17.3	9.53157620191968	2.82773369414234	0.00919786347198352
RP11-535A19.2	7.45630230310244	-2.62373823583742	0.0262522606393077
RP11-44N21.1	5.6153617003144	2.0098625949151	0.0022406734881307
RP3-416H24.1	12.0632345368413	2.4618790041799	0.0161286483441998
CTC-378H22.2	2.35088565420691	2.32285651085474	0.0425472231211591
RP11-753A21.1	8.45782266226598	-2.02501087008574	0.0490500927582505
RP11-1069G10.2	6.40563668438189	2.77851325882879	0.049458085377701
RP11-800A3.4	67.9821817508672	-1.29992644941092	0.0490500927582505
TMEM178B	4.52277493902139	1.89701343950311	0.0345839313513714
CTD-2529O21.2	23.2053857386813	-1.72344695027542	0.0462302862139009
LA16c-325D7.2	2.98095252463193	2.52308268494715	0.011117824620847
LRRC37A7P	82.9656953766242	-1.41903091311697	0.0248623769337723
CTD-2132N18.2	19.5783247846369	1.02576715436324	0.0257525918285535
SMUG1P1	130.068198153431	-1.45979904920527	0.0271607660138017
MIR24-2	25.6306952125367	1.01896209306957	0.0447251356454561
MILR1	24.8544695455967	1.33518109256162	0.0225148566806415
CD24	665.40107612331	1.75887878828265	0.011117824620847
XXbac-BPGBPG55C20.1	4.16929469281986	2.54941331228089	0.0245094851186537

ADIRF-AS1	13.175817208874	2.28107770934571	0.0167436813588506
RP11-631N16.4	14.8057888706164	1.10975409793905	0.0438528821398412
SGK223	71.7149796552796	1.38321183999589	0.014091852792574
CCL18	41.4764287511523	2.57443683194739	0.0128526411700704
CNTNAP3P2	4.10394818822346	2.25175055382107	0.00617008391111822
RAB7B	22.6023000456575	1.7029270336662	0.0007133067010293
AC233755.2	13.463726285018	5.24570880996466	0.0175657976800143
RP11-39H3.2	82.8210483406775	-1.80336243027007	0.0082079776748903
RP11-329N15.3	13.9882548990521	1.31502956739264	0.0439505299787088
RP11-40A7.2	58.1593611699522	-1.33818350704963	0.0462253842821979

Table S2. Differentially expressed genes after transcriptome analysis of liver biopsies from people with PBC with advanced versus early fibrosis. PBC, primary biliary cholangitis.

Gene	baseMean	log2FoldChange	padj
SEMA3B	106.252673745257	1.01792631305576	0.00299320894590054
ARG2	32.0729270466563	3.20777520349771	0.0222148662092035
SLC22A17	13.7403940201114	1.68833664617511	0.0234164759451462
B3GAT1	330.785632228951	-4.06290649959507	0.0021060449852488
VIPR1	575.717882931384	-1.08356171629421	0.0389246839940229
FBXO2	845.560539466882	2.03335159840117	0.0464395152480275
SLITRK3	141.796227599134	-7.54955745924069	0.020980880193268
LRRC61	432.346917701521	1.12733983229235	0.0197207464992929
GDF15	196.926422673467	2.7718079466055	0.0197207464992929
RAMP1	2407.52480009748	2.39915590002154	0.0263117180533465
SPG20	299.961313984638	-1.0868660759023	0.0418273408769321
GSTM1	1582.75926057157	9.87650162240374	5.50522552777621e-63
SLC44A3	268.581554387947	1.95878251671691	0.0389246839940229
FAHD2B	93.4973695611101	1.81902185279525	0.0272233175025397
ALDH1L1	18991.0908116763	-1.24856661210005	0.0163438287425528
KAAG1	3.9127703136235	4.47240667553635	0.0234164759451462
POMZP3	57.3373983706961	2.11335071041715	0.020980880193268
ST14	537.75367632175	1.66834506720528	0.00164111199165595
ADGRA3	2911.92614174655	-1.4201106423066	0.0268043877341672
SLC5A11	22.4309546667405	2.33753037361579	0.0222148662092035
DMKN	75.3862580409731	2.2323493083895	0.020980880193268
COX7A1	163.159863296235	3.40787881125903	0.0222148662092035
SPINK1	482.533821635518	5.14793339101465	0.023214474538679
CMYA5	272.527747876613	-1.08305755179008	0.0291244846392159
LARP6	88.7326086555368	2.22629880634019	0.0222148662092035
FABP4	60.7504922995816	2.82329635806906	0.00280602875809337
HSD17B13	9158.00730163744	-2.30217962782736	9.10407076759242e-05
TMC7	15.1572375186213	1.62354104838993	0.0144297275592029
UGT2A1	85.8256205688496	-4.03488888125007	0.0222148662092035
MUC13	73.6972753508615	3.46238039206834	0.0298076303153813
SLC6A19	35.1362617579552	-5.82473646587567	0.0234164759451462
ASPHD1	25.9078522249864	3.44364123907278	0.0202044571838998
SCUBE2	33.1664438125838	2.64246704344229	0.0234164759451462
FAM9B	96.3013702358622	-3.67503944350075	0.00346566614437743
PCDH9	63.0913127052523	-2.22133963532328	0.0234164759451462
CIDEC	31.8597573790422	3.02180518001379	0.0234164759451462
DKFZp779M0652	153.39051284766	2.78732121501196	0.0272233175025397
HRAT92	10.6580372010041	2.77106236476161	0.00164111199165595
CES1P1	435.508701204926	-4.49100042465018	0.0268043877341672
CYP2B7P	1288.72065215491	-3.41646189956983	0.00204099697165222
CTD-2325P2.4	26.0616631966413	3.07767230723641	0.0389246839940229

Table S3. Differentially expressed genes after transcriptome analysis of liver biopsies from people with MASLD with advanced versus early fibrosis. MASLD, metabolic dysfunction-associated steatotic liver disease.

Gene	baseMean	log2FoldChange	padj
CFTR	273.956395227161	2.94378755232819	7.72809766076411e-06
DBNDD1	119.513371036829	1.39286767360068	0.002730647434486
CD38	44.6779935254418	-1.08190358047328	0.026588573140817
PRSS22	18.917215775178	2.91495841633327	6.76233427497206e-07
ITGA3	110.705723528929	1.56911638433046	0.00750288892446449
TMEM132A	29.5774447647859	1.97638240705748	0.00281405758548834
TNFRSF12A	128.597475726922	1.80452711873048	0.0098742846633194
USH1C	28.8605399812367	2.47616347759235	0.000190430625450584
PROM1	90.9543797449458	2.09447965369921	0.000147723553602656
SEMA3G	72.5929765591515	1.61336344825135	0.0233231940731534
EHD3	392.515678810604	-1.38956761307103	6.66860366658118e-06
MTMR11	89.7292961554225	1.36138756357488	0.0188508661606287
IL20RA	14.271065138535	1.74693439392454	0.0264252006666996
VSIG2	67.5656688691542	2.39115248663795	1.45553036620174e-07
SYT13	24.3774284668038	3.16271905903779	1.02685554441465e-05
PLEKHB1	40.7556629833221	1.91941844785035	0.000691768336020804
GRAMD1B	99.6374210556334	2.27221871854293	0.000416531132867255
ARNTL2	65.578509903385	1.32677204922387	0.017582772565213
SH3YL1	318.301668078193	1.02123995151687	0.00720883168484891
DEPDC1B	14.6887984754936	1.75537635227251	0.0250617882312635
CASR	3.83873371630235	2.72795124018841	0.0117542840360053
VCAN	93.678704067049	1.58860626496817	0.0459168154350462
FOXC1	8.30892123154557	1.51176842617186	0.00757856413671407
MCOLN3	11.9356672802958	1.87865193232783	0.0250617882312635
LAMC2	75.1860785012479	3.30091109498489	6.48338826169106e-07
SLC12A2	201.348120348318	2.0496162392261	0.00075136325920484
EYA2	24.232463636465	1.52834439324295	0.0323289214077222
FGFR2	1322.5864068305	1.07365289298412	0.0138366825828019
PFKP	46.4929768369819	1.48330087290251	0.0459132693690945
RASGRP2	195.997908845571	-1.10459096357444	0.0457779717529985
KCNAB2	374.749245988332	1.83401448905355	0.0103850146392027
LMCD1	57.5281559492406	1.06148042783556	0.015889114721459
EVC	202.971290819002	1.49963875638329	0.0428164876502561
IGF2BP2	58.8373777475369	1.66125400438036	0.000274068718191157
CA12	81.4990740812502	3.81113397274314	0.00100743593241414
SLC12A1	9.4912022475983	-3.22992001131789	0.00106400310315629
MCAM	154.746133108518	1.66492951675722	0.0030844140288367
CAPN6	25.7245070976755	2.57623607046331	0.00017245513278913
LXN	26.7829499672959	1.11555364360328	0.0414718509567788
MOXD1	27.0426238732717	2.29546909975761	0.00640417735103604
GRHL2	21.251223493569	2.44045950032557	0.00422497003299876
TTC39A	19.5587648951474	1.36291656855599	0.0243355551232263
PTHLH	3.5346941669612	3.1258279738989	0.0259270057926525
TESC	39.6753972156991	2.17614137794829	0.000301168748959087
SLC26A3	51.5146861925741	2.579470773864	0.0312975677988687
SEL1L3	285.367397479363	1.069664324964	0.0098742846633194

SLC7A8	113.545082513508	-1.2140976528748	0.0300113422931293
TGFB2	19.2197916359792	2.40617214093687	0.0051539398427581
BAMBI	195.099554174906	1.219152262725	0.00580256742107098
GADD45B	883.992078465281	-1.36767781131579	0.0170304921618695
SLC5A1	96.9461678655945	1.93868631018605	0.00582804038374654
RAB36	12.2627302990534	1.46629492859641	0.00150439781754773
PVALB	20.0338925820189	-1.99510061462099	0.00632935922343394
UPK3A	5.16842232869413	3.21380966844653	0.00481231192534056
IL2RB	172.322765245668	-1.1828522473503	0.00481231192534056
TRIM9	70.1889140952072	2.75953935084994	0.0278807281060706
FERMT1	14.4480663857714	1.56734824702434	0.0133283150988447
JAG1	256.558871065499	1.41641594110788	0.0113578674616
LAMA1	20.0926689637424	2.05814630734097	0.00453914872692047
KLF5	31.6555217512223	1.79442568152322	0.00932816435671946
RGCC	19.394646523958	1.77351057137454	0.00180575762272094
RHOV	3.78402127399666	4.14316463478596	0.00112877693793146
NIPAL2	298.41918299032	1.14233970579664	0.0428164876502561
ESRP1	22.3138756594283	2.96947259158155	0.000190430625450584
TUBB4A	22.3471926678759	3.36526064621246	0.00432955555923546
CLEC4M	848.920161722724	-1.48537480963343	0.0405601786791036
APLP1	5.30449306729883	2.28338671429657	0.0439165864549174
ITGB8	29.7700622769209	2.23144971649875	0.00432955555923546
WNT2	32.8636427310125	-1.74505643771252	0.0030844140288367
NPTX2	9.62965827166842	2.28099968479639	0.00010972240660449
GLIS3	117.447804577222	1.07937567093929	0.000576738884052627
LGI1	87.0148182808396	-2.21086876236801	0.00432955555923546
KRT23	31.9040646868593	6.44433455423202	1.055566537679e-10
CCL2	74.4731058458238	1.46705869777161	0.0184083632545901
RND2	154.11597205453	-1.79962691426358	0.00825076375194256
SULT1E1	867.301872810941	-1.0540593673883	0.0437897070425335
MS4A6A	872.201217197026	-1.00135325909988	0.0249987913529739
VWF	422.228116416635	2.22083996694831	8.11948860429638e-07
SYT10	5.66402832519141	-2.21992135086148	0.00191202906015148
AKAP3	35.4861500843647	-1.20430449235509	0.00542262729805956
RASAL1	6.75767456731976	2.43171967225187	0.0034596658758818
PERP	2554.8063773124	1.05161591303283	0.0030844140288367
ARFGEF3	18.8381666362298	1.56001324870948	0.0378120764537426
MDFI	5.26386280500746	1.44161062449007	0.0389309643321594
ENPP5	21.2379518790502	2.15876516546056	3.34407532579434e-05
CDH6	79.899737775005	3.45653499062753	5.27280140732182e-06
DBN1	69.648953329083	1.45233978277631	0.00123909456152555
LRRC31	128.229369121918	-1.03730551318859	0.0318563223610417
C3orf52	8.78598729592234	2.39190407024375	0.0110404412403313
ITGB6	10.7080373287067	2.43771762613553	7.68602748320055e-08
CLIP4	76.6578809992056	1.99662687990729	0.00293915918529691
GNLY	102.544384008063	-1.05924611792409	0.0412761019867418
PLEK	116.136734869643	-1.16081068634345	0.0232382282203324
RGS2	72.4924460510633	-1.07436032435649	0.0233004564834127
RGS4	35.6422141792003	2.74452005358152	0.00559261786876192
MUC5B	121.482888122141	4.91199231756705	1.98653836294689e-06
C1orf198	236.399794466022	1.23061931064125	0.00144116367888336
EPCAM	118.791236917673	3.47993702169548	5.3146446381688e-09
SFRP5	231.396053413547	2.13014363720182	0.00144116367888336
MTHFD1L	71.6191728277431	1.1563052848102	0.0152767163956546

TNFSF11	16.0290576897159	1.34021412218905	0.0250617882312635
EGR1	348.845021504932	1.94303803670496	0.0287505088820584
DUSP4	20.8092476773847	1.44601396829712	0.0158752642106027
DPPA4	14.4878664199243	-1.5841549977971	0.0182154902826885
SLITRK3	50.0610564224719	-3.66332427171644	0.00404843348648364
AKR1D1	7057.38610879044	-1.02373102886345	0.00100743593241414
BICC1	151.874414635224	2.64867522694715	2.67049408802529e-08
BHLHE41	18.5064626878415	1.93853404042077	0.00281405758548834
ITIH5	91.0881114902835	1.74182149210598	0.0162000350184996
IL13RA2	75.9542591173228	-1.43460604623915	0.0158448998322581
ZNF831	15.0628688553483	-1.21091283977868	0.0133377594372195
PMEPA1	84.8715388888103	1.97846650749119	0.0014766262011178
POF1B	17.9500187664721	2.62381931700134	2.46105204118366e-05
SOX4	113.832893209089	1.85286904938975	0.000848963706513276
CXCL6	59.2735048812414	3.45276205035066	1.11974582565716e-05
MYRF	683.291918751718	1.15829751481604	0.00609207158992444
ABCC4	106.149430303538	1.56605321973715	0.013535186573776
SOX9	135.149058128648	2.32872835102171	1.19297217759789e-06
SDCBP2	203.929238348985	1.93316640470917	0.000238869507890472
FFAR2	4.69822643394887	-2.12760032256322	0.0155604187503747
GLIS2	63.1104088371323	1.36623302146514	0.0212727837334848
AIF1L	71.2808595263687	1.85896426949124	0.00100743593241414
ATP13A4	23.9289231988848	2.44836333190039	0.00551329508444726
LIF	19.6913229071714	1.86496384042434	0.0430410608322617
APOBEC3A	6.67870316332191	-1.9971835100411	0.0314190177282974
PODXL	233.385484654723	1.35088852162055	0.00819399936198138
LOXL1	23.5522050897924	1.83300850915256	0.040800537219139
AP1M2	32.064819679853	3.35647611177966	2.54464653987023e-06
FOXJ1	2.56224443458611	2.82348415577464	0.0428164876502561
ACE2	162.280233974437	1.45537048489443	0.0051539398427581
PLVAP	163.764499325707	2.21459143075861	7.41509620540167e-06
STK33	7.91193433609077	2.06384322229834	0.0381104889837708
SLC6A8	39.2830186191414	1.78057868283452	0.000783727542836514
EDA2R	23.066458815281	2.78332053763417	0.00135459449026624
GALNT15	43.9800761115508	1.93796984905335	0.000936734324309568
G6PC	8331.58384402622	-1.17231821836811	0.0462692498716562
MAP1B	50.7246600916965	1.52073453672494	0.000407252060612292
LGALS3	323.535743021319	1.76119897370354	0.0249987913529739
RHBG	408.547771348027	-2.07101733575314	0.00842206070849562
RAB25	22.5173011650079	2.75087461292229	8.16306946934542e-06
KANK4	132.008977788563	-1.88582063112856	0.0141336435239488
CHI3L1	1631.63453347042	3.07808802625336	0.0084129154964436
POSTN	140.664715968913	-2.01695451690739	0.0011205224833021
BEX2	19.7480716721464	2.14228548875203	0.000891470775464358
FAM83F	115.707901400542	2.0865989685762	0.0337359259141619
PTPN22	19.5874322623027	-1.02860052624984	0.0404584384867764
VTCN1	63.993997322709	3.48043422226593	3.17597506228603e-06
EMP1	97.5976191544653	1.5055872219038	0.0178756357498322
LRP4	22.1394151296128	-1.85160117301385	3.85980426232745e-05
DTNA	132.976673710269	2.49827263916898	0.0030844140288367
CLDN10	86.9139645291705	2.28659936683511	0.0011205224833021
GOLM1	489.864080635488	1.51614381243484	6.55469323607001e-05
TES	112.054611666529	1.01443777201495	0.0321883402287623
AGAP2	19.1325296773105	-1.00767862790203	0.0297604124593965

KRT87P	3.74875939323019	3.1414813253456	0.0320262237216149
KRT7	270.88212646969	2.99020820614743	5.56346254377866e-08
KCNH3	13.852629585378	1.58770528790664	0.000218073354201783
PCNX2	24.872139551175	1.3397509274904	0.0138366825828019
NACAD	4.40608032949385	1.85830164050558	0.0199122952052687
LRRC1	27.0382304190859	2.21934810666234	1.0106569813408e-07
FGFBP2	9.55120805198759	-1.5867194857175	0.0167334898999925
MMP7	70.0100115135461	3.52231707278672	1.92137708021331e-05
FXYD2	21.7111312625585	1.83579873545454	0.00641610437425617
PAQR5	40.8567718366731	2.08552677223154	0.000912493021418272
SLC44A5	39.327734400206	2.56472258156113	0.0119371554539066
LOXL4	134.598358403672	2.72015184218308	1.79742007358844e-05
CH25H	6.36648183199183	2.12660756847687	0.0167322315388678
ANXA3	49.7288641894889	2.14587333480769	0.00276953180104953
LGR5	68.7272578279518	-1.74394707009969	0.00609207158992444
PDX1	12.0279485436482	3.86751776909744	3.55139650692923e-10
ITGB7	53.4310461856919	-1.05296012230236	0.0227178205471219
CDH24	18.4180989007269	1.17271860759729	0.0314190177282974
RDH12	25.3928606747335	1.99273552281282	0.0408480755356006
TPM1	895.649057181195	1.01785343055298	0.000691768336020804
CHST4	130.911601053616	2.29633514123295	0.000211770285476572
PLPP2	56.2381531469487	2.60512626475309	2.52509702421378e-06
SIK1	18.3492810123153	-4.49023014576764	0.0167334898999925
LMTK3	6.56762863833589	2.04669652195219	0.0428164876502561
CNKSR1	48.7240097738705	2.34251202323256	2.36410071749572e-09
IGSF3	62.3709334748936	1.64158676030097	0.0285791890560433
KCNN3	17.4941326480817	1.76785538321193	0.00905971810371764
ILDR1	14.8090898970221	1.92013956603932	0.00300971518681784
SCD5	62.7381746684434	1.87939865796819	0.000288656335607061
NKD2	30.0181598757526	2.4054535025151	0.00609119130522683
N4BP3	6.83742489468198	1.94447568974889	0.0167250815282735
TENM2	172.346977467918	-1.92520613298517	4.06594109645072e-06
DCDC2	178.540123027816	2.99732904676201	1.15664899931939e-08
GPC3	187.845873905032	4.24839973023085	2.32472366575292e-07
RSPO2	6.30199785141297	-1.94121019790573	0.00839750595380541
CDKN2B	18.748704011878	1.76951132445228	0.0202114433626093
CDKN2A	12.4389842351509	3.19062814335691	1.03664770992397e-07
SLC25A25	1113.28442596917	-1.05623276044506	0.000967190608896738
ANKRD1	6.91907612355167	4.26400718527473	0.000420993901394291
TENM4	26.6798411046478	3.42640779259204	0.000407252060612292
ST14	324.474229540643	1.56498841090422	0.00265740477102986
KLRF1	20.7606531767704	-1.69234532252702	0.000131288052812213
ADRA2A	16.7870487365538	2.51446377363624	0.000211770285476572
HSPB8	54.6610528179813	2.30223575057668	0.000174169071927295
CAMK4	50.199407830084	-1.22485055129566	0.00432955555923546
KCNJ16	36.5701082397949	2.15502867946187	0.0281124596557948
HS3ST3A1	11.4315765792697	-1.80807674332036	0.0121861301065523
CABYR	6.25893577066717	1.72671515751886	0.00798254909073145
CHST9	359.699021885274	1.27602793419875	0.0188244561715107
THY1	64.0329134505905	1.79518353977381	0.00275108668890254
TNIK	94.7636547232015	1.510592899715	0.006018021923541
NCAM2	360.857929481584	-2.58945921303705	0.00111669869872824
SLFN13	90.21186014795	1.38124396073847	0.0459653960939531
APCDD1	69.9463432531852	2.13012614361304	0.0246009943736072

HKDC1	292.14117362158	4.16197299114733	0.00100743593241414
B3GNT7	24.5791982591513	1.44156926071535	0.0450505085663366
SLC34A2	45.5886127435572	2.39534156686705	0.00112674115958887
RHPN1	56.1315712662335	1.0996639749254	0.00649244532455086
NPM2	42.5191063223894	1.88672333971341	0.00300971518681784
STC1	12.9452309027455	2.21465423292732	0.0128761393458746
CLIC6	80.8222935983723	3.90987114619447	2.67049408802529e-08
TUBBP5	13.2258630278127	2.20224202906223	0.0167334898999925
TPPP3	32.3407057824836	1.71568097645499	0.000187416073431572
ZNF233	10.2120985099774	1.19430467923913	0.0264543226196962
TMPRSS3	34.9604367391879	1.80738020841205	0.000190228330270655
FCRL3	19.2352562407679	-1.31697051836582	0.0233830015628059
CYP3A7	1687.06824133455	4.07416709544267	1.51759952480241e-32
DMKN	45.1454260417267	1.38800520970579	0.0143841850225964
BICDL2	16.1211538139145	2.24208822007416	0.00288617702502002
PAQR4	10.5116671263937	1.37167654299249	0.0254323530355539
PDZK1IP1	100.767995718006	3.91570490975977	6.23666247764746e-10
RBP7	34.7734111811087	1.62378617427301	0.0252148934991824
VANGL2	15.8587279757996	1.33225408051525	0.0481400419624993
FCGR3B	18.6224483860033	-2.4896765703469	0.0201972235737074
FCMR	41.6634951230528	-1.02753217955464	0.0463397316186124
CAPN2	1159.40868899413	1.71500240084142	4.58705048914487e-05
CFAP221	27.3517664090116	1.48111859118732	0.0356961035817483
SGPP2	12.8051337465279	1.94068873885236	0.00382917857248763
MSX1	8.03299023606069	2.43371122780529	0.0218743063404119
C1orf106	14.3441766098807	2.66247085943241	0.0018773796283953
SLC22A15	30.2961938430104	2.64084572656995	0.000311169642624131
CXCR1	7.80377041773006	-3.49596632130543	2.6497441837039e-06
ADORA1	12.9473425682977	1.87289051759139	0.00978559145720976
CD200R1	27.1231476923504	-1.04910479288536	0.0304342953126775
CDS1	22.9423461899464	1.76321812683843	0.0064876260201619
CXCL5	4.12581002323635	2.93878230695996	0.0113578674616
LIPH	11.4325020054119	2.16168799641677	0.000312011722617842
MELTF	39.3117902603292	1.30899968038144	0.00979579818716153
HSPA4L	239.260588399495	1.18079636218087	0.040482681771472
NDST3	11.9743196503147	-1.25607472377574	0.0367200838917854
ELOVL7	28.2397278705085	2.27861704386459	0.000153734707388776
F2RL1	114.616890707237	1.34596439689038	0.00971051447101889
CDC20B	2.08855721947496	3.62190147705162	0.0250617882312635
ANKRD55	38.3185192298889	-1.01829828239268	0.00655654407497125
NEUROD6	5.76957360109236	-1.92931027733888	0.0246247824486426
ADCY1	3015.11518592518	-1.24937382839212	0.00150103734898597
DIRAS2	9.66394433734045	2.33034621304709	0.00802104581970176
TRPV6	36.7677254484637	2.04797603612547	0.00458501429160891
WNK2	93.2700973360636	2.30175844992689	0.000702561967553672
SLC18A2	10.5146201613699	-1.47729846326369	0.0323289214077222
FRMD7	16.0336764949393	-1.74559047257965	0.0475722529832353
TC2N	89.8695406563552	1.64762842469497	0.0213939185481315
SERPINA12	23.1660323330146	1.84524564657229	8.28471150222208e-05
SPINT1	121.127635381052	2.90398684274081	5.77250993253188e-09
AVPR1A	536.249032927358	-1.96917620110797	0.0493936369290227
LARP6	62.1548850668966	1.49957348792564	0.00144116367888336
SERPINB8	140.474028744239	1.19902918388396	0.0018773796283953
PRKCB	66.9864578537001	-1.17526829030645	0.0283736619858364

ACSM1	235.361774083964	3.2048299867035	0.000137129437320616
LDHC	15.029599246359	-3.05885784014164	0.0138682000737234
PRR15L	48.154181446739	1.60601993535184	3.77668358978971e-05
GPRC5B	174.818796233463	1.55249660828712	0.00632622442174225
CACNB3	27.0333548203108	1.11524990843021	0.0188244561715107
TMC4	123.541552540364	2.5918816276229	0.0229569191686067
SPINT2	170.498992900721	1.77267233803538	0.000691768336020804
TMIGD2	5.38495834275817	-2.15768660317907	0.0475761980232135
TRPV3	32.6310196377814	1.97598748591612	0.0233830015628059
GGT6	17.4939085516002	2.20686550484609	0.0167322315388678
KRT80	15.384203396269	3.58008823867684	3.85697240142595e-07
FILIP1L	61.9034657661441	1.47345822166823	0.00170083927469687
BDKRB2	16.171139355695	3.12875306226143	2.6497441837039e-06
NPNT	97.6422249647255	1.57467149919878	0.0404470943770781
MFSD7	193.28413591076	1.42140564033137	0.0182154902826885
CXCL8	33.0676667263316	3.30111566716251	0.0020881563310238
GPR183	19.967155050269	-1.127088561633	0.0290199700510326
CTNND2	32.7371784232079	1.91369620924636	0.0391110189590975
ZPLD1	7.82418282294768	2.54510857355321	0.0462434834791338
SERPINA9	5.74663507075758	1.83210339657191	0.0182154902826885
SEMA3E	12.8670242666986	2.43663143088534	0.00203141951634407
PDGFD	92.7339696655831	1.87273745499261	0.00265740477102986
CDC42BPG	53.1662001412751	1.02087584282704	0.0224359652488166
KCNK3	13.3715007294084	-2.31833642506816	9.57712459232721e-05
KRT19	109.045910593279	1.58421559766842	0.0351337049417762
NMRAL2P	2.70931245300819	3.6623347093524	0.00736520068953118
LGALS4	3556.2926685393	2.17874460431091	0.000205442545474132
ID4	24.1829972612084	1.73015057197744	0.018240899465426
CXCR6	31.2924570759311	-1.21905010256794	0.000190430625450584
ZMAT3	476.470358546244	1.14234186647571	0.0409103150052263
OVOL1	5.65677090275559	3.4614871625576	0.0084129154964436
EVC2	20.5517799798371	1.35531735315639	0.006018021923541
TNFRSF10C	18.6584576458778	1.66909882387277	0.0227821604592961
TRAPPC3L	2.91905846450316	-3.19360151891037	0.0166374089363057
MUC13	12.0947001828063	2.77382774920228	0.0167250815282735
C11orf80	46.4087241834896	1.25556350326975	0.00905460395833625
HAP1	12.7256675651183	-1.81803568362595	0.00606154277413209
CBX2	22.722512519823	1.37987100762411	0.000174169071927295
CD34	132.378307047735	1.60219518796443	1.0605677257676e-05
PHLDA3	134.74177183553	1.50561449347081	0.0145961075081613
SLCO2A1	103.483987282796	1.62151298846683	1.79742007358844e-05
SEZ6L2	34.9154688833304	3.52885689033166	1.16531647905751e-10
ASPHD1	10.5474657601908	2.32203825073915	0.00234382487208137
CADM2	10.6827479168621	-1.58177526796777	0.0204391548681969
CLCF1	14.1517832571113	1.58011838166206	2.79700656622325e-06
ETV4	23.4101644136363	2.29364451333381	0.000190228330270655
A2M	48555.8090720097	1.9003837716149	0.00770605137475211
SPHK1	38.9172931345356	1.94709110922283	1.11974582565716e-05
GCNT4	49.4413365352155	1.65600739041472	0.0059483407919081
ERICH5	275.843746259717	1.50957669534836	0.0175160008109977
TMEM125	18.8962546651319	2.25222080533715	0.0002823200517001
B3GNT3	55.970185181497	3.30305338943673	5.36890763592517e-07
TMEM150B	42.3443945519295	2.26293104088348	0.00559261786876192
PRR26	53.7413706091044	1.04748102334193	0.0027402060544844

PRF1	88.9427984973331	-1.18652866316514	0.00288617702502002
S1PR5	15.2347315014194	-1.61427003972749	0.0076289543246958
NQO1	60.1297461266686	2.56394450545357	3.85980426232745e-05
MAB21L2	23.5731920079204	1.75510893242981	0.017802909543641
C6orf223	2.89652328758261	3.55186143638918	0.00205530857572877
TIGIT	18.8911747231124	-1.23967061681955	0.0224359652488166
CLDN7	312.410297825051	1.17662616164442	0.0246009943736072
LDOC1	30.6133631534872	1.07907537637686	0.0217021873995268
BACE2	130.87372784978	1.59856084824656	1.11974582565716e-05
ANXA2	1718.03887058873	1.85883657816179	0.000537628569965476
C1orf116	43.267498887363	2.49441079487337	5.62107719892863e-05
MACC1	14.3500554937651	2.34762622588452	0.00404796741325496
B3GALT5	33.9486118248473	2.34261267189738	0.0064876260201619
FAM3B	28.2182777449149	2.33362545091002	0.00125745304025186
LRRC55	60.4566843511476	-2.7749400390224	0.00149264398537461
PRKX	92.1871314894785	1.05315493866298	0.00909347498297562
ADRA2C	7.37860606512618	1.76281866114793	0.0249987913529739
TACSTD2	74.5266675697977	3.30971218054287	9.70745076520113e-12
PKP3	17.0378387368375	3.60361391976998	2.21112630486719e-05
MUC6	159.84410536703	2.49893176126082	2.88399462836292e-06
MUC1	27.8329079077513	2.68390964362566	0.00828343598520972
MYBL1	39.8138660941157	-1.00636718936458	0.00149264398537461
CTD-2600O9.1	8.14457480120482	1.67428796517939	0.0212076146064624
KCNJ11	10.2959937901769	1.34015032841868	0.0167334898999925
FAM69C	4.82687956031115	-2.3019934358275	0.0212076146064624
ZCCHC16	31.8475984406533	-1.37807955102661	0.0206225480614195
C6orf132	19.1972718355066	1.93313098048023	0.0250764420402524
SELL	58.4368311351513	-1.06292594381106	0.0108683883805256
COL25A1	34.2846060661438	-1.15730334535238	0.0184083632545901
CLDN4	60.2023378256625	2.18010920694708	0.000268498183231101
FAM150B	20.2410742213558	1.76581007630888	0.0208332181088841
KIF19	37.2853722661503	-1.05723008190392	0.0287505088820584
SFTA2	4.45000020250681	3.63785612125317	0.0316325373474369
SRC	237.625644588053	1.08800841668622	0.0101082821407059
ADGRA1	15.1987896096287	-2.64061456327214	2.6497441837039e-06
VEPH1	25.989057974463	2.49915761464664	0.000185125625702886
PDGFA	84.800506404564	1.29204252825083	0.0167632508683847
SLC28A3	22.0722405513318	1.96141532062135	0.00276953180104953
CR1L	6.55086869666096	-1.79226326011592	0.0190362712954183
PTMAP2	13.1029701767519	-2.85910494939105	0.0450505085663366
S100A6	403.414875283625	1.41194532345975	0.0339022265046587
AKR1B10	1259.98574684702	5.19860322624065	2.91673528742692e-08
SULT1C4	33.8329564736917	2.0112785608967	0.000591476557368028
SULT1C2	177.108925726975	3.35170775720995	0.00432955555923546
PLXNB3	28.3704412270167	1.60091340796152	0.0327146077307638
RP11-163F15.1	8.07521369206726	3.81471782633397	3.63732358748245e-05
FOXO6	5.74120921067184	2.73818966118391	0.0212076146064624
GPRIN2	10.9348156483118	2.14881629240991	0.0290199700510326
COL15A1	28.6861775048506	3.05484687338102	0.000756548420221007
LAYN	13.4514474390093	1.85963880751812	0.00369386031568281
NCR3	9.16821248844234	-1.92041164219053	0.00400064912913566
PSORS1C3	12.6269420396467	1.90233966436206	0.0167250815282735
NAT8B	46.1141534087114	2.03260846330064	0.000186476713306085
ADGRG1	112.157227794878	1.77292227261987	3.61018822115176e-06

SLCO1B7	8.67631183693816	-1.53381352331243	0.0331525124947241
CYS1	55.8181024087922	1.32172238534021	0.00495154037194302
RP11-473M20.5	3.58770366181201	2.59994532440938	0.0140793972501447
DOK6	56.1494538677904	-2.37355378379383	1.11974582565716e-05
TRDC	17.5251828287832	-1.59685442993819	0.00432955555923546
HNRNPA3P6	15.1006535142831	1.00720406282526	0.00349596603688623
RPS7P14	7.07998042845581	-1.85194221126916	0.0481400419624993
AC098614.2	11.8669792464292	1.91029113753828	0.00256475499622181
TAX1BP3	11.8320635818049	1.83616052388111	0.0327708975963136
UCA1	16.6652460933323	2.99388627456121	0.00165388237508148
COL28A1	34.6390368382426	-1.60717558925102	0.000808378223708392
ATAD3C	172.458405161439	-1.70253522237402	0.0385328725554505
RP11-465B22.3	22.6123950530654	3.69556425649595	3.77668358978971e-05
TDRD15	33.6961628093778	1.44673191272292	0.0285791890560433
FAM19A5	43.7306361698797	3.67829182873725	0.00263337601118412
PPP1R3G	48.9118083964407	-1.42956858662328	0.0279156838638082
FXYD7	4.87246383437442	-2.12226904443334	0.0110220395089333
CYTOR	7.13472465304243	1.79127090562487	0.015624415409214
RP3-395M20.2	9.63681729951276	2.8312600061987	0.0428164876502561
AP001626.1	5.9055442967529	2.07095200539748	0.0249987913529739
SATB2-AS1	2.70279616550456	2.6822714598245	0.0259181332209531
NPY6R	62.7950543932824	-3.0723702374125	0.000635672940337574
CROCC2	34.1065949521483	-1.03542523231329	0.0123126598225338
RP11-289F5.1	8.43185359276993	-3.7543611674887	0.0169049801711688
HORMAD2-AS1	30.879660059516	-1.36475699275344	0.0198047036434074
MEOX2-AS1	3.07047508629508	-3.13042588125311	0.0405773474729345
RP11-367G18.1	7.24313527747639	1.41332562036293	0.0250617882312635
AC013275.2	6.35471976689444	2.33686097242912	0.0162575867271289
RP1-27K12.2	56.8492464561322	1.61208936017501	0.00258745032730043
PIK3CD-AS2	4.23399912480022	2.04465915964753	0.0459653960939531
HS1BP3-IT1	50.4141543644466	1.04634992795912	0.0155604187503747
AC016831.7	12.3549282983167	1.93617401986097	0.0386148265187823
RP11-255H23.2	10.3438471006139	2.18671597113784	8.14014681362477e-05
DPP10-AS1	5.11469487906772	3.43629889340516	0.0435900571581891
RP3-417L20.4	2.60434976388868	4.23038127041578	0.0459168154350462
C12orf75	33.9893638435778	2.06350745615055	4.54149597540626e-06
AL162759.1	4.59553724813656	2.87779485398159	0.00552885059502126
RP11-439H9.1	7.45826313449111	-3.28141296730994	0.0133648855951827
RP11-59D5__B.2	4.11714814729297	2.05337422796687	0.0144880932042998
ARHGEF38	6.26795281210967	1.67362235002981	0.0402168114494961
AC005550.3	12.6240140881412	-2.92203699953352	0.0167250815282735
RP11-278H7.3	8.71105015724727	-3.49797923307226	0.00713468839547332
RP3-395M20.8	167.140503284087	1.21993036301373	0.00432955555923546
GATS	46.2690252758026	1.20805237983573	0.0120627087443952
AQP1	796.381774002583	2.33473668057738	8.66563444501805e-08
LILRA6	19.0250332883082	-1.40786667208577	0.0407834791376932
PTCHD4	7.60007152854065	3.67108086646054	2.32472366575292e-07
LINC00861	25.2206991170647	-1.25915018276663	0.026588573140817
LINC01550	10.3431603417752	2.78973783138949	0.0239890221581195
CCAT1	12.3640086223785	2.79086761008397	0.0098742846633194
PCDHA11	3.86616280720519	2.32329375844963	0.00993464473283081
LINC01021	29.7842232330075	2.6549919015747	0.0155604187503747
CTD-2005D20.1	18.0863834368643	2.9256231790172	0.00955536283373315
TRNP1	64.0446362541457	2.59858846962653	0.000363395389786314

KB-1615E4.2	5.91710384093901	2.29777145974479	0.0449202886206357
RP11-150O12.3	5.55089371667177	3.28723661097312	0.0263235010898177
RP11-626H12.1	17.2954094276102	-1.32287275314516	0.026588573140817
RP11-575F12.2	9.92044743895397	-1.28840410030493	0.0413499757674839
ADGRA1-AS1	3.64681103159751	-2.66868187135587	0.0463397316186124
KRT17P8	283.029798097511	-2.24840181290957	0.0067505709028866
MGAM	6.83173272606938	-2.17128905839766	0.0316718817400346
RP11-44N21.1	11.0637347206739	2.45457384002552	0.0290199700510326
RP3-416H24.1	6.25102075264105	2.49508819308651	0.0051539398427581
RP11-25E2.1	11.5525351146803	-1.65654523446589	0.0151790249257851
RP11-510M2.5	13.9847633414542	1.73697925814988	0.0405601786791036
RP1-239B22.5	8.10061130045011	1.48161625278702	0.0405601786791036
RP11-114G11.5	48.2674484582793	-2.90241815377775	0.000137129437320616
RP4-555D20.2	5.17574382424085	3.07884932140233	0.0486110083705687
RP11-353N14.4	3.234701729639	2.89889638163957	0.0314190177282974
RP11-353N14.5	29.4973608766557	4.33251295004476	0.000185125625702886
GJA5	32.1052898721038	1.61307557630264	0.00955536283373315
RP11-484N16.1	9.81002433055178	2.49311361597493	0.0170955675492222
CTD-2008P7.9	15.5819786552878	3.38185038104782	0.000184363280707364
LINC01482	34.6978146815048	-2.15334573074272	0.0140234132088284
AC004156.3	12.0839761356464	-1.47296177883282	0.049929857576501
RP11-120K24.3	19.7029990728073	1.19311206254778	0.0232382282203324
RP11-465B22.8	10.9562518491914	2.07468322911156	0.000367648192977179
CD24	523.70812706959	2.82404623715818	2.3804977305159e-07
RP11-284F21.10	8.33405605911352	3.49751856032156	0.00609207158992444
RP11-468N14.13	7.61182933692702	3.66133562765461	0.00432955555923546
RP11-54O7.18	4.20933702665961	4.67196700717462	0.0132050038443973
RP4-568C11.4	163.831473389198	1.34935625860033	0.0249987913529739
PI4KAP1	131.400760016476	1.05455885019877	0.0345933136021904
RP11-569G13.3	6.91090464227913	2.67183775868181	0.00432955555923546
RP11-96A15.1	7.41326736538114	2.11176220131674	0.0387120965413674
HIST1H3H	24.7190464961488	-2.16267480258624	0.00312763116945927
RP11-114G11.4	6.11421554138446	-3.2863478096481	0.00432955555923546
RP11-437J2.3	5.81399535845764	-3.16291986782852	0.00609207158992444
CYP3A7-CYP3A51P	38.8467796908418	3.71399674806295	2.36410071749572e-09
BISPR	90.3193289382196	-1.60461584490272	0.0231528039663219
RP11-403F21.4	11.5519788923604	3.33357695850581	0.00428056428386065
CTB-76P12.1	11.8732265299321	3.79749841839338	0.00265740477102986

Table S4. Baseline Demographics and Clinical Characteristics of the external Study Population. All data are presented as mean (SD) or n (%). Serum bile acids, C4, and FGF19 data are available for n = 40 subjects.

*UDCA and UDCA conjugates were removed from the BA pool.

Abbreviations: PIII-NP, type III procollagen peptide; TIMP-1, tissue inhibitor of metalloproteinase 1.

**Characteristic**	**Total (n = 74)**
Demographics	
Age, years	46.3 (11.3)
Female	22 (30)
Body mass index, kg/m^2	26.9 (4.8)
UDCA use	50 (68)
Ulcerative colitis	37 (50)
Ethnicity	
European descent	60 (81)
African-American	12 (16)
Asian	2 (3)
Liver tests	
Alanine aminotransferase, U/L	85.1 (72.7)
Alkaline phosphatase, U/L	332.6 (302.4)
Gamma-glutamyltransferase, U/L	408.6 (457.4)
Bilirubin, mg/dL	1.1 (1.3)
Albumin, g/dL	4.0 (0.4)
International normalized ratio	1.0 (0.2)
Platelets, ×10^3/uL	260.5 (98.0)
Other biomarkers	
ELF	9.47 (1.35)
Hyaluronic acid, ng/mL	100.8 (188.2)
TIMP-1, ng/mL	331.3 (163.7)
PIII-NP, ng/mL	9.6 (5.7)
Hemoglobin, g/dL	13.9 (1.7)
Total bile acids, pg/mL	1590.2 (586.2, 3649.2)*
C4, ng/mL	8.6 (4.3, 24.6)
FGF19, pg/mL	135.8 (60.4, 221.4)
IL-8, pg/mL	19.5 (12.0, 41.0)
Liver histology	
Ishak fibrosis stage	
F0-F1	26 (35)

Characteristic	Total (n = 74)
F2-F4	37 (50)
F5-F6	11 (15)
Hepatic collagen content, %	5.3 (5.1)
α-SMA expression, %	5.3 (7.7)
